# Enhancing chemical and biological diversity by co-cultivation

**DOI:** 10.3389/fmicb.2023.1117559

**Published:** 2023-02-01

**Authors:** Denise M. Selegato, Ian Castro-Gamboa

**Affiliations:** Nucleus of Bioassays, Biosynthesis, and Ecophysiology of natural products (NuBBE), Institute of Chemistry, São Paulo State University (UNESP), Araraquara, Brazil

**Keywords:** activation of Biosynthetic Gene Clusters (BCGs), enhanced chemodiversity, microbial co-culture, untargeted metabolite regulation, microbial interaction

## Abstract

In natural product research, microbial metabolites have tremendous potential to provide new therapeutic agents since extremely diverse chemical structures can be found in the nearly infinite microbial population. Conventionally, these specialized metabolites are screened by single-strain cultures. However, owing to the lack of biotic and abiotic interactions in monocultures, the growth conditions are significantly different from those encountered in a natural environment and result in less diversity and the frequent re-isolation of known compounds. In the last decade, several methods have been developed to eventually understand the physiological conditions under which cryptic microbial genes are activated in an attempt to stimulate their biosynthesis and elicit the production of hitherto unexpressed chemical diversity. Among those, co-cultivation is one of the most efficient ways to induce silenced pathways, mimicking the competitive microbial environment for the production and holistic regulation of metabolites, and has become a golden methodology for metabolome expansion. It does not require previous knowledge of the signaling mechanism and genome nor any special equipment for cultivation and data interpretation. Several reviews have shown the potential of co-cultivation to produce new biologically active leads. However, only a few studies have detailed experimental, analytical, and microbiological strategies for efficiently inducing bioactive molecules by co-culture. Therefore, we reviewed studies applying co-culture to induce secondary metabolite pathways to provide insights into experimental variables compatible with high-throughput analytical procedures. Mixed-fermentation publications from 1978 to 2022 were assessed regarding types of co-culture set-ups, metabolic induction, and interaction effects.

## 1. Introduction

Microbial specialized metabolites are a result of several billion years of evolutionary biosynthetic optimization. Their biosynthetic enzyme pathways use simple building blocks to assemble architecturally complex metabolites, displaying tremendous potential to provide new therapeutic agents (Pettit, 2009; Newman and Cragg, 2016). Even with the challenges related to unlocked genomes and unculturable strains, they still represent over 50% of the clinical antibiotics and account for over 42 thousand natural compounds already reported in the literature (Bérdy, 2005; Van Wezel and McDowall, 2011; Laatsch, 2012).

The potential of the microbial metabolites is not only based on the currently available chemical structures but also on the unknown and certainly huge number of not yet studied microbial populations. Moreover, it has become clear that the microorganisms still hide yet undiscovered secondary metabolite pathways. Recent whole-genome sequencing of several bacteria shows that the microbial potential to produce secondary metabolites is fairly underestimated, meaning that a much broader range of compounds could be produced if the silent genes are induced by whatever methods (Scherlach and Hertweck, 2021).

Conventionally, in microbiology, single-strain cultivation has been the standard method for the screening of secondary metabolites. However, due to the absence of biotic and abiotic interactions, growth conditions in monocultures are significantly different from those encountered in the natural environment (Pettit, 2009; Onaka et al., 2011). In nature, microbial metabolic pathways are often regulated by complex signaling cascades, in which the metabolic pathways are controlled by regulatory genes and influenced by external factors. For example, in competitive environments, microbial species engage in constant interactions with their neighbors, competitors, and hosts, resulting in phenotypic and genotypic effects that ensure survival and shape the community in this microenvironment (Moody, 2014).

The absence of biotic and abiotic incentives is one of the most significant limitations of axenic cultures and limits the observable chemodiversity of microorganisms. Monoculture screening often provides an ever-increasing rate of redundancy, with chemically poorer profiles and the frequent re-isolation of known secondary metabolites (Hong et al., 2009; Marmann et al., 2014). In the past decade, several methods have been reported to eventually create the physiological conditions under which cryptic genes are activated to stimulate the biosynthetic pathways and elicit the production of hitherto unexpressed chemodiversity (Schroeckh et al., 2009; Wakefield et al., 2017). These strategies have been successfully applied to the genomic activation of the cellular machinery producing other classes of specialized metabolites than previously found in these organisms (Hertweck, 2009; Bertrand et al., 2014b; Wakefield et al., 2017).

Among those, genetic approaches have been very efficient methodologies for improving the yields of specialized metabolites and include genome-mining, direct mutagenesis expression, recombinatorial biochemistry, heterologous expression, and ribosomal engineering (Bergmann et al., 2007; Starcevic et al., 2012; Gaudêncio and Pereira, 2015; Okada and Seyedsayamdost, 2017). Overall, these genetic manipulations are pathway-specific and focus on altering the expression of transcription factors that control targeted Biosynthetic Gene Clusters (BCGs). As a consequence, these experimental protocols are usually more time-consuming and cannot be performed in strains that are not easy to genetically manipulate or that do not have defined genomes (Santos and Stephanopoulos, 2008; Imai et al., 2015; Romano et al., 2018).

Genetic-independent methodologies have been equally used to regulate BCGs and enhance the yield and diversity of specialized metabolites. These approaches mostly focus on the untargeted regulation of the metabolome, unbiasedly increasing chemical diversity without direct manipulation of the genome. Several of these strategies have been described in the literature and comprise mainly the use of substrate feeding (De Boer and Schmidt-Dannert, 2003), small molecules elicitors (Pettit, 2011a; Seyedsayamdost, 2014; Xu et al., 2017; Mao et al., 2018; Moon et al., 2019b; Xu et al., 2019), co-cultivation (Pettit, 2009; Shank and Kolter, 2009) and the variation of nutrient sources and availability by One Strain, Many Compounds (OSMAC) (Bode et al., 2002; Wei et al., 2010; Rateb et al., 2011; Pan et al., 2019; Selegato et al., 2019; Hebra et al., 2022). These have been shown to provide a broader expression of the metabolic pathways than their respective monocultures, thus increasing chemodiversity in a more holistic and unsupervised manner.

Microbial co-culture involves the cultivation of two or more microorganisms in the same confined environment and has been considered a promising strategy to induce cryptic pathways (Rateb et al., 2013; Wakefield et al., 2017). In a co-culture experiment, microbial communication occurs either by volatiles or *in-loco* signaling and leads to the regulation of specialized metabolites. The regulations of the biosynthetic pathways can be made by exogenous metabolites or autoregulatory molecules (Schroeckh et al., 2009; Pettit, 2011b; Rateb et al., 2013) and have been shown to give a pleiotropic metabolic induction without requiring any prior knowledge of the genome, nor any special equipment for the cultivation and data interpretation (Moody, 2014).

The first co-culture studies were mainly established to understand the natural or synthetic interactions between human microbiota and pathogens as well as to improve biochemical processes in the food (Islam et al., 2017), solvent and oil industries (Kumari and Naraian, 2016; Tao et al., 2017). Successful examples of industrially applied co-cultures can be found in wastewater treatment, soil remediation, gas production (Chaudhry and Chapalamadugu, 1991), and food products such as dairy products (Sodini et al., 2000; Martin et al., 2001; Narvhus and Gadaga, 2003; Kariluoto et al., 2006), salami (Dicks et al., 2004) and alcoholic beverages (Cort et al., 1994; Beek et al., 2002; Fleet, 2003; Clemente-Jimenez et al., 2005; Renouf et al., 2006). However, it is now well accepted that mixed cultures can also be successfully applied to improve limiting steps of a biosynthetic pathway (Zhang and Stephanopoulos, 2016), prevent enzymes from byproducts biosynthesis, increase single-cell protein production (Tesfaw and Assefa, 2014), induce or increase bioactivity (Chanos and Mygind, 2016; Sung et al., 2017), suppress virulence (Minerdi et al., 2009) and improve the bioactive metabolite production by the presence of a microorganism that alters media composition (Shimizu et al., 1999; Liu et al., 2006; Ariana and Hamedi, 2017).

**Figure 1:**
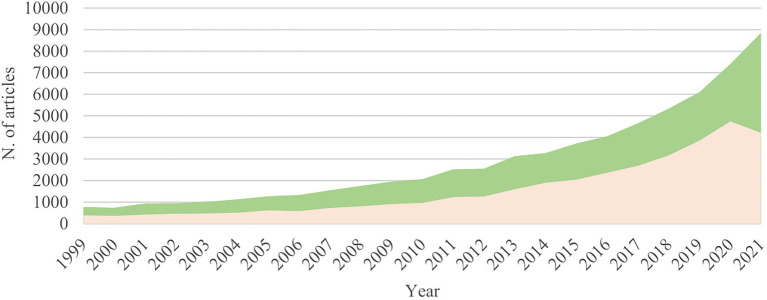
Evolution of the field of co-culture; Number of publications per year in the fields of co-culturing microorganisms to increase biological activity (green), and co-culturing microorganisms to enhance chemodiversity and induce the production of novel secondary metabolites (salmon). These numbers were obtained by a metric search from 1999 to 2021 (Web of Science/Science Direct).

Co-cultivation research that targets BCGs’ activation and the enhancement of chemodiversity is still in its infancy compared to the applications to the human microbiome and industrial processes. Only a few reports deal with the biosynthetic and biological role of the induced compounds (Figure 1). Most studies in that direction concern the microbes that induce novel anti-infective or anticancer compounds by antagonist interactions in an attempt to overcome multidrug pathogen resistance (Dashti et al., 2014; Ueda and Beppu, 2017). Nonetheless, other types of neglected microbial interactions such as amensalism, commensalism, cooperation, and mutualism have also resulted in sporulation, biofilm formation has been reported to occur in co-culture (Ueda and Beppu, 2017).

Several reviews have shown the potential of co-cultivation to produce new biological leads. However, these studies are mainly regarding the chemical and biological results reported in the literature (Burgess et al., 1999; Rutledge and Challis, 2015; Abdalla et al., 2017; Onaka, 2017; Arora et al., 2020; Knowles et al., 2022). Over the last decade, only a few articles have reported detailed experimental, analytical, and microbiological strategies for efficiently identifying bioactive molecules in co-culture (Bertrand et al., 2014a,b; Azzollini et al., 2018). Hence, in this review, we focus on the description of co-culture experimental approaches between microbes (for other interactions, please check (Zhang et al., 2022) and other reference works) and workflows compatible with high-throughput analytical procedures. Particularly, we have interpreted all major mixed-fermentation publications from 1978 to 2022, displaying the current knowledge on different types of co-culture, metabolic inductions, interaction effects, and how these protocols can be optimized to enhance chemical and biological diversity. Lastly, while numerous applications of co-culture have already been reported, we hope to shed light on this strategy as an efficient strategy for the activation of Biosynthetic Gene Clusters (BCGs) in a microorganism, exemplifying major challenges, significant results, and perspectives.

**Figure 2:**
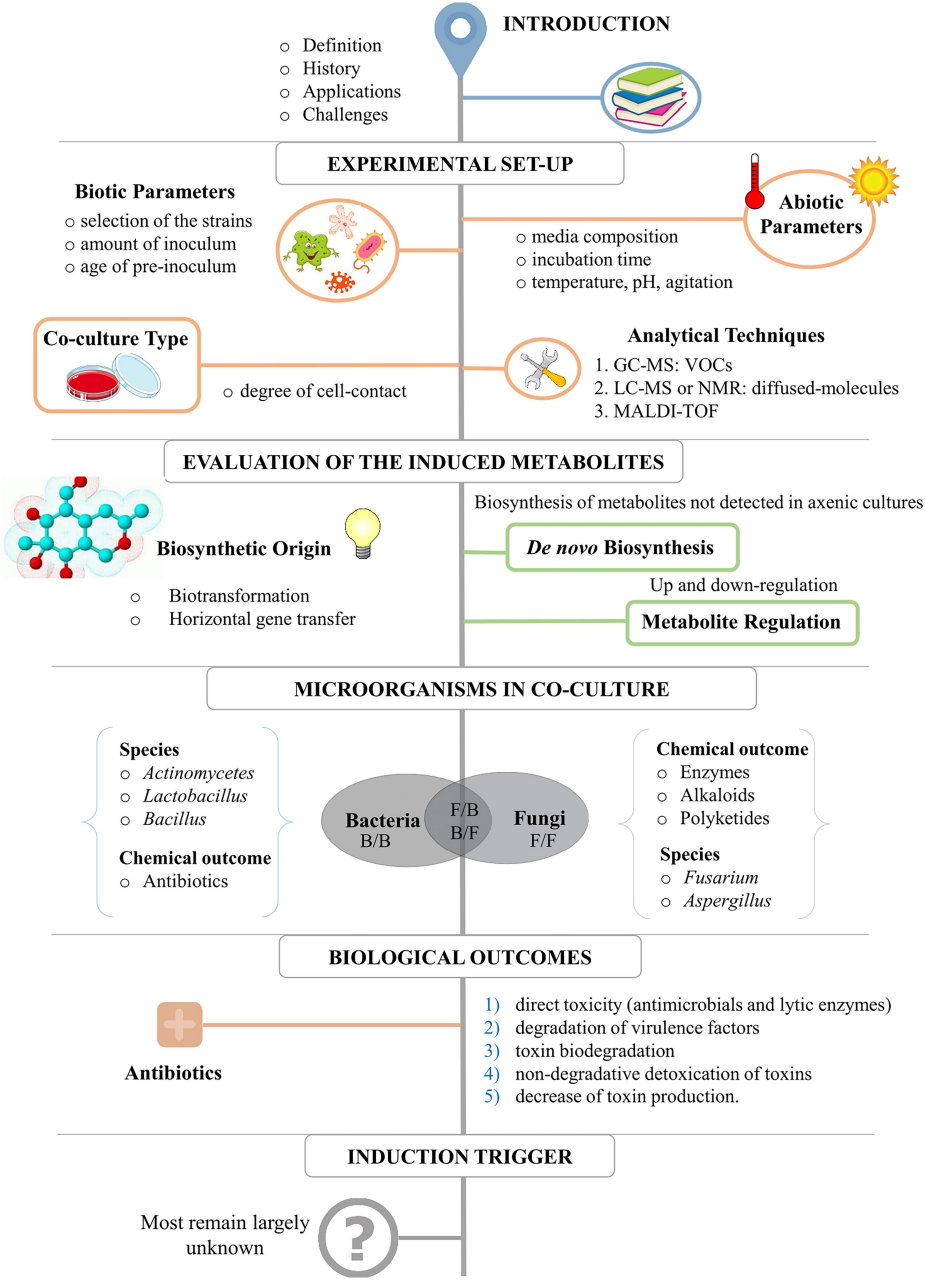
Topics for the application of co-culture for the enhancement of chemical diversity.

Figure 2 represents a schematic workflow of the content of this review. It starts with the basics for the experimental set-up for a successful data interpretation, including the evaluation of critical abiotic and biotic parameters and analytical techniques. Following, we discuss the results of most co-culture studies from 1978 to 2022, describing the most common metabolic outcomes, the microorganisms most used for co-cultivation (and the reason why), the biological and ecological outcomes, and perspectives for the use of this strategy to explore microbial diversity. The criterium for selecting references was the use of only experimental studies published from 1978 to 2022 in impact and peer-reviewed Journals. These studies describe either chemical or biological induction as a direct result of co-cultivation between microorganisms (i.e., fungi or bacteria) and the use of co-culture as a strategy to increase chemodiversity. A Table in which all these studies are summarized can be found in the Supplementary material. The table includes the inducer and challenge strains, type of media, class, and bioactivity of the induced compounds, and the original publication.

Lastly, although many different types of interactions are possible in co-culture experiments, the community still struggles to standardize what the microbes involved in the experiments are called. In this review, microorganisms that produce the specialized metabolites as a consequence of co-cultivation will be referred to as the *inducer strain*. This is because these strains induce their biosynthetic pathways for the production of these compounds. Similarly, the strain that is interacting with the inducer will be named as *challenge strain* and includes all the microbes that provide the biotic interaction for the induction. This latter category also includes the *auxiliary species*, which are challenge microbes that alter media composition by the selective consumption of nutrients, aiding the inducer strain in the production of given metabolites.

## 2. Setting up a co-culture experiment

### 2.1. Biotic and abiotic parameters

It is well known and repeatedly reported in the literature that the choice of growth conditions (i.e., nutrients, temperature, pH) can affect the chemical profile in axenic cultures (Bode et al., 2002; Scherlach and Hertweck, 2009). However, the biotic parameters and the degree of cell–cell communication between strains also need careful consideration to define the best condition for metabolic induction in co-culture (Goers et al., 2014). In the experimental design for co-culturing, we have identified seven factors that should be considered. These are the (1) selection of the co-culture experimental system, which is dependent on the degree of contact between the cell and detailed in the section below (2.2); (2) the selection of the inducer and challenge microbes, based on the desired type of microbe interaction (e.g., antibiosis, symbiosis, mutualistic and others); (3) taxonomic criteria or biological/ecological data of the strains; (4) age of pre-inoculum, i.e., the incubation period of each strain prior to co-cultivation; (5) amount of inoculum from each strain, considering the growth rate of the selected microbes; (6) incubation time; (7) abiotic factors, such as temperature, pH, nitrogen/carbon/phosphate sources, agitation speed, luminosity, type of media (liquid or solid), aeration and water availability. Furthermore, suppose the research targets the evaluation of an ecological response rather than an increase in chemical diversity. In that case, it is also critical to perform a detailed assessment of all biotic and abiotic variables present in the natural biological system to mimic the physiological conditions that elicit the same complex reactions in nature (Hogan, 2006). One good example is the study of mutualistic microbes *Candida albicans* and *Pseudomonas aeruginosa*, commonly as a community in the sputum of cystic fibrosis (*CF*) patients. Studies have shown that only in *CF* isolates, *C. albicans* produces farnesol. This sesquiterpene acts to inhibit the filamentation of its producer, as well as improve swarming motility and the release of secondary metabolites in *P. aeruginosa*, demonstrating how the microbial interactions contribute to disease in polymicrobial infections (Cugini et al., 2007; McAlester et al., 2008).

The optimization of the factors that influence a co-culture experiment is usually performed by individual assessment of the variables or by multifactor and systematic analysis in a Design of Experiment (DoE). Any subtle change may dramatically affect the production of individual metabolites, i.e., each parameter could regulate the activation of specific BGC. For example, Slattery and colleagues have demonstrated that 24-h pre-inoculation of the inducer bacteria *Streptomyces tenjimariensis* was essential to enhance the production of the aminoglycoside antibiotics, istamycins A–B, when in co-culture with challenge marine bacteria. Simultaneous co-inoculation or pre-establishment of challenge-microbes without pre-inoculation resulted in a significant decrease in the production of istamycins compared with the monoculture of *S. tenjimariensis* (Slattery et al., 2001). Similarly, studies with *Lactococcus lactis* have shown an increase in the production of the polycyclic peptide nisin only in co-culture with specific bacteria that consume lactic acid as a carbon source. These challenge species include *Yarrowia lipolytica* (Ariana and Hamedi, 2017), *Saccharomyces cerevisiae* (Liu et al., 2006), and *Kluyveromyces marxianus* (Shimizu et al., 1999), and have provided a booming increase of over 85% in nisin production in comparison to the control.

More recently, researchers have also created novel methodologies for the monitoring of species dynamics and microbial growth in liquid cultures. Some successful strategies include the use of high-throughput online monitoring and the use of strains tagged with fluorescence. On the one hand, studies showed that real-time measurements of the respiration rate allowed the online tracking of sugar formation in noncellulolytic bacterium and cellulolytic fungi (Finger et al., 2022). On the other hand, strains tagged with fluorescence proteins and computer-controlled optogenetic modulation of bacterial growth helped dissect the individual strain contributions, providing knowledge of complex cocultures and accelerating the setup of other tailor-made coculture in bioprocesses (Finger et al., 2022; Gutiérrez Mena et al., 2022).

### 2.2. Types of co-cultures experiments

The degree of contact between microbes defines the type of experimental setting for co-cultivation and is a critical step in the experimental workflow. It should be based on the study’s purpose and the analytical techniques available to evaluate the chemical profiles. For example, suppose the work targets the measurement of volatiles by gas chromatography. In that case, it is crucial to use volatile trapping and select an experimental set-up that prevents direct cell-to-cell contact. Moreover, if the work targets the identification of unknown molecules, methods that allow the discovery of unknowns and that enable scaling-up for isolation/purification steps also need consideration.

**Figure 3:**
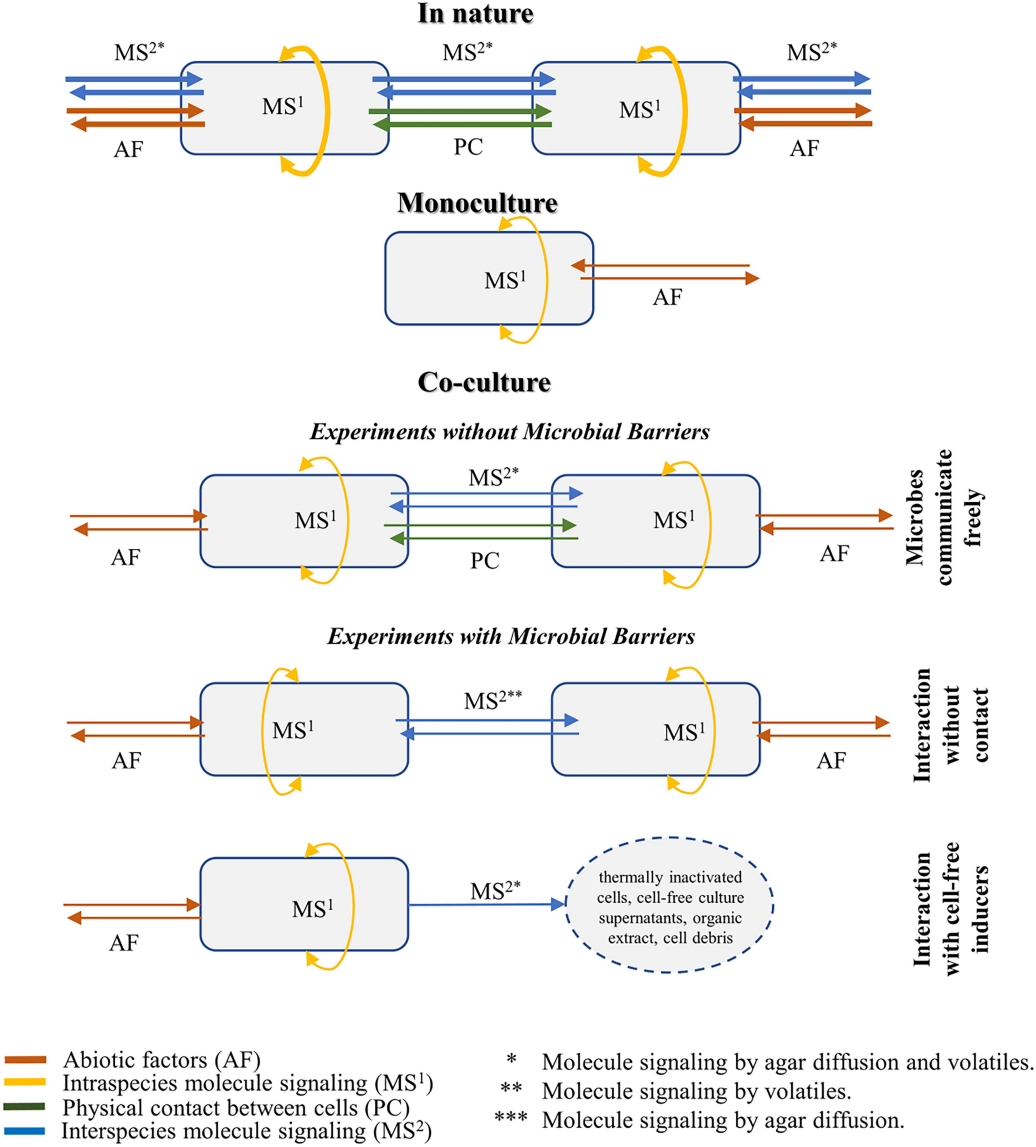
Illustration of the microbial interactions and molecule signaling in the natural environment, monoculture, and co-culture settings. Co-cultivation methodologies are divided into three different experimental conditions, considering the degree of contact between the cells and the type of induction evaluated in the study.

Figure 3 shows the two most common set-ups for co-culture evaluation, considering the degree of contact between the cells and the type of induction evaluated in the study. These methodologies are divided into three different experimental conditions, and their applications, advantages, and disadvantages are described in the sections below.

#### 2.2.1. Experiments without microbial barriers (direct cell–cell contact)

In most co-culture systems, the activation of cryptic genes is achieved by the inoculation of the inducer and target microbes without any physical barriers between them. In these systems, metabolic induction is the most comprehensive given that it could happen either by signaling molecules that are secreted into the media or by the diffusion of volatiles (Figure 3). Both secretion and diffusion contain specialized compounds, detailed in this review, and autoregulatory metabolites, briefly discussed in Box 1. In practice, this high degree of contact means that both strains affect each other simultaneously, leading to a complex system that elicits nature’s most similar response (Bader et al., 2010).

For fungal cultures, solid media is the standard method to unbiasedly screen bioactive metabolites because the colony growth, the type of interaction (based on changes in microbial morphology), and the individual contribution of each microbe for the metabolic induction can be visually assessed (Bertrand et al., 2013a; Yao et al., 2016). For instance, growth inhibition from antagonistic interactions is readily visible during the first days of inoculation, facilitating the target analysis of antimicrobials compounds (Moree et al., 2013; Akone et al., 2016; Yao et al., 2016; Serrano et al., 2017).

**Figure 4:**
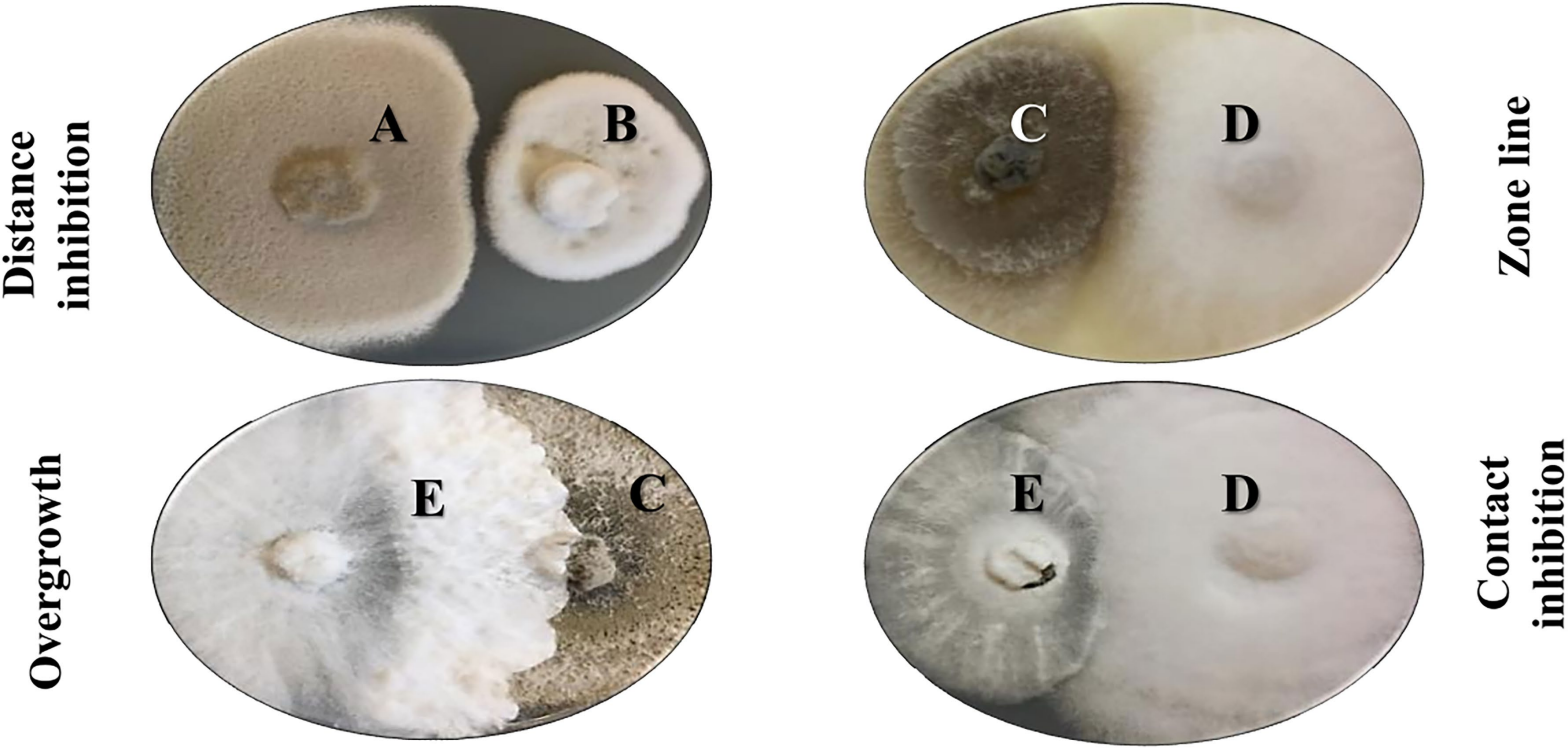
Morphological interactions in co-culture experiments with five different fungi on solid media. A: *Alternaria alternata*, B: *Colletotrichum acutatum*, C: *Diaporthe eres*, D: *Fusarium oxysporum*, E: *Xylaria cubensis*. Bertrand and colleagues reported four major morphological outcomes in these co-culture experiments in solid media: distance inhibition, zone lines, contact inhibition, and overgrowth (Bertrand et al., 2014b).

In 2014, Bertrand and co-workers reported that fungal co-culture in solid media could result in four major interaction types. Indeed, our morphological evaluation of *Alternaria alternata*, *Colletotrichum acutatum*, *Diaporthe eres*, *Fusarium oxysporum*, and *Xylaria cubensis* has shown a very dynamic interaction between strains, with one strain exhibiting all four morphological responses according to the challenge microbe (Figure 4). The first type of morphological change is named *distance-inhibition* and is usually a result of antibiosis. It happens when fungal growth stops at a distance from the competing culture, possibly due to the release of antimicrobial secondary metabolites in the media. The second type is denominated as *zone lines* and can also be the result of the release of antimicrobial metabolites. The fungal colonies grow until contact and form a dark precipitate in the confrontation zone. In the third group, both fungi grow until contact but do not produce any evidence of metabolite release or confrontation, respecting each other place in a *contact-inhibition* response. Lastly, the fourth type of interaction occurs when fungi grow until contact with a partial or complete invasion of one fungus by the other, in a so-called *overgro*wth. Although there is no clear evidence of antagonist release of specialized metabolites in this setting, this competitive interaction is a clear result of morphological and developmental changes by microbial signaling (Bertrand et al., 2013a, 2014b).

Up until now, there is a pronounced lack of information on the exact type of colony morphology and its corresponding biological outcome in solid media experiments. Most co-culture reports only indicate the chemical induction without any standardized description of morphological interactions or growth conditions. Moreover, in most studies that use this experimental set-up, the metabolome is only assessed in terms of secreted molecules, failing to mediate volatile organic compounds (VOC), despite their ecological and biological importance (König et al., 2006; Nonaka et al., 2011; Ola et al., 2013).

Good examples of morphological descriptions include Glauser et al. (2009) and Bertrand et al. (2013b). Glauser and co-workers evaluated the *zone line* of two grapevine fungal pathogens *Eutypa lata* and *Botryosphaeria obtuse*. From the confrontation zone of these two wood-decaying fungi, they were able to identify the antifungal polyketides *O*-methylmellein, 4-hydroxy-8-*O*-methylmellein, and 5-hydroxy-8-*O*-methylmellein (Glauser et al., 2009). Similarly, Bertrand and co-workers isolated the polyketide 4″-hydroxysulfoxy-2,2″-dimethylthielavin in the *distance-inhibition* zone between *Trichophyton rubrum* and *Bionectria ochroleuca*. This compound was found in the pure strain culture of *B. ochroleuca* and was up-regulated in the confrontational region between these fungi in solid culture (Bertrand et al., 2013b).

In filamentous bacteria and fungi, mycelial morphology also plays a crucial role in the metabolome and can lead to the production of different specialized metabolites. This dependence happens because variations in morphology interfere with the degree of nutrients, the oxygen transfer within the pellets, and the rheological properties of the fermentation broth. All these parameters affect nutrient distribution within the fermentation, causing activation of different BCGs and, hence, induction of different metabolites (Fang et al., 2000; Wang et al., 2017). For example, the linkage between mycelial morphology in *Streptomyces hygroscopicus* and the production of the antibiotic rapamycin has been exemplified by Fang et al. (2000). In this study, mycelial morphology was influenced by the presence of the antibiotic thiostrepton at sub-inhibitory concentrations, leading to the formation of a dispersed mycelial morphology that is preferred for antibiotic production (Fang et al., 2000). Similarly, erythromycin production has also shown to be regulated by the pellet size (Wardell et al., 2002), enhancing its production in increased hyphal strength. To date, several genetic determinants involved in mycelial morphology have been identified in *Streptomyces*. Some highly recommended studies on the topic can be found in Xu et al. (2008), Koebsch et al. (2009), and van Dissel et al. (2014, 2015).

**BOX 1. Induction by autoregulatory molecule.**

Chemical and morphological changes can also be a result of metabolites that were known to act only as intraspecies signaling (Crespi, 2001; Bader et al., 2010). In the early days, the release of these small molecules, named diffusible signal factor (DSF) or autoregulatory molecules, was focused on the intraspecies response in opportunistic pathogens. However, there is increasing evidence that these molecules can promote regulation in other microbes, highlighting their importance in polymicrobial diseases (McAlester et al., 2008).

Recent research shows that DSF modulates intra- and inter-species by various mechanisms, inflecting antagonistic to mutualistic responses, morphological transitions, virulence control, and release of important secondary metabolites. For example, *Xanthomonas campestris* and *Stenotrophomonas maltophilia* produce the DSF fatty acid *cis*-11-methyl-2-dodecenoic acid to control intraspecies biofilm formation and virulence capacities of its producers (Dow et al., 2003; Torres et al., 2007; He and Zhang, 2008). However, a different study has shown that, in co-culture, this molecule can also promote hyphae inhibition in *Candida albincans* (Wang et al., 2004) and influence biofilm architecture, stress response, and polymyxins tolerance in *Pseudomonas aeruginosa* (Ryan et al., 2008). A similar answer has also been found in the DSF α,β-unsaturated fatty acid cis-2-dodecenoic acid (BDSF) produced by *Burkholderia cenocepacia*, restoring biofilm and extracellular polysaccharide production in *X. campestris* and inhibiting germ tube formation of *C. albicans* (Boon et al., 2008).

Regulators responsive to autoregulatory molecules in *Streptomyces* are also well known. Feedback control by biosynthetic intermediates has been demonstrated for actinorhodin, jadomycin, and simocyclinone biosynthesis (Van Der Heul et al., 2018). Furthermore, γ-butyrolactones have also been shown to regulate antibiotic production and morphological differentiation, binding to receptors involved in the regulation of specific antibiotic BGC (Takano, 2006; Willey and Gaskell, 2011; Van Der Heul et al., 2018).

#### 2.2.2. Experiments with microbial barriers (without cell-cell contact)

While some microbial interactions in co-culture experiments depend on cell-to-cell contact, in other conditions, induced specialized metabolites are released even without clear evidence of contact. In that case, metabolic variation can be evaluated either by (a) co-cultivation with a reduced degree of contact between the microbes, (b) by using inactivated microbes (Fourati-Ben Fguira et al., 2008; Liang et al., 2019), or (c) by the addition of elicitors, found as pure compounds, extracts, cell debris, or cell-free supernatants (König et al., 2013).

In the case of living cells, the contact between the strains can be controlled by establishing barriers that prevent physical contact between cells in the culture medium. This is usually achieved by using culture membranes or segregated Petri dishes (Bogdanowicz and Lu, 2013) and it is the ideal setting to determine if physical contact is mandatory for the metabolic induction, as well as to check the role volatile compounds might play in the interaction. Orban and co-workers have recently shown how the use of bi-plates enabled the study of bacterial volatiles on the mycelial growth of mushrooms, identifying the VOC 2,5-diisopropylpyrazine as the main promotor of fungal growth when the bacteria *Paenibacillus peoriae* strain M48F was co-culture with *Pleurotus* species (Orban et al., 2023).

The use of pure compounds or a simple mixture of compounds to induce cryptic BGC is known as small molecule elicitor screening and has gained some attention in the last 5 years for the high throughput screening of cryptic compounds. Some excellent references can be found in the literature (Seyedsayamdost, 2014; Okada and Seyedsayamdost, 2017; Xu et al., 2017; Mao et al., 2018; Xu et al., 2019; Moon et al., 2019b; Zhang and Seyedsayamdost, 2020). In this approach, the main concern is to select an adequate concentration of the metabolite (s) added to the cultures. In theory, the ideal concentration can enable metabolic regulation without killing the inducer strain, known as subinhibitory concentration. Several assays can be done for this assessment, in which minimum inhibitory concentration (MIC) and agar-disc diffusion assay (DDA) are the most used. In both, different concentrations of elicitors can be tested on the same Petri dish to determine at which molarity there are no inhibition growth zones (Bauer et al., 1966). Some practical approaches to *in vivo* antimicrobial bioassays can also be found (Balouiri et al., 2016). Lastly, it is important to keep in mind that the tested compounds need to be solubilized in solvents that do not interfere with microbial metabolic production, to avoid false positives when determining subinhibitory concentration. Specifically, dimethylsulfoxide (DMSO) has a proven effect on bacterial growth and should not be used in concentrations higher than 2% (Basch and Gadebusch, 1968).

Benitez and colleagues demonstrated that *Bacillus amyloliquefaciens* LBM 5006 produces a higher concentration of an antibiotic peptide when in liquid co-culture with *Escherichia coli* ATCC 25922. Interestingly, the induction of bacteriocin production was also achieved with thermally inactivated *E. coli* cells and cell debris after cellular fractionation (Benitez et al., 2011; Chanos and Mygind, 2016). Contrarily, Cueto and co-workers have shown that the antibiotic pestalone was induced only by cell-to-cell contact. Only when in co-culture with the antibiotic-resistant marine α-proteobacterium, *Pestalotia* sp. produced the antibiotic. Neither organic extracts nor cell-free culture supernatants of the chlorinated benzophenones were detected (Cueto et al., 2001).

### 2.3. Analytical techniques

In most co-culture experiments, metabolite extraction is performed using conventional protocols that use an organic solvent. Moreover, if the microbes are filamentous, i.e., most plant-associated fungi or Actinomycetes, an extra step of cell-rupture with acetone or ethyl acetate may be required, enabling analysis of secreted and intracellular compounds (Bertrand et al., 2013a; Ross et al., 2014; Serrano et al., 2017). The most common solvents used for liquid media cultures are ethyl acetate (Cueto et al., 2001; Ross et al., 2014; Selegato et al., 2019) or methanol (Zhu et al., 2011), whereas, for solid media, authors preferred the use of acetone (Serrano et al., 2017) or the mixture of dichloromethane/methanol/water (v/v/v 64:36:8) (Glauser et al., 2009; Bertrand et al., 2013a). For microbiome communities, authors also reported the use of methanol/acetonitrile followed by incubation at −20°C and centrifugation to break cells of bacteria as well as induce the precipitation of proteins (Zimmermann et al., 2019).

Although successful in the profiling of microbial metabolome, the conventional protocols continuously face problems of reproducibility, probably because scientists do not control several essential abiotic parameters of microbial growth, such as aeration, luminosity, and inoculum age. Other challenges faced in chemical evaluation are intense signals from the culture medium components and low metabolite yield. Methods that improve speed and sensitivity and the standardized use of controls (blank and culture media blanks) are urged but scarce in the literature. Bertrand and co-workers described one successful example of methodological developments. In their approach, rapid and simultaneous assessment of the metabolites was performed by down-scaled co-culture using multi-well plates, strongly stimulating fungal growth. Moreover, the use of a 12 well-plate enables the generation of a large number of replicates to overcome the lack of reproducibility and had a reliable up-scale using a 15 cm-petri dish for metabolite purification and isolation. These results partially overcame the difficulties of standard 9 cm plates regarding the growth rate of different fungi, metabolic changes based on the duration of growth, and lack of sufficient material for isolation, providing a rational way to highlight the metabolic induction (Bertrand et al., 2014a).

A comprehensive assessment of co-culture induction should include measurements of both diffused metabolites and VOCs to ensure an unbiased analysis of the microbial interaction. However, only recently, efforts have been made to develop methodologies that provide this holistic analysis of molecular dynamics. Azzollini and co-workers described a strategy for the concomitant study of volatile and non-volatile metabolomes in a co-culture system. In this methodology, the fungi were directly grown in vials which are first submitted to a headspace solid-phase microextraction and gas-chromatography mass-spectrometry (HS-SPME-GC–MS) analysis, to profile VOCs, and then extracted with organic solvents, for analysis by liquid chromatography and high-resolution mass spectrometry (LC-HRMS) (Azzollini et al., 2018).

Another challenge of co-culture experiments is the absence of high-throughput methodologies. To our knowledge, only Bertrand et al. (2013a,b) have described a method for the high throughput screening of co-culture of filamentous fungi grown in solid media (Bertrand et al., 2013a). The development of protocols that reliably screens microbial strains would represent a significant advance in drug discovery. Moreover, successful high throughput cases have already been reported for monoculture in the presence of small molecules (Seyedsayamdost, 2014; Moon et al., 2019b; Zhang and Seyedsayamdost, 2020; Zhang et al., 2022) and could be extrapolated for the study of microbial interactions. These methodologies have already facilitated the bio-guided selection of promising strains, as well as the chemical evaluation of BCG activation under different experimental conditions.

The chemical interpretation of secreted molecules in co-cultures is usually carried out by mass spectrometry. Successful results were achieved using LC–MS (Bertrand et al., 2014a; Hoshino et al., 2015b) and MS-imaging methodologies (Moree et al., 2012, 2013). However, due to the complexity of the isolated compounds and their unknown nature, proper identification of the induced metabolites often requires the use of complementary spectroscopic tools, such as MS/MS, 1D and 2D Nuclear Magnetic Resonance spectroscopy (Angell et al., 2006; Wang et al., 2013; Gerke et al., 2022). In the case of VOCs, the profiles are usually obtained by headspace trapping using static or dynamic techniques, and chemical analysis is conducted by gas chromatography–mass spectrometry analysis (Brasch et al., 2014; Orban et al., 2023). Recently, Brasch and co-workers showed by enantioselective GC–MS that the isolated compounds were stereochemically pure (Brasch et al., 2014). In this study, these induced metabolites were trapped using solid-phase-micro-extraction (SPME) that were inserted into the dishes through a small hole and left in place for 2 h to allow VOC adsorption. Some practical approaches to volatile analysis using solid phase microextraction (SPME)-GC–MS were reported by Tholl and co-workers (Tholl et al., 2006).

MALDI-TOF imaging (IMS) has been gaining particular attention in the study of microbes in solid media, mainly due to its ability to create overlays of the diffusion patterns of specialized metabolites in agar, resulting in visual images of the metabolome in the microbial colonies (Moree et al., 2012; Traxler et al., 2013; Moree et al., 2014; Clark et al., 2018). The interest in this technique came from its already established protocols in the study of small molecule communication between tissues and cells in clinical data which enables the development of models for the study of cell communication in diseases or the efficacy and safety testing of drugs and toxicants (Zink et al., 2019; Spencer et al., 2020). The advantage of MALDI Imaging for microbiology is the fact that several metabolites can be simultaneously visualized according to their spatial distribution, enabling the comparison of chemical signatures and the correlation with biological phenotypes of interest (Stasulli and Shank, 2016; Müller et al., 2022). In co-culture, IMS enables the observation of previously undetected coculture-metabolites and the microbial strain that produced these compounds (Watrous and Dorrestein, 2011; Bleich et al., 2015; Chen et al., 2018; Teixeira et al., 2022). Moreover, the concepts of molecular cluster families integrated into the ideas of MS/MS networking allowed the annotation of unknown *m/z* peaks, facilitating the dereplication of analogs produced by the same BGCs (Moree et al., 2012; Watrous et al., 2012; Moree et al., 2013; Stasulli and Shank, 2016; Martin et al., 2019; Wang et al., 2022). More and colleagues used IMS and MS Molecular Networking to identify the molecules produced by *Bacillus amyloliquefaciens* when interacting side-by-side with two fungal strains, *Aspergillus fumigatus*, and *A. niger*. The visualization was performed directly on the agar without the need for extraction. It led to the identification of several lipopeptides iturin analogs as the predominant antifungal factors associated with this *Bacillus* strain (Moree et al., 2013).

## 3. Types of metabolic induction in mixed cultures

Recent data have suggested that metabolic induction can happen either by activating BCGs from only one strain or by a combined effort between the competing species. Usually, single culture activation is genetically more straightforward and leads to the regulation (up or down-regulated) of specialized metabolites and/or the production of analogs from the same gene cluster (Zhu and Lin, 2006). Multispecies activation, on the other hand, is much more complex and provides metabolite enhancement by multistep strategies, such as cross-biotransformation (Hoefler et al., 2012; Moree et al., 2012) or horizontal gene transfer (Kurosawa et al., 2008).

An excellent example of biotransformation in co-culture was provided by Moree and co-workers, that studied the production and regulation of phenazines in the bacteria *P. aeruginosa*. Phenazines play a vital role in bacteria defense, acting on electron shuttling, biofilm development, and the production of toxic superoxides. When in co-culture with the fungus *A. fumigatus, P. aeruginosa* is known to produce higher amounts of these secondary metabolites. Using MALDI-TOF imaging mass spectrometry (IMS) combined with MS networking, Moree showed that, even though there was an up-regulation of phenazine compounds on the confrontation zone, *A. fumigatus* was able to rapidly convert these compounds into other chemical derivatives with lower antifungal activity, enhanced bacterial toxicity and the ability to induce the production of fungal siderophores (Moree et al., 2012). The same context of biotransformation was also recently reported between *Alternaria* and *Trichoderma*, in which the first produces alternariol in co-culture, while the second rapidly metabolizes it into its hydroxylated form (Tian et al., 2023).

Kurosawa and co-workers also revealed an unusual co-culture induction, in which actinomycin genes from the highly stable antibiotic-producer *Streptomyces padanus* were transferred to the multi-antibiotic resistant mutant of *Rhodococcus fascians*, a bacteria species that is not known for any antibiotic production. In a liquid co-culture experiment, they demonstrated that a horizontal gene transfer from *S. padanus* to *R. fascians* led to the production of two aminoglycosides antibiotics by *Rhodococcus*. These compounds, named rhodostreptomycin A and B, strongly act against *S. padanus* with complete elimination of the competing bacteria. Genomic analysis revealed that, in mixed cultures, *Rhodococcus* harbors a large DNA segment of the *Streptomyces* strain, illustrating the underreported microbial capability to produce new antibiotics through horizontal gene transfer (Kurosawa et al., 2008).

**Figure 5:**
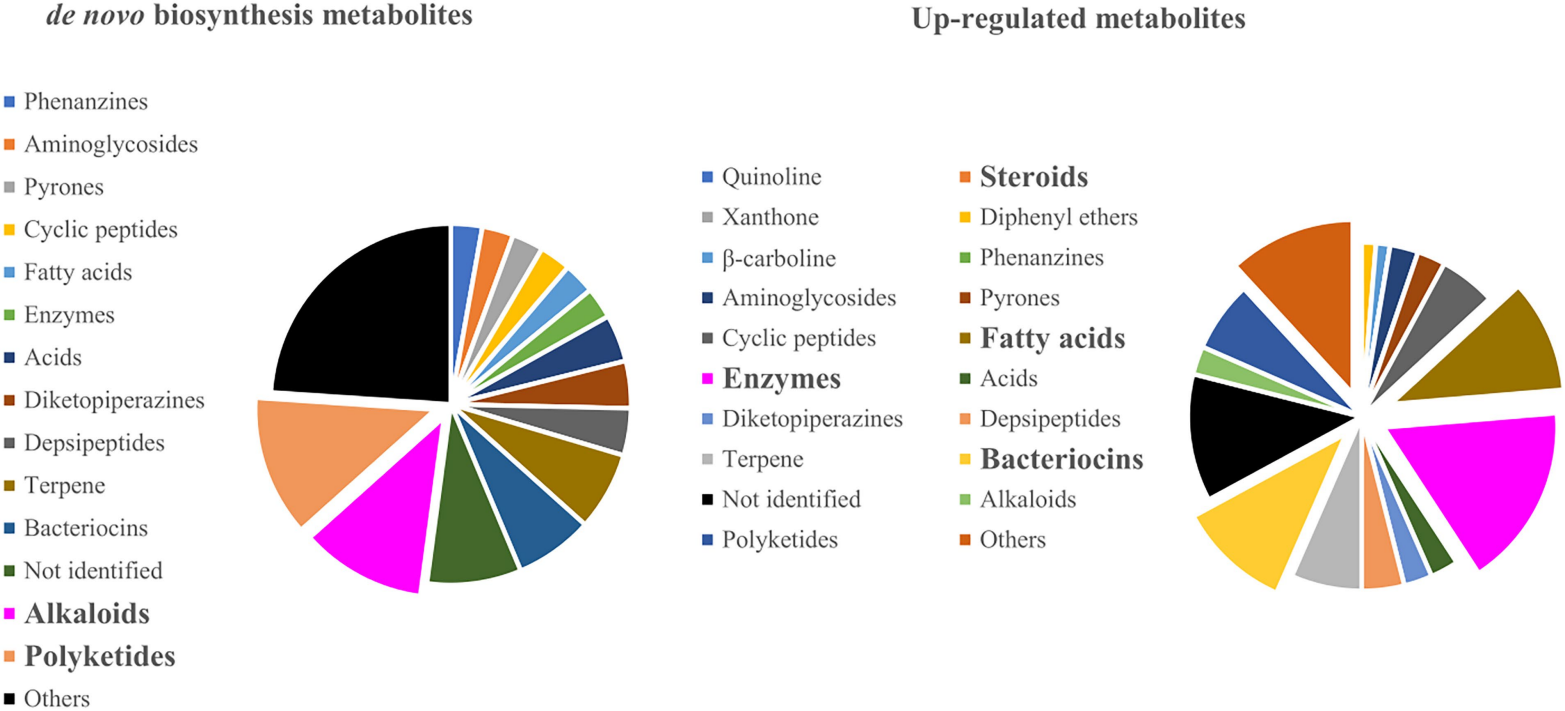
Metabolite classes that were produced on *de novo* biosynthesis and up-regulated co-culture experiments.

Most experiments that report up-regulated metabolites are focused on the evaluation of known antibiotics (e.g., bacteriocins, aminoglycosides, quinolines, and others) and enzymes involved in ecological and industrial processes. These studies target mainly to improve biotechnological and pharmaceutical applications (Figure 5). However, for *de novo* biosynthesis, the most common metabolic classes are alkaloids (Zhu and Lin, 2006; Park et al., 2009; Zuck et al., 2011; Zhu et al., 2013) and polyketides (Watanabe et al., 1982; Schroeckh et al., 2009; Onaka et al., 2011; Chagas et al., 2013; Ross et al., 2014), indicating that fungi and bacteria usually induce these classes as a defense strategy during biotic stress (Figure 5). For example, Chagas, Dias, and Pupo showed that the interactions between the endophytic fungi *Alternaria tenuissima* and *Nigrospora sphaerica* have significantly increased the production of polyketides alterperylenol and stemphyperylenol, the latter displaying antifungal and cytotoxic effects against *N. sphaerica*. Moreover, these compounds were shown to act against the endophytic fungi but displayed no phytotoxicity to the host plant *Smallanthus sonchifolius* even at high concentrations. This indicates a selective biological activity and meaningful ecological interaction between endophyte-endophyte and endophyte-host plants (Chagas et al., 2013). Similarly, Zhu and co-workers demonstrated the production of a new alkaloid, together with neoaspergillic acid and ergosterol, during the co-culture of two marine-derived mangroves epiphytic *Aspergillus* sp. Both the alkaloid aspergicin and neoaspergillic acid showed significant antibacterial activity against the Gram-positive bacteria *Staphylococcus aureus*, *S. epidermidis*, and *Bacillus subtilis*, as well as against three Gram-negative bacteria *B. dysenteriae*, *B. proteus*, and *E. coli* (Zhu et al., 2011). An excellent review of the structural diversity in co-culture based on a similarity network can be found (Arora et al., 2020).

One important aspect of microbial co-culture is to correctly find which microorganism is responsible for the production of the increased specialized metabolite. Most reports identify the inducer microorganisms only by comparison with the axenic cultures or by evaluating confrontation zone and isolated mycelial parts. However, these comparisons do not consider the possibility of gene transfer or the fact that both strains could produce the same metabolic classes. Confirmation is required by not only a detailed chemical evaluation but also high-quality genome sequencing studies (Bogdanowicz and Lu, 2013). Several strategies to confirm the metabolic pathways and enzymes that encode the induced molecules are available in the literature and usually combine gene clustering with computational tools (Medema et al., 2015). Here we highlight NP.searcher, ClustScan (Li et al., 2009), CLUSEAN (Weber et al., 2009), antiSMASH (Weber et al., 2015), SMURF (Khaldi et al., 2010), MIDDAS-M (Umemura et al., 2013), ClusterFinder (Cimermancic et al., 2014), CASSIS/SMIPS (Wolf et al., 2016), NRPSpredictor (Röttig et al., 2011), SBSPKS (Anand et al., 2010) and C-Hunter (Yi et al., 2007). These tools have helped in predicting the structure of specialized metabolites and over the year, have evolved from cluster cores of Nonribosomal Peptide Synthetase (NRPS) and Polyketide Synthase (PKS) to detailed information over many signature enzymes. However, these methods should be handled carefully because mismatches can occur in the prediction of the chemical structures of the clusters. Detailed reviews of each strategy are available in the further mentioned references (Chavali and Rhee, 2018; Singh et al., 2021; Almeida et al., 2022).

## 4. Microorganisms in mixed cultures

**Figure 6:**
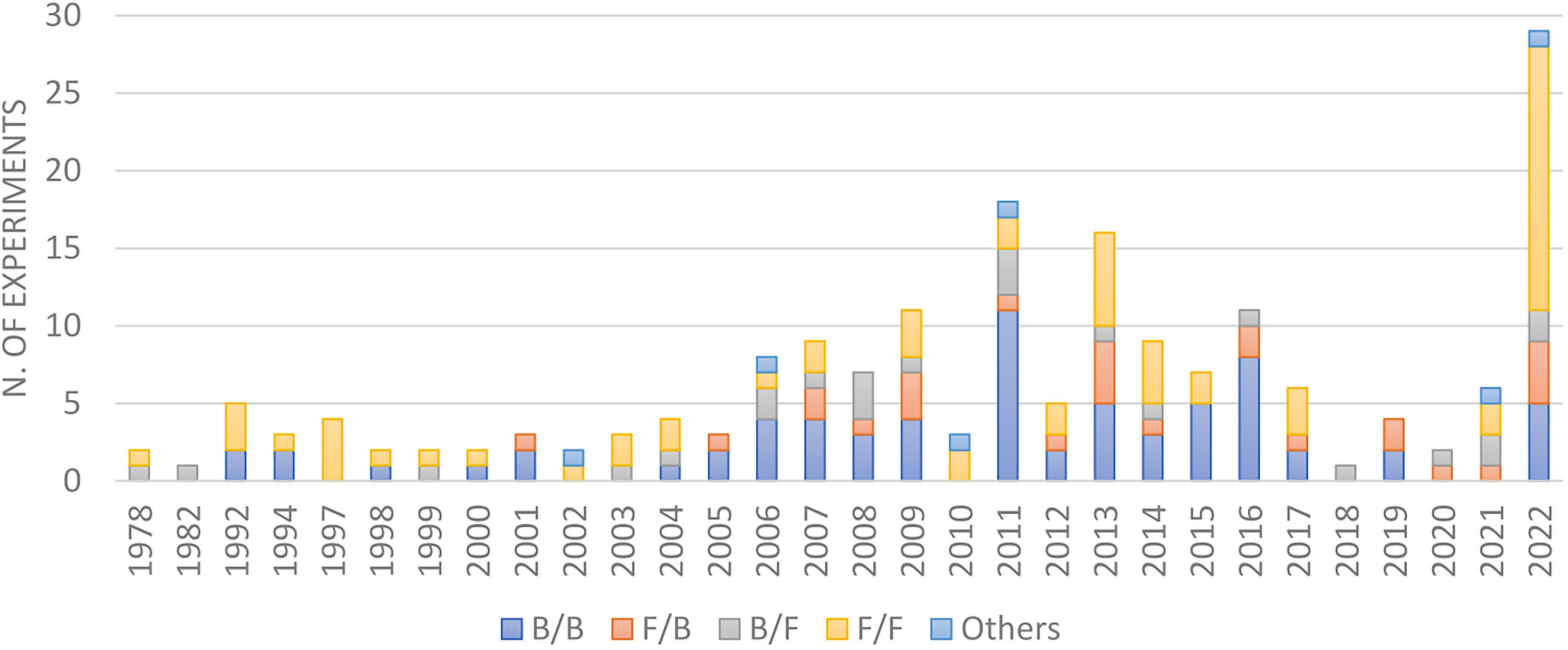
Microorganisms pairwise used in co-culture experiments since 1978.

In the early days, most co-cultures were performed using two fungi or two bacteria (Figure 6), majorly due to the difficulties in growth rate and reproducibility among eukaryotes and prokaryotes. However, owing to the fact that microorganisms co-exist in nature in close associations with each other, alongside the recent development in media culture and microbiology techniques, the interactions of microbes from different phylogenetic orders have been increasingly evaluated, including the study of fungi/bacteria, bacteria/fungi, oomycete/oomycete (Ojika et al., 2011) or the incubation of more than two microbes (Pettit et al., 2010).

Among the untraditional co-cultures, the most prominent are bacterial/fungi, in which the bacterium is the inducer strain, and fungi/bacteria, in which the fungus was reported to produce the induced metabolites. However, studies of microbial co-culture have achieved the use of more than two microorganisms in one culture system. Indeed, Chevrette and co-workers have discovered that the abundance of transcripts of BGCs and the metabolic profiles differ between monocultures, dual-cultivation, and a tripartite community and that the dynamics of specialized metabolism depend on the interspecies interactions and the community species composition (Chevrette et al., 2022). To exemplify these dynamics, Pettit and co-workers have simultaneously co-cultured five fungi (*Ovadendron sulphureoochraceum*, *Ascochyta pisi*, *Emericellopsis minima*, *Cylindrocarpon destructans*, and *Fusarium oxysporum*) for the biosynthesis of potential antineoplastic substances. This simultaneous cultivation resulted in a *de novo* production of lateritin, a *N*-methylated depsipeptide that inhibits the growth of a mini-panel of human cancer cell lines and shows antibacterial activity against Gram-positive bacteria (*Micrococcus luteus*, *S. aureus*, *E. faecalis*, and *S. pneumoniae*) and antifungal potential against *C. albicans* (Pettit et al., 2010). The five fungi did not synthesize detectable levels of lateritin individually. Although it is not clear which microbe is responsible for the induction, it validates the growing body of evidence that co-culture is a viable strategy for natural product drug discovery.

### 4.1. Bacterial co-culture: *Actinomyces*, *Lactobacillus*, and *Bacillus*

**Figure 7:**
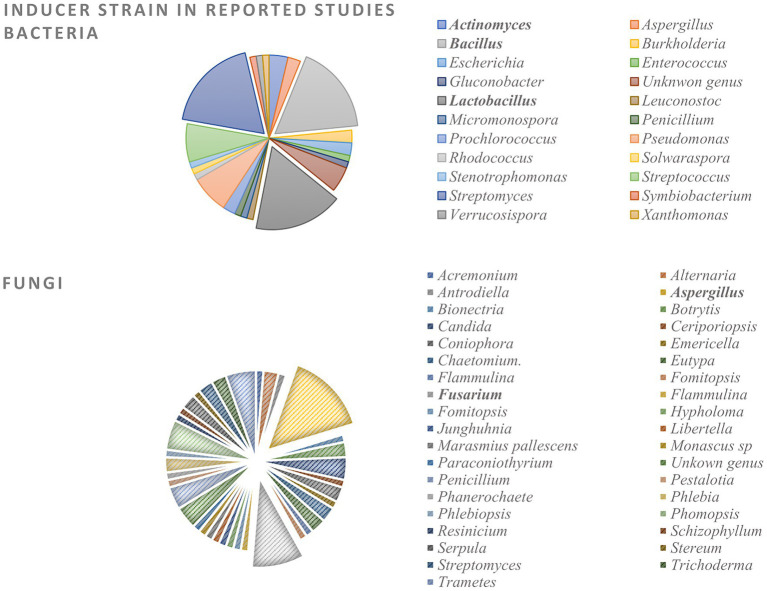
Bacteria and fungi inducer strains in reported co-culture studies from 1978 to 2022.

Evaluation of the reported bacterial co-culture experiments from 1978 to 2022 revealed that the majority of inducer strains were Gram-positive organisms from the genera *Streptomyces*, *Bacillus*, and *Lactobacillus* (Figure 7). These species will be individually discussed below, detailing their co-culture outcomes and the reasons for their success in this experimental condition.

#### 4.1.1. Actinomycetes

For many years, Actinomycetes have received special attention as “*the richest reported microbial source of bioactive secondary metabolites*.” This title comes from the reported capacity of the family to activate their numerous BCGs according to the environmental conditions, producing several polyketides, phenazines, peptides, isoprenoids, indolocarbazoles, sterols, and other specialized metabolites (Nett et al., 2009). Indeed, sequencing of several *Streptomyces* species revealed that their coding capacity is much larger than necessary for basic functioning (Galagan et al., 2005), with more than 20 BCGs exclusively dedicated to the production of secondary molecules (Knight et al., 2003).

Other than its genomic capacity to produce previously unknown molecules, *Streptomyces* species are also the producer of important antibiotics, antifungals, anthelmintics, and antitumor compounds of clinical use (Omura et al., 2001; Traxler et al., 2013; Valliappan et al., 2014; Sung et al., 2017). These commercially available metabolites increase the interest in methodologies that might enhance their yielding, such as co-cultivation, contributing to the constant selection of *Streptomyces* species as inducer strains. The most important antimicrobials enhanced by co-culture are the antituberculosis/antibiotic streptomycin, produced by *Streptomyces griseus*, the immune suppressant tacrolimus used for reducing transplant rejection, found in *S. tsukubaensis*, and the pesticide and antifungal avermectin, a macrocyclic lactone derivative from *S. avermitilis* (Moody, 2014). All these compounds have been enhanced by different fungi and bacteria, showing the pleiotropic effects where one single gene is affected by multiple systems.

In the Actinomycetes family, *Streptomyces* is the most studied genus. Mixed cultivation studies of this species are often performed with pathogens or mycolic-acid-containing bacteria and target the production of both new and up-regulated antibiotics. Bacteria containing mycolic acids in their outer membrane are a prime example in the induction of secondary metabolism in *Streptomyces* strains and have been extensively used for the induction of promising compounds (Onaka et al., 2011, 2015). The metabolite classes identified by co-cultivation include aminoglycosides (Turpin et al., 1992; Slattery et al., 2001; Carlson et al., 2015), diterpene (Cho and Kim, 2012), indolocarbazole alkaloids (Shah et al., 2008; Hoshino et al., 2015c), hydroxamic acid (Traxler et al., 2013), polyketides (Bignell et al., 2010; Onaka et al., 2011), pigments (Onaka et al., 2011), polyenes (Yakovleva and Bulgakova, 1978), butanolide chojalactones (Hoshino et al., 2015b), macrolactams (Hoshino et al., 2015a), enzymes (Hoefler et al., 2012), peptides (Loria et al., 2008; Sung et al., 2017; Shin et al., 2018), and naphthoquinone (Sung et al., 2017), demonstrating the great and unexplored potential of the biosynthetic pathways of these Gram-positive bacterias. It is also worth mentioning that the trigger factors of mycolic-acid-containing bacteria have been attributed not only to molecule signaling but also to concomitant cell-to-cell contact. In several different studies, these challenge strains have enhanced the chemical diversity of fungi and bacteria (Adnani et al., 2015; Hoshino et al., 2015a,b,c) and provided novel secondary metabolites from different biosynthetic pathways such as novel antibiotic polyketide (Onaka et al., 2011), cytotoxic indolocarbazole alkaloid, butanolide chojalactones, and tricyclic macrolactams (Hoshino et al., 2015a,b,c).

#### 4.1.2. Lactobacillus

*Lactobacillus* is a genus of Gram-positive bacteria that are known to produce lactic acid as well as some important antimicrobial compounds. These antibiotics, generally called bacteriocins, are biologically active proteins commonly used as a preservative in the food industry (Todorov, 2009). They act against a broad spectrum of bacterial contaminants. Recent developments of multi-species antibiotic resistance have led to the urgent need for the development of novel bacteriocin from *Lactobacillus* species. This special interest arises from the mechanisms of action of these molecules because lantibiotics act on the pyrophosphate linkage component of the cell wall precursor lipid II. The cell wall is the same target as the clinically used antibiotic vancomycin; however, using a slightly different mechanism of action (Münch et al., 2014). This means that lantibiotics display no cross-resistance with vancomycin, making them a viable option for the screening of new antibiotics to treat methicillin-resistant *Staphylococcus aureus* (MRSA) and vancomycin-resistant enterococci (VRE) (Van Der Heul et al., 2018). Currently, vancomycin is the last line of defense used to treat infections associated with multi-drug resistant Gram-positive bacteria, bindings to the D-alanyl-D-alanine terminus of the lipid II on the outside of the cytoplasmic membrane to inhibit the synthesis of the bacterial cell-wall (Sosio and Donadio, 2006; Van Der Heul et al., 2018).

Co-culture is by far the most used strategy for the regulation of bacteriocin production in *Lactobacillus* (Barefoot et al., 1994; Shimizu et al., 1999; Cheirsilp et al., 2003; Maldonado et al., 2004; Liu et al., 2006; Rojo-Bezares et al., 2007; Di Cagno et al., 2009; Tabasco et al., 2009; Kos et al., 2011; Man et al., 2012; Ge et al., 2014; Chanos and Mygind, 2016; Ariana and Hamedi, 2017). The most general co-culture approach is to up-regulate the biosynthesis of these compounds through the presence of an *auxiliary species* capable of keeping the lactic acid levels low because bacteriocin production is higher in a less acid environment. In the co-culture environment, the auxiliary microbes can be fungi, *Saccharomyces cerevisiae*, *Yarrowia lipolytica*, or *Kluyveromyces marxianus*, and the bacteria *Lactococcus*, *Bacillus*, or *Streptomyces*, which consume lactic and acetic acids and thus do not compete for the carbon source in the media (Shimizu et al., 1999; Cheirsilp et al., 2003; Liu et al., 2006; Tada et al., 2007; Ariana and Hamedi, 2017). Lastly, apart from metabolite consumption by auxiliary microbes, other factors have also been reported to influence bacteriocin yielding in co-culture. These factors include the presence of quorum-sensing messengers (Barefoot et al., 1994; Tabasco et al., 2009; Chanos and Mygind, 2016), such as the peptide autoinducer-2, and the selection of resistant strains as challenge microbes (Maldonado et al., 2004; Rojo-Bezares et al., 2007).

Auxiliary microorganisms also have other modes of action for triggering BCG activation, such as the consumption of metabolites that inhibit BCG (Shimizu et al., 1999; Liu et al., 2006; Ariana and Hamedi, 2017), or the competition for iron *via* siderophore piracy (Traxler et al., 2012). For example, Cheirsilp and co-workers demonstrated that kefiran production in kefir grains happens by a complex and balanced microflora. It consists of lactic acid bacteria, lactose-assimilating yeast, and non-lactose-assimilating yeast. While bacteriocin production is increased by lactic acid-consuming bacteria, the non-lactose-assimilating yeast survives by consuming galactose, which is a product of the lactose-assimilating microorganisms (Cheirsilp et al., 2003).

#### 4.1.3. Bacillus

*Bacillus* is a known spore-forming soil bacteria, and, hence, relies on antagonistic responses toward challenge strains as a competitive advantage during colonization in this environment. When in competition with other soil microbes, antibiotic interactions ensure the development and resource availability of this species, making *Bacillus* a common bacterium for biological control and plant growth-promoting agent (Höfte and Whiteley, 1989; Li et al., 2020). Because of this potential to induce antimicrobial molecules, *Bacillus* species have been broadly studied over the last decade as an inducer strain in co-cultures, in an attempt to be used against phytopathogens. This interest is partially due to the increasing restrictions on the use of chemical pesticides but also because of the high potential of several *Bacillus* species to produce antagonist compounds. In particular, *B. subtilis*, *B. amyloliquefaciens*, *B. pumilus*, and *B. cereus* have been reported to produce novel compounds in co-culture that act as antimicrobials (Trischman et al., 2004; Peterson et al., 2006; Straight et al., 2006; Benitez et al., 2011; Schneider et al., 2012; Moree et al., 2013; Aghcheh and Kubicek, 2015), biosurfactants (Dusane et al., 2011) and inhibitors against fungi and bacteria (Dusane et al., 2011; Wu et al., 2018; Li et al., 2020). Research to date points out that *Bacillus* species produce a wide range of these bioactive substances when in co-culture with Cytophaga-Flavobacterium (Peterson et al., 2006), *Streptomyces* (Straight et al., 2006; Butcher et al., 2007; Schneider et al., 2012), fungi (Dusane et al., 2011; Moree et al., 2013), *Pseudomonas* (Andrić et al., 2021)*, E. coli* (Benitez et al., 2011; Chanos and Mygind, 2016), as well as other *Bacillus* species (Trischman et al., 2004; Dusane et al., 2011). Moreover, this species is also used as a challenge strain, inducing strong metabolic production in competitive species (Watsuji et al., 2007; Shah et al., 2008; Hoefler et al., 2012; Ge et al., 2014; Akone et al., 2016; Chanos and Mygind, 2016; Ebrahim et al., 2016; Sung et al., 2017; Yu et al., 2017; Shin et al., 2018; El-Sayed et al., 2021; Bagheri et al., 2022; Sun et al., 2022). For instance, in 2006, Berleman and co-workers have shown that the proteobacterium *Myxococcus xanthus* responds to *B. subtilis*, *E. coli*, and *Saccharomyces cerevisiae* by altering chemical and developmental patterns. Specifically, the presence of bacteria-induced *M. xanthus* rippling, which is an undulatory movement utilized as a mechanism to consume non-diffusing growth substrates efficiently, maximizing predation and the scavenging for nutrients (Berleman et al., 2006).

Lastly, most reports of commercial *B. thuringiensis* sprays attribute insect death to starvation or direct septicemia. However, Broderick and co-workers demonstrated that *B. thuringiensis* insecticidal activity is also dependent on the presence of signaling molecules. Co-cultivation with *Enterobacter* sp., a bacterium commonly found on the gypsy moth midgut, shows that this midgut bacteria enhances *B. thuringiensis* septicemia, increasing, as a consequence, the insecticidal activity. Furthermore, the absence of these bacteria abolishes *B. thuringiensis* insecticidal activity, evidencing how closely the bacteria contribute to *B. thuringiensis* mortality (Broderick et al., 2006; Shank and Kolter, 2009).

### 4.2. Fungal co-culture: *Aspergillus* and *Fusarium*

**Figure 8:**
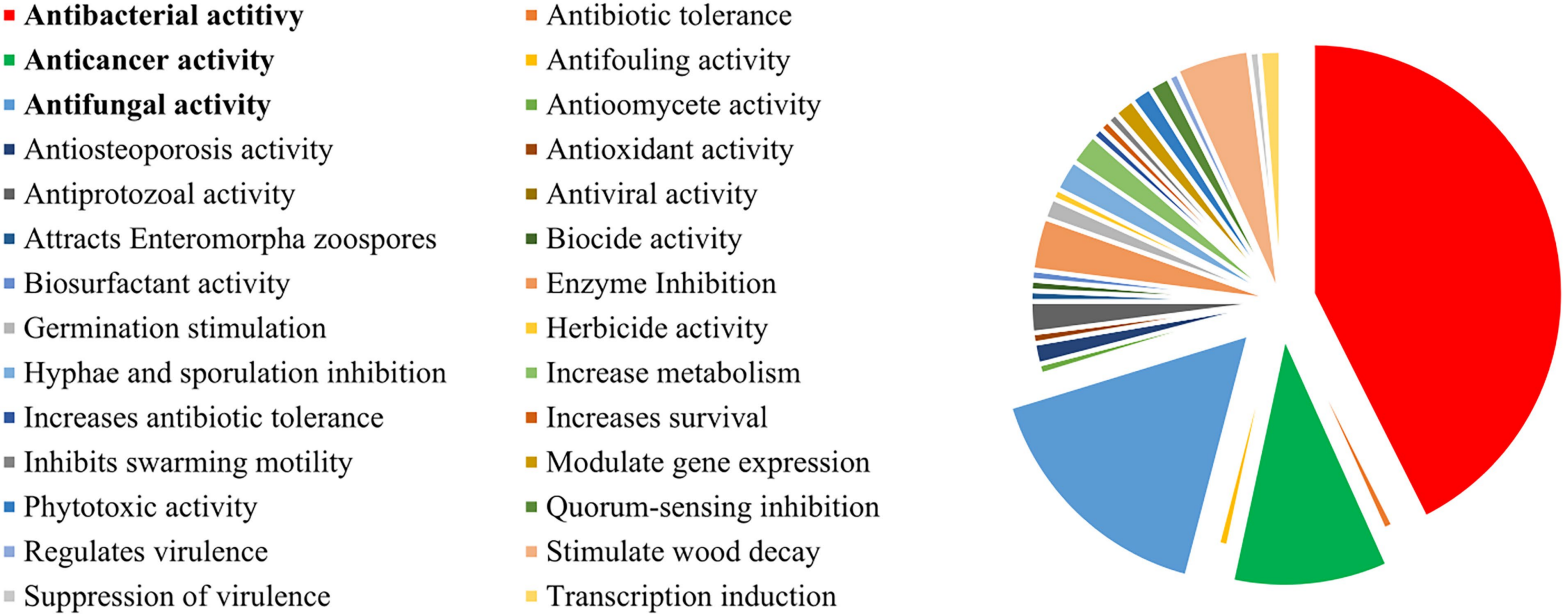
Main biological outcomes reported for co-culture experiments. Biological activity is only reported if not present in the comparative monoculture.

The co-cultures in which the fungi act as inducer microbes are much more diverse in literature than bacteria culturing and consequently have a broader chemical and biological outcome (Figure 8). Among the most studied fungi species, the pathogenic *Fusarium* and *Aspergillus* represent the most used genus, being cultivated with both fungi and bacteria for the enhancement of specialized metabolite and the virulence factors responsible for these species’ pathogenicity.

#### 4.2.1. Aspergillus

*Aspergillus* is well-known for its chemodiversity. During the search for novel bioactive metabolites, the co-culture of *Aspergillus* species with various bacteria and fungi has led to the discovery of a number of newly described and up-regulated compounds, being the most studied inducer fungus reported in mixed fermentations. Examples of co-cultivation of *Aspergillus* species, particularly *A. fumigatus* and *A. niger*, have led to the regulation of amino acids (Liu et al., 2022) and an increased production of alkaloids (Zuck et al., 2011; Rateb et al., 2013), meroterpenes (König et al., 2013), polyketides (Schroeckh et al., 2009; Stroe et al., 2020; Ninomiya et al., 2022), statins (Wang et al., 2022), and peptides (Wu et al., 2015). These molecules have displayed the potential of the *Aspergillus* genome in the production of highly biologically active metabolites that range from antitumoral/cytotoxic (Park et al., 2009; Zuck et al., 2011; El-Sayed et al., 2021), antimicrobial (Meyer and Stahl, 2003; Park et al., 2009; Schroeckh et al., 2009; Moree et al., 2012; König et al., 2013; Ebrahim et al., 2016; Ninomiya et al., 2022; Sun et al., 2022), antiprotozoal (Rateb et al., 2013) to antiosteoporosis activities (Schroeckh et al., 2009).

Other than drug discovery, the chemodiversity of *Aspergilli* has been also extensively studied in opportunistic diseases, given their high pathogenicity in immunocompromised patients. For instance, Konig and collaborators have evaluated the airborne-pathogenic *A. fumigatus*, which is the primary cause of life-threatening invasive and opportunistic mycoses. When in co-culture with *Streptomyces rapamycinicus* isolated from the same environment, they observed activation of previously silent polyketide synthase, resulting in the production of fumicyclines A-B. These meroterpenes were not produced in axenic cultures and display moderate activity against *S. rapamycinicus*, contributing to the understanding of the pathobiology of this human pathogen (König et al., 2013). Moreover, the role of *Aspergillus* in the lungs of cystic fibrosis (*CF*) patients has also been demonstrated by co-cultivation with the bacteria *Pseudomonas aeruginosa*, which often resides together with *A. fumigatus*. Several studies have focused on the development of a methodology for the analysis of both strains in biofilm on a solid medium, characterizing specific microbial metabolites that contribute to lung infection in this polymicrobial niche (Zheng et al., 2015; Neerincx et al., 2016; Reece et al., 2018). Zhao and Yu (2018) demonstrated that, according to the disease stage, the coexistence of *P. aeruginosa* and *A. fumigatus* could lead to mutual inhibition and promotion, regulating their secondary metabolism by VOCs, phenazines, gliotoxin production, and reduced antibiotic sensitivity (Zhao and Yu, 2018).

#### 4.2.2. Fusarium

*Fusarium* is the second most studied fungal genus in co-culture experiments. It concerns filamentous fungi commonly found in the soil, being a normal constituent of the rhizosphere communities of plants (Gordon and Martyn, 1997). In nature, some *Fusarium* strains are highly pathogenic to certain plant species and are responsible for the destruction of crops worldwide, like banana trees (Pegg et al., 2019). Others, however, live symbiotically in the roots, feeding themselves with the plant’s exudate without invading the vascular system or causing disease (Alabouvette et al., 2009).

The infection that some *Fusarium* strains cause in plants is a highly complex process, involving a cascade of regulated steps (Notz et al., 2002; Schouten et al., 2004; van Rij et al., 2005; Bohni et al., 2016). A full understanding of the disease is needed to be able to develop protocols for disease control. One of the primary mechanisms of the infection with these wilt-inducing strains is the release of toxic secondary metabolites, which are often regulated by biotic and environmental factors (Alabouvette et al., 2009). In this context, the use of co-culture with soils that naturally limit the incidence of *Fusarium* wilts allows the evaluation of the physiological conditions that promote this inhibition, as well as the microbial communities that control the expression of toxic secondary metabolites (Bao and Lazarovits, 2001). For example, Minerdi and co-workers have evaluated the relationship between the non-pathogenic and wild *Fusarium* strains with a consortium of bacteria found in a *Fusarium*-wilt suppressive soil, which included the genera *Serratia*, *Achromobacter*, *Bacillus*, or *Stenotrophomonas*. In this study, they demonstrated that sesquiterpenes volatiles, mainly caryophyllenes emitted from the non-pathogenic strain, are capable of negatively influencing mycelial growth and gene expression of virulence genes of pathogenic strains. Interestingly, typing experiments have shown that these same wild *Fusarium* strains, when isolated from the soil bacteria, become pathogenic, causing the same wilt symptoms as the pathogenic strain (Minerdi et al., 2009). Interestingly this is a case in which co-cultivation results in gene silencing.

Other than the ecological role of *Fusarium* in soil, co-cultures with other species have also revealed their great potential as an inducer strain, in which the chemical and biological outcomes are highly dependent on the challenged species. Co-cultivation with *Alternaria tenuissima*, *Sarocladium strictum*, *Saccharopolyspora erythraea, Streptomyces lividans, Epicoccum nigrum*, or *Bacillus subtilis* has led to increased production of trichothecenes (Müller et al., 2012), polyketides (Bohni et al., 2016), decalin-type tetramic acid analogs (Whitt et al., 2014), ennantins (Ola et al., 2013; Moussa et al., 2019; Vásquez-Bonilla et al., 2022), lateropyrone, naphthoquinones, lipopeptides (Moussa et al., 2019) and coumarins (Ola et al., 2013), illustrating the chemodiversity of the *Fusarium* species. Furthermore, *Fusarium* species can also act as a challenge strain, causing induction of several BCGs in *Ustilago maydis* (Estrada et al., 2011), *A. giganteus* (Meyer and Stahl, 2003), *Botrytis cinereal* (Serrano et al., 2017), *A. tenuissima* (Müller et al., 2012), or *P aeruginosa* (Moussa et al., 2020).

## 5. Biological outcomes in mixed cultures

Microbial interactions are the result of a co-evolution process that leads to the adaptation and specialization of the communities (Braga et al., 2016). The specificity of these interactions happens according to the surroundings, regulating the genome of microbial strains to activate different biosynthetic pathways. Based on the effect one microbe has on the other, microbial interactions in co-cultures can be divided into positive, neutral, or negative effects (Alabouvette et al., 2009; Tarkka et al., 2009). These effects vary according to the challenge strain, providing different chemical and biological outcomes. For example, Traxler and collaborators demonstrated the model bacterium *Streptomyces coelicolor* uniquely interacts with other actinomycetes, suggesting an idiosyncratic response from *S. coelicolor*. Using mass spectrometry, they revealed that this Actinomycete produces desferrioxamine with acyl side chains of various lengths triggered by siderophores made by neighboring strains, which affect in different ways the antibiotics, antifungals, and anticancer properties of *S. coelicor* (Traxler et al., 2013).

In terms of bioactivity, most reports target the enhancement of the production and diversity of antimicrobial compounds by antagonistic interactions, evaluating the co-culture systems mainly for this purpose. Hence, it comes as no surprise that the three major outcomes of the publications from 1978 to 2022 have been antibacterial (~45%), antifungal (~18%), and anticancer (~11%) (Figure 8). Several reviews have evaluated the antibiotic potential of co-cultures, and, as such, this topic will only be briefly discussed in this review. For further reference, please check Burgess et al. (1999), Abdalla et al. (2017), Ueda and Beppu (2017), Okada and Seyedsayamdost (2017), and Van Der Heul et al. (2018).

### 5.1. Antimicrobial compounds

The most well-known natural products from microbial sources are, undoubtedly, antibiotics (Demain, 1999). The golden age of those specialized metabolites, which occurred from the early 1940s to the late 1970s, was flared by the penicillin discovery, in 1928, by Alexander Fleming. This revolutionized the treatment of bacterial infections and the control of endemic diseases (Demain and Elander, 1999; Knight et al., 2003; Bader et al., 2010). Most antibiotic scaffolds in use today were discovered in this productive era, including macrolides, glycopeptides, nitroimidazoles, penicillin, sulfonamides, polymyxin, and others.

In the early years, although little understood, co-culture was already applied as a successful strategy to increase microbial metabolite production. In the golden days, mixed fermentation has enhanced yields of known antibiotics penicillin, antimycin, tetracyclines, griseofulvin, bacitracin, levorine, mycoheptin, as well as novel compounds from antimicrobial-inducer strains, such as *Streptomyces, Penicillium* and *Lactobacillus* (Yakovleva and Bulgakova, 1978). The continuous armamentarium of new antibiotics was so overwhelming that it was believed that infectious diseases would be conquered and eradicated by the end of the 20th century (Conly and Johnston, 2005). In reality, between the 1970s and 2000s, the discovery of novel antimicrobial compounds has stalled in the past 20 years. The reason for this decline is first of all the high costs of developing novel antibiotics, which result in neglected interest from the pharmaceutical companies toward new antibiotic treatments. These costs are in part due to problems in finding new leads, the lack of reproducibility of microbial matrices, the identification and constant re-isolation of the same chemotypes, and an ever-increasing bureaucracy of regulatory barriers (Conly and Johnston, 2005).

The lack of discovery of new antibiotic scaffolds, combined with the constant inappropriate use and prescribing, and the extensive agricultural application of these substances have catapulted multidrug resistance of pathogens. Resistance has been found to every antibiotic currently on the market, and bacterial pathogens such as MRSA, drug-resistant *Mycobacterium tuberculosis*, and pan-drug resistant *Pseudomonas aeruginosa* have become the leading health challenges of the century (Fischbach and Walsh, 2009; Okada and Seyedsayamdost, 2017). To overcome this global health emergency, in the last 20 years, new methods have been developed to increase the discovery of novel therapeutic agents (Appelbaum, 2006; Miethke et al., 2021). These strategies often apply genetic and metabolic engineering and are based on bioinformatic analysis, overexpression of biosynthetic genes, cellular biochemistry, and the expression and regulation of targeted genes (Gaisser et al., 2002; Hahn et al., 2006; Demain and Sanchez, 2009). Moreover, methods that do not require any genetic manipulation have also been gaining special attention, particularly for the pleiotropic activation of strains. These cultivation-dependent methodologies include the variation of media composition by OSMAC (Rateb et al., 2011; Romano et al., 2018), co-culture and elicitor screening (Imai et al., 2015; Xu et al., 2017; Moon et al., 2019a,b; Mukai et al., 2020; Zhang and Seyedsayamdost, 2020) and have been implemented complementarily to genetic approaches to efficient increase in both metabolite diversity and yielding (Moody, 2014).

For co-culture, most experiments enhance antibiosis by culturing pathogenic communities with known antibiotic inducer strains (Moody, 2014). However, recent studies have demonstrated that the induction of antibiotic metabolites could be as prevalent as when the challenged strain comes from the same environment. Mearns-Spragg showed that twelve out of the 16 marine strains showed increased antimicrobial activity toward human pathogens *S. aureus*, *E. coli*, and *P. aeruginosa* following exposure (Mearns-Spragg et al., 1998). Similarly, Oh and co-workers have demonstrated that the co-culture of marine-derived fungus *Emericella* sp. and marine actinomycete *Salinispora arenicola* resulted in the enhanced production of antimicrobials emericellamides A and B. This fungal cyclic depsipeptide showed moderate antimicrobial activity against MRSA and weak cytotoxicity against the HCT-116 human colon carcinoma cell lines (Oh et al., 2007).

The concentration of induced metabolite needed to culminate in antibiotic activity has also generated some controversy in the field. There is growing evidence suggesting that antibiotic molecules found in smaller concentrations, can also act as signaling molecules. In their natural environment, subinhibitory levels of these molecules have been shown to mediate cell responses other than death (Davies, 2006; Linares et al., 2006; Fajardo and Martínez, 2008; Moody, 2014). Indeed, it has been shown these sub-lethal levels of various antibiotic metabolites can upregulate the expression of SOS-response and methyl-mismatch repair genes (Mesak et al., 2008), alter virulence factor expression in different bacteria (Linares et al., 2006; Skindersoe et al., 2008), modulate biofilm mass (Starner et al., 2008; Waack and Nicholson, 2018), control colony morphology (Dietrich et al., 2008; Wang et al., 2017) and alter multiple gene promoters in bacteria and fungi (Goh et al., 2002; Mesak et al., 2008; Shank and Kolter, 2009; Amano et al., 2010, 2011; Moody, 2014).

One excellent example of antibiotic hormesis for the activation of BCGs was provided by Xu and co-workers (Xu et al., 2017). In this study, they showed that ivermectin and etoposide, well-known antiparasitic and antibiotic compounds, stimulate the production of other antibiotics in *Streptomyces albus* J1074 at subinhibitory concentrations. Induction by these elicitors led to the elucidation of 14 novel secondary metabolites, including several that arise from crosstalk between BGCs. Variations in the concentrations have confirmed the dose response of these antibiotic elicitors and increased the level of activation up to 150-fold, greatly enhancing secondary metabolite synthesis from a given strain.

The ever-expanding volume of genomic sequencing data continues to facilitate the identification of new antibiotic biosynthetic pathways, allowing access to a vast and unexplored reservoir of metabolic diversity (Demain and Sanchez, 2009). For instance, Ohnishi and co-workers have determined the complete genome sequence of streptomycin-producer *Streptomyces griseus* IFO 13350, in which 34 gene clusters or genes were attributed to the biosynthesis of secondary metabolites (Ohnishi et al., 2008). Nonetheless, they have also observed that secondary metabolism and morphogenesis were only partially activated by *S. griseus* A-factor regulatory cascade, remaining unknown other possible mechanisms for the activation of these cryptic genes. Further application and development of strategies to induce novel antibiotics are needed to shed light on genetic and mechanisms issues in this area.

### 5.2. Negative outcomes

Microbes can interact antagonistically through a multitude of mechanisms. The most common interactions include (1) direct toxicity against the competitive strain by the release of antimicrobial metabolites, (2) degradation of virulence factors, (3) detoxication of toxins, and (4) decrease of toxin production. Each of these mechanisms will be discussed and exemplified individually in this section, although in nature, they should be considered a part of a complex and holistic mechanism for microbial defense.

During antibiosis, microbes tend to produce not one, but a plethora of metabolites, induced by the activation of one or more BCGs. This chemical army is essential to antagonistically act against predators, but also to facilitate communication within the community and prevent the development of pathogen resistance (Tarkka et al., 2009). In 2017, Stierle and co-workers demonstrated this pleiotropic outcome by co-cultivation of *Penicillium fuscum* and *P. camembertii/clavigerum* isolated from acidic and metal-rich waters of Berkeley Pit Lake (Stierle et al., 2017). This mixed fermentation yielded eight new 16-membered-ring macrolides, along with three known antibiotics from different metabolic classes, each of them displaying a variety of antimicrobial activity against MRSA, *Bacillus anthracis*, *Streptococcus pyogenes*, *C. albicans*, and *Candida glabrata*. Moreover, other than direct toxicity, metabolites released in mixed culture could also act as virulence suppressors, regulating morphological transition, toxin production, and antibiotic resistance. To illustrate this, Lopez-Medina and co-workers cultured the pathogenic strains *P. aeruginosa* and *C. albicans* in a neutropenic mouse model of microbial gastrointestinal (Lopez-Medina et al., 2015). In this mammalian model system, the authors demonstrated that the fungus *C. albicans* decreases the virulence of *P. aeruginosa* by the inhibition of the bacterium pyochelin and pyoverdine gene expression, which plays a critical role in iron acquisition.

Most of the metabolites released during antagonistic interspecific interactions are non-enzymatic. However, some enzymes could also be secreted as a response to the presence of another microorganism (Score et al., 1997). In the case of well-studied white-root fungi, co-cultivation targets the production of enzymes that degrades lignin and other xenobiotics (Box 2). But overall, enzymes studied in this experimental setting are mostly described as a resistance mechanism, causing toxin degradation into less toxic compounds (Freitag and Morrell, 1992; Score et al., 1997; Savoie and Mata, 1999; Iakovlev and Stenlid, 2000; Baldrian, 2004; Ferreira Gregorio et al., 2006; Bergmann et al., 2007; Chi et al., 2007; Schouten et al., 2008; Hiscox et al., 2010; Hoefler et al., 2012; Moree et al., 2012; Schneider et al., 2012). For example, in the screening of specialized metabolites between *B. subtilis* and *Streptomyces* sp. Mg1, Hoefler, and co-workers established that, during the early stages of interaction, *B. subtilis* produces surfactin, a cyclic lipopeptide that inhibits the fungus’s aerial growth and spore development (Hoefler et al., 2012). However, IMS showed that in the confrontation zone, there was an enhanced production of the enzyme surfactin hydrolase, acting on this lipopeptide to produce a hydrolyzed molecule that was not active to inhibit the fungus aerial growth.

**BOX 2. Enzymatic Regulation in Co-culture of Basidiomycetes Rot Fungi.**

Enzyme activation is particularly important in the study of white-root fungi. These microbes are industrially used in co-culture to produce enzymes that can degrade lignin and other xenobiotics. This biochemical ability for enzymatic production is mediated by several unknown factors. However, physical contact and the secretion of volatiles seem to play crucial roles to increase their production (Baldrian 2004; Hynes et al., 2007; Peiris et al., 2008).

Specifically, enzymatic variation in the basidiomycetes rot fungi are of great biotechnological interest due to their ability to degrade lignin and other xenobiotic, such as pesticides, polyaromatic hydrocarbons, polychlorinated biphenyls nitro explosives, and other toxic chemicals. These enzymes are currently used for the treatment of industrial dye effluents, and the biodegradation of organic pollutants and waste (Gao et al., 2010).

✓ Co-culture has been repetitively used to enhance enzymatic abilities, increasing the activity of oxidative enzymes laccase, manganese peroxidase (MnP), manganese-repressed peroxidase (MRP) and lignin peroxidase (LiP).
✓ The extent of the increase in activity differs, depending on the enzyme-producing ability of the species, and the microbial interaction.
✓ *Trametes versicolor* is the most studied white-rot basidiomycetes. This species has shown pronounced enzyme activity when competing against several fungi and bacteria, including *Stereum gausapatum*, *Daldinia concentrica*, *Bjerkandera adusta*, *Fomes fomentarius*, *Hypholoma fasciculare* (Hiscox et al., 2010), *Pleurotus ostreatus* (Baldrian, 2004)*, Trichoderma harzianum* (Freitag and Morrell, 1992), *Acremonium sphaerospermum*, *Fusarium reticulatum*, *Humicola grisea*, *Penicillium rugulosum*, *Bacillus subtilis*, *Escherichia coli*, *Endomyces magnusii*, soil fungi (Baldrian, 2004).

Although microbial defense strategies are complex and multifactorial, only a few studies detail mechanisms that do not involve the release of specialized metabolites or enzymes in co-culture. For example, in 2008, Schouten and collaborators demonstrated that the resilient pathogenic fungi *Botrytis cinerea* employs, other than phytotoxic metabolites, two different strategies against other pathogenic fungi (Schouten et al., 2008). When in co-culture, *B. cinerea* resists the broad-spectrum phenolic antibiotic 2,4-diacetylphloroglucinol (2,4-DAPG) by both degradative and non-degradative defense mechanisms. Results showed that while the efflux pump BcAtrB prevents antibiotic accumulation in the cell, the up-regulation of extracellular laccase degrades the remaining antibiotic, creating a double resistance mechanism against these exogenous toxic compounds (Schouten et al., 2008; Tarkka et al., 2009). *Fusarium* species have also been shown to give a multifactorial response against antagonist microbes. The human and phytopathogenic *F. oxysporum* has been shown to interrupt 2,4-DAPG antibiotic activity by deacetylation of this compound into the less toxic derivatives monoacetyl-phloroglucinol and phloroglucinol (Schouten et al., 2004). However, non-degradative mechanisms also act on their cross-domain signaling, forcing the antagonist microbe to attenuate toxin production. For instance, fusaric acid is a polyketide produced by different species of *Fusarium* and in mixed cultures, up-regulation of this compound increases antimicrobial activity (Bohni et al., 2016) while also repressing the production of toxins (Notz et al., 2002; Duffy et al., 2003; van Rij et al., 2005). In fact, studies that focused on the toxin suppression of picolinic acid derivatives showed that *Fusarium*, when in co-culture with biocontrol strain *Pseudomonas fluorescens*, increased fusaric acid production, enhancing the suppression of key virulence factor of the biocontrol strains, such as phenazine-1-carboxamide, auto-inducer N-hexanoyl-L-homoserine lactone (C6-HSL) (van Rij et al., 2005) and 2,4-diacetylphloroglucinol (DAPG) (Notz et al., 2002).

### 5.3. Positive outcomes

Positive interactions are systematically overlooked during co-culture experiments that focus on drug discovery. Only a few reports have been made about the chemical and biological potential of these communication systems. Ueda and Beppu have studied the interaction between different *Streptomyces* strains, targeting to identify intra- and interspecific signaling during mixed cultivation (Ueda and Beppu, 2017). In this study, the authors revealed that interspecific stimulation of antibiotics occurs at a higher frequency in mutualistic interaction rather than antagonistic. That up-regulation was probably due to the developmental induction of the challenge microbe in the receiver strain. Notably, the production of siderophores, ionophores, and other ATP synthesis inhibitors has shown great potential to activate inter-species antibiotic production in *Streptomyces*, encouraging the exploration of mutualistic growth to elicit the production of bioactive specialized metabolites.

The use of mechanisms to preserve beneficial microbes is also a common component of host-microbe mutualisms and shows how wild microbes confer benefits to their hosts. For example, Scott and co-workers demonstrated how actinomycetes could protect the symbiosis between a southern pine beetle *Dendeoctonus frontalis*, and *Entomocorticium* sp. (Scott et al., 2008). This fungus is known to inhabit the beetle storage compartment and helps nourish the beetles’ larvae. Using co-cultivation, Scott and co-workers demonstrated that these bacteria from the same storage compartment of the beetle produce a linear polyene peroxide that selectively suppresses the antagonistic fungus *Ophiostoma minus*, while displaying no significant effect in the beneficial fungus *Entomocorticium sp.* (Scott et al., 2008; Shank and Kolter, 2009).

Polymicrobial environments are also a common source of positive and mutualistic effects in plants. For example, the plant rhizosphere contains a large microbiome that feeds on the root exudates. In exchange for nutrients, this massive source of microorganisms promotes plant growth and defense by a multitude of unknown mechanisms. Maier and collaborators reported a mutualistic relationship between the basidiomycete *Amanita muscaria*, and actinomycetes isolated from its rhizosphere (Maier et al., 2004). In co-cultivation, some of these filamentous bacteria promoted the growth of fungal hyphae, whereas others inhibited the growth of pathogenic fungi. Indeed, examples of plants, insects, and animals that engage with Actinomycetes in protective symbiosis (Seipke et al., 2012; van der Meij et al., 2017) are numerous, illustrating the tip-of-the-iceberg of ecological interaction in host environments.

Opportunistic infections are also polymicrobial in human health and symbiosis between these microbes shapes the pathogenicity and the prognostic of the diseases. For instance, *P. aeruginosa* and *S. aureus* are human opportunistic pathogens commonly co-isolated from medical equipment, skin, eyes, and respiratory tract of people with cystic fibrosis (*CF*). For example, Hoffman and collaborators analyzed the sputum of *CF* patients to determine the mutualistic relationship between these species (Hoffman et al., 2006). They revealed that *P. aeruginosa* produces 4-hydroxy-2-heptylquinoline-*N*-oxide, a quinoline that suppresses *S. aureus* respiration, protecting it from death by commonly used aminoglycoside antibiotics. Furthermore, they also showed that prolonged growth of both pathogenic bacteria led to the selection of specific *S. aureus* strains, which are known for both aminoglycoside resistance and persistence in chronic infections.

## 6. Conclusion and perspectives

Microbes play a crucial role in drug discovery screenings. In this sense, cultivation-dependent methods that cause pleiotropic regulation of metabolites emerge as promising candidates for the screening of microbial diversity. In the cases where the microbe is unknown or when the biosynthetic pathways rely on reciprocal dependence of more than one BCG, these methods have shown to be more useful than the genetic-dependent ones, providing a global and unbiased alteration of the microbial physiology.

To date, over 300 compounds with a diversity of biological activities have been identified in co-cultivation. Recent strategies of unusual pairwise or microbial consortia promise to increase the number of reported molecules exponentially. Moreover, the diversity of microbial species encountered in nature makes co-culture experiments a standard choice for the untargeted evaluation of specialized metabolites. Scaling law predicts that Earth is home to trillions of microbial species, offering unlimited opportunities for combining a nearly infinite number of strains. All these factors, combined with increasingly sophisticated analytical techniques for chemical detection and quantification, illustrate the potential of mixed fermentation in activating gene clusters that encode promising and unexplored secondary metabolites.

Accumulating evidence indicates that co-cultivation is dependent on both biotic and abiotic parameters. A greater understanding of the underlying functional interactions is also critical for deriving general mechanisms. As such, there is an urgent need to standardize the detailed experimental procedures and the possible trigger factors during communication. This information could shed light on the intimate process involving metabolite production. Unraveling these conditions includes the understanding of abiotic factors interferences, global regulators, and elicitors, providing a systematic investigation of the microbial metabolome profiles.

In most reported experiments, antagonistic interaction is the driving force that promotes the biosynthesis of biologically active compounds. However, the evaluation of mutualistic and multifactorial relations in nature has also resulted in a significant variation in the metabolic profile. These positive interactions remain a neglected strategy in most reported studies. Changing our view of microbial intercellular signaling could enable the use of the right conditions for cells to communicate and, hence, generate new bioactive molecules.

The development of high throughput methodologies is needed to overcome the continuous multidrug resistance in pathogenic strains and satisfy the constant need for new drug candidates. Also, although the methods to activate silenced genes have collectively begun to illuminate the massive untapped trove of new metabolites, few studies have shown the precise trigger factors responsible for gene activation. Current research suggests that one of the most important trigger factors is cell-to-cell contact. However, other mechanisms have also been described to induce BCGs, such as the presence of elicitor molecules.

## Author contributions

DS organized the table, created all figures and wrote the first draft of the manuscript and Supplementary material. DS and IC-G contributed to the conception and design of the study. All authors contributed to the manuscript revision, read, and approved the submitted version.

## Funding

This work was supported by the Fundação de Amparo à Pesquisa do Estado de São Paulo [grants number 2013/07600-3, CibFar CEPID; numbers 2014/05935-0, and 2017/06446-2, Ph.D]. Additional research grants were awarded by Conselho Nacional de Desenvolvimento Científico e Tecnológico – CNPq [grant number 449523/2014-4] and by Coordenação de Aperfeiçoamento de Pessoal de Nível Superior - Brasil (CAPES), Instituto Nacional de Ciência e Tecnologia (INCTBioNat-CNPq/FAPESP) [grant number 465637/2014-0].

## Conflict of interest

The authors declare that the research was conducted in the absence of any commercial or financial relationships that could be construed as a potential conflict of interest.

## Publisher’s note

All claims expressed in this article are solely those of the authors and do not necessarily represent those of their affiliated organizations, or those of the publisher, the editors and the reviewers. Any product that may be evaluated in this article, or claim that may be made by its manufacturer, is not guaranteed or endorsed by the publisher.

## Figures and Tables

**Table 1:** 

ATP	Adenosine Triphosphate
BGC	Biosynthetic gene cluster
*CF*	Cystic Fibrosis
Challenge strain	The strain that is interacting with the inducer. This includes all the microbes that provide the biotic interaction for the induction as well as *auxiliary species*, which are challenge microbes that alter media composition by the selective consumption of nutrients, aiding the inducer strain in the production of given metabolites.
DAPG	Diacetylphloroglucinol
DDA	agar-Disc Diffusion Assay
DMSO	Dimethylsulfoxide
DoE	Design of Experiment
DSF	Diffusible Signal Factor
GC–MS	Gas Chromatography coupled with Mass Spectrometry
HSL	Homoserine Lactone
IMS	Matrix-Assisted Laser Desorption Ionization (MALDI) time-of-flight (TOF) imaging mass spectrometry
Inducer strain	Microorganisms that produce the specialized metabolites because of co-cultivation. This is because these strains induce their biosynthetic pathways to produce these compounds.
LC–MS	Liquid Chromatography coupled with Mass Spectrometry
LiP	Lignin Peroxidase
MALDI-TOF	Matrix-Assisted Laser Desorption Ionization – Time of Flight
MIC	Minimum Inhibitory Concentration
MnP	Manganese Peroxidase
MRP	Manganese-repressed Peroxidase
MRSA	Methicillin-resistant *Staphylococcus aureus*
MS	Mass Spectrometry
NMR	Nuclear Magnetic Resonance spectroscopy
NRPS	Nonribosomal Peptide Synthetase
OSMAC	One Strain, Many Compounds
PKS	Polyketide Synthase
SPME	Solid Phase Microextraction
VOC	Volatile Organic Compounds
VRE	Vancomycin-resistant enterococci
