# Supplementary material for: Enhancing chemical and biological diversity by co-cultivation
Source: Front Microbiol. 2023 Feb 1;14:1117559. doi: 10.3389/fmicb.2023.1117559 (PMC9928954; doi:10.3389/fmicb.2023.1117559)
Supplement: Supplementary file 2 [file Data_Sheet_1.PDF]

Enhancing Chemical and Biological Diversity by Co-cultivation

Denise M. Selegato[1,j,*], Ian Castro-Gamboa[1]

[1] Nucleus of Bioassays, Biosynthesis, and Ecophysiology of natural products (NuBBE), Institute of Chemistry, Brazil

[j] Current address: Zimmermann Group, Structural and Computational Biology Unit, European Molecular Biology Laboratory (EMBL), Meyerhofstraße 1, 69117, Heidelberg, Germany.

[*] Corresponding author: Denise Medeiros Selegato. E-mail: denise.selegato@embl.de

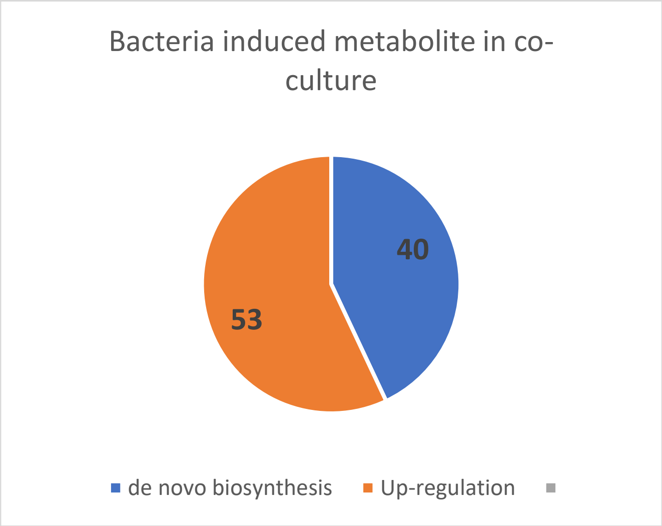
Bacteria induced metabolite in co-culture
40
53
de novo biosynthesis
Up-regulation

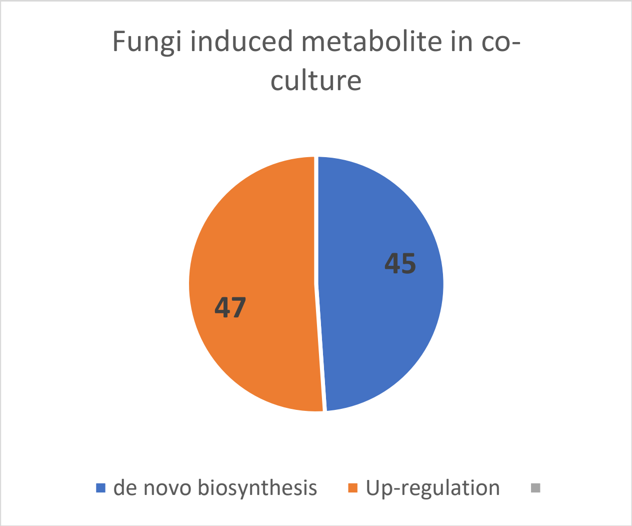
Fungi induced metabolite in co-culture
45
47
de novo biosynthesis
Up-regulation

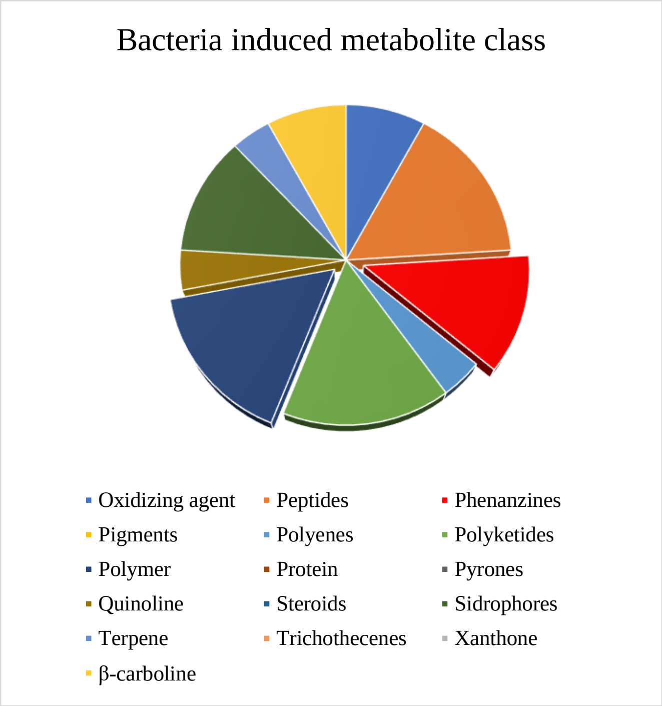
Bacteria induced metabolite class
Oxidizing agent
Peptides
Phenanzines
Pigments
Polyenes
Polyketides
Polymer
Protein
Pyrones
Quinoline
Steroids
Sidrophores
Terpene
Trichothecenes
Xanthone
β-carboline

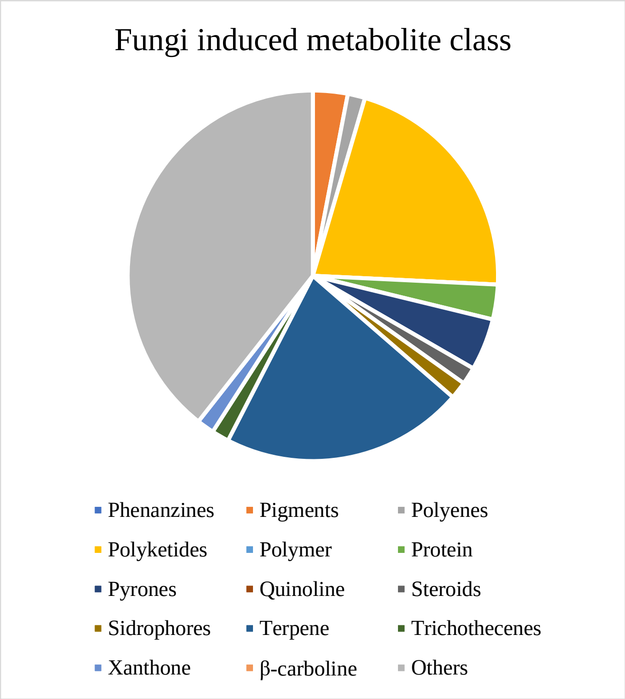
Fungi induced metabolite class
Phenanzines
Pigments
Polyenes
Polyketides
Polymer
Protein
Pyrones
Quinoline
Steroids
Sidrophores
Terpene
Trichothecenes
Xanthone
β-carboline
Others

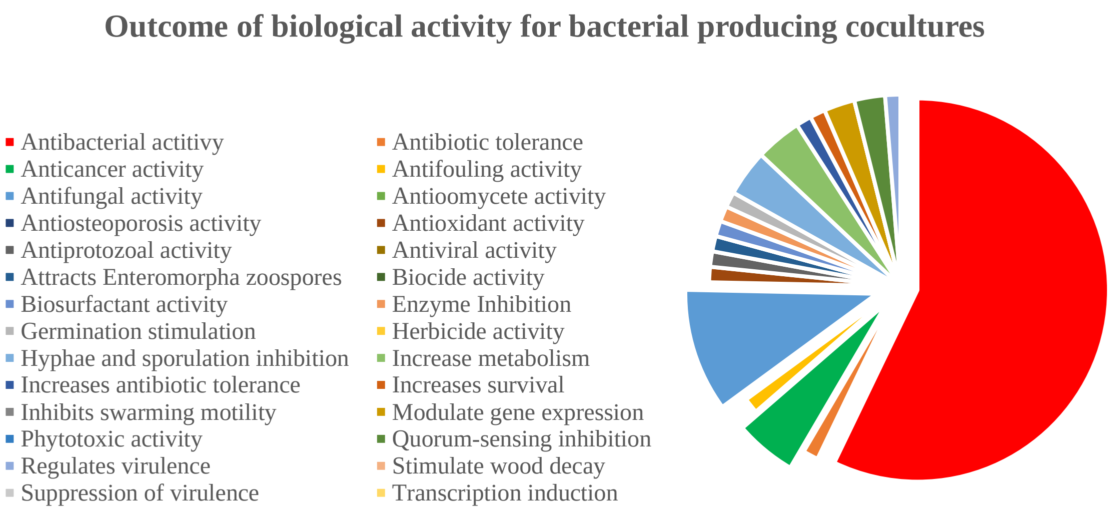
Outcome of biological activity for bacterial producing cocultures
Antibacterial actitivy
Antibiotic tolerance
Anticancer activity
Antifouling activity
Antifungal activity
Antioomycete activity
Antiosteoporosis activity
Antioxidant activity
Antiprotozoal activity
Antiviral activity
Attracts Enteromorpha zoospores
Biocide activity
Biosurfactant activity
Enzyme Inhibition
Germination stimulation
Herbicide activity
Hyphae and sporulation inhibition
Increase metabolism
Increases antibiotic tolerance
Increases survival
Inhibits swarming motility
Modulate gene expression
Phytotoxic activity
Quorum-sensing inhibition
Regulates virulence
Stimulate wood decay
Suppression of virulence
Transcription induction

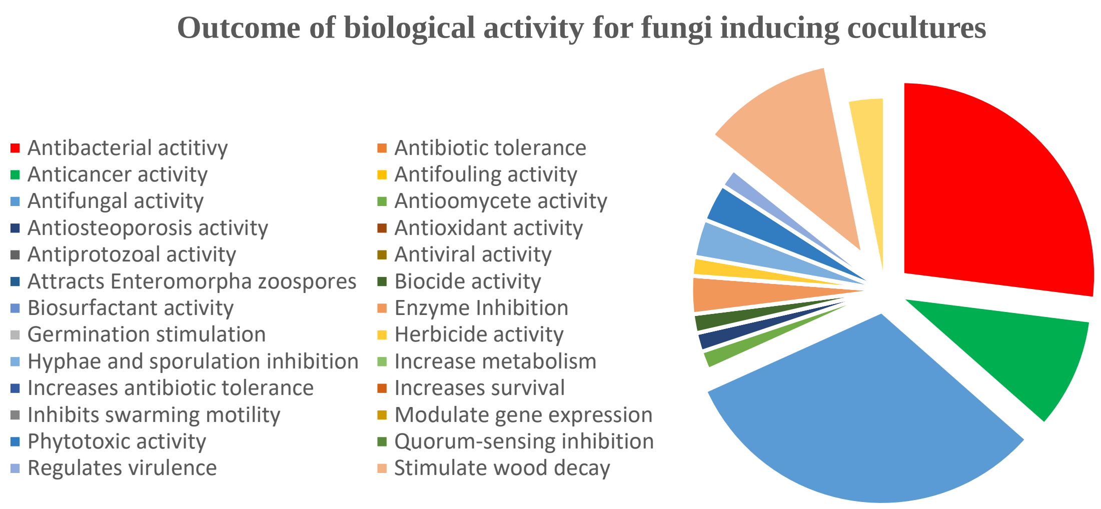
Outcome of biological activity for fungi inducing cocultures
Antibacterial actitivy
Antibiotic tolerance
Anticancer activity
Antifouling activity
Antifungal activity
Antioomycete activity
Antiosteoporosis activity
Antioxidant activity
Antiprotozoal activity
Antiviral activity
Attracts Enteromorpha zoospores
Biocide activity
Biosurfactant activity
Enzyme Inhibition
Germination stimulation
Herbicide activity
Hyphae and sporulation inhibition
Increase metabolism
Increases antibiotic tolerance
Increases survival
Inhibits swarming motility
Modulate gene expression
Phytotoxic activity
Quorum-sensing inhibition
Regulates virulence
Stimulate wood decay

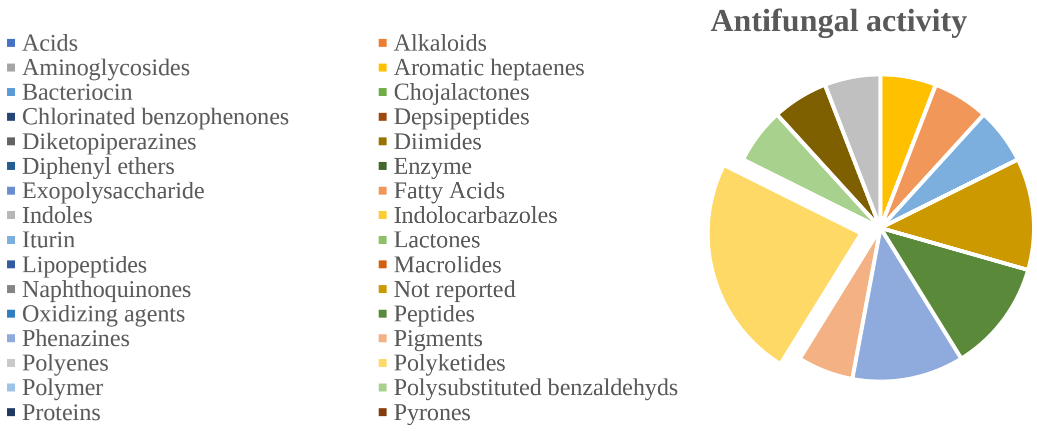
Antifungal activity
Acids
Alkaloids
Aminoglycosides
Aromatic heptaenes
Bacteriocin
Chojalactones
Chlorinated benzophenones
Depsipeptides
Diketopiperazines
Diimides
Diphenyl ethers
Enzyme
Exopolysaccharide
Fatty Acids
Indoles
Indolocarbazoles
Iturin
Lactones
Lipopeptides
Macrolides
Naphthoquinones
Not reported
Oxidizing agents
Peptides
Phenazines
Pigments
Polyenes
Polyketides
Polymer
Polysubstituted benzaldehyds
Proteins
Pyrones

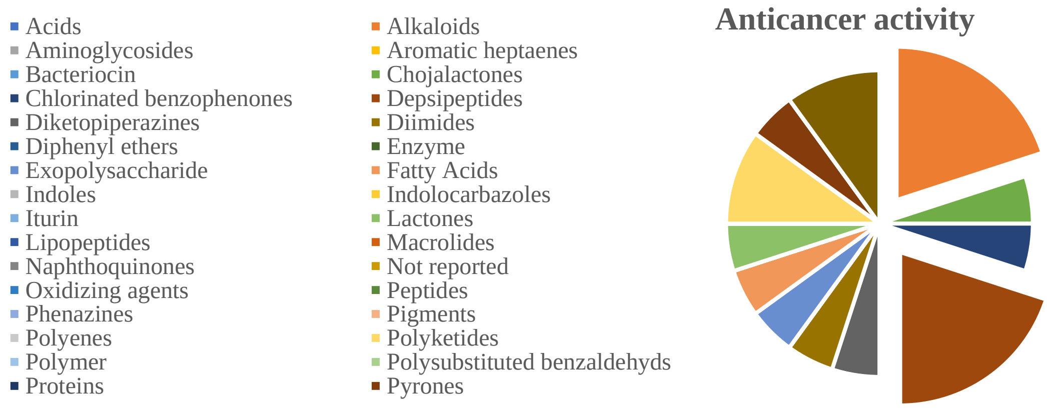
Anticancer activity
Acids
Alkaloids
Aminoglycosides
Aromatic heptaenes
Bacteriocin
Chojalactones
Chlorinated benzophenones
Depsipeptides
Diketopiperazines
Diimides
Diphenyl ethers
Enzyme
Exopolysaccharide
Fatty Acids
Indoles
Indolocarbazoles
Iturin
Lactones
Lipopeptides
Macrolides
Naphthoquinones
Not reported
Oxidizing agents
Peptides
Phenazines
Pigments
Polyenes
Polyketides
Polymer
Polysubstituted benzaldehyds
Proteins
Pyrones

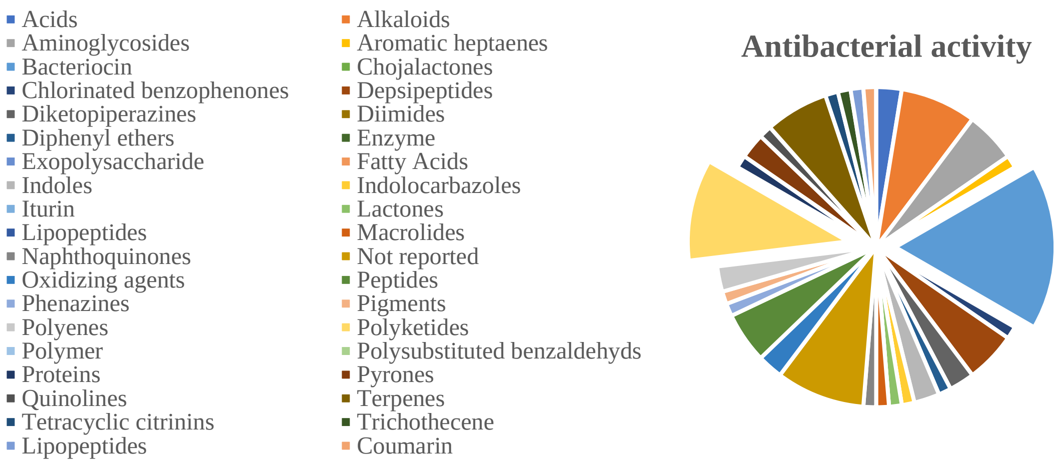
Antibacterial activity
Acids
Alkaloids
Aminoglycosides
Aromatic heptaenes
Bacteriocin
Chojalactones
Chlorinated benzophenones
Depsipeptides
Diketopiperazines
Diimides
Diphenyl ethers
Enzyme
Exopolysaccharide
Fatty Acids
Indoles
Indolocarbazoles
Iturin
Lactones
Lipopeptides
Macrolides
Naphthoquinones
Not reported
Oxidizing agents
Peptides
Phenazines
Pigments
Polyenes
Polyketides
Polymer
Polysubstituted benzaldehyds
Proteins
Pyrones
Quinolines
Terpenes
Tetracyclic citrinins
Trichothecene
Lipopeptides
Coumarin

Type	Inducer microbe	Culture Medium	Challenge microbe	Compound	Compound class	Induction type	Biological activity	Reference
F/F	Serpula *lucrymans*	Solid	*Trichoderma* sp (3 isolates)	laccase	Enzymes	*de novo* biosynthesis	Stimulate wood decay	(Score et al. 1997)
B/F and B/B	*Bacillus* sp. S3, *B. pumilus* S8, *B. licheniformis* D1	Liquid	*Candida albicans, Yarrowia lipolytica, Pseudomonas aeruginosa, Bacillus pumilus*	not identified	Not identified	Up-regulation	Antifungal activity against *Y. lipolytica*	(Dusane et al. 2011)
B/B	S*treptomyces* sp (76 species)	Solid	*Bacillus subtilis* ATCC6633	not identified	Not identified	Up-regulation	Antibiotic activity	(Ueda et al. 2000)
F/F	*Acremonium* sp. Tbp-5	Solid	*Mycogone rosea* DSM 12973	acremostatins A–C	Lipoaminopeptides	*de novo* biosynthesis	No reports	(Degenkolb et al. 2002)
B/F	*Actinomyces levoris*	Solid	*Candida tropicalis*	levorin	Aromatic heptaene	*de novo* biosynthesis	Antifungal and antibiotic activity	(Yakovleva and Bulgakova 1978)
F/F	*Alternaria tenuissima*	Liquid and Solid	*Nigrospora sphaerica*	stemphyperylenol, alterperylenol	Polyketides	*de novo* biosynthesis	Stemphyperylenol shows antifungal activity	(Chagas et al. 2013)
F/F	*Antrodiella citrinella, Flammulina velutipes, Fomitopsis pinicola, Heterobasidion annosum, Junghuhnia collabens, Phlebia centrifuga, Phlebiopsis gigantea, Resinicium bicolor*	Solid	*Antrodiella citrinella, Flammulina velutipes, Fomitopsis pinicola, Heterobasidion annosum, Junghuhnia collabens, Phlebia centrifuga, Phlebiopsis gigantea, Resinicium bicolor*	laccase	Enzymes	Up-regulation/*de novo* biosynthesis	Stimulate wood decay	(Iakovlev and Stenlid 2000)

B/B	*Aspergillus austroafricanus*	Liquid	*Bacillus subtilis* or *Streptomyces lividans*	austramide, violaceol I, violaceol II, diorcinol	Diphenyl ethers	*de novo* biosynthesis/Up-regulation	Violaceol I-II has antibacterial active against *Staphylococcus aureus* (ATCC 700699); Diorcinol has moderate antibacterial activity against *B. subtilis.*	(Ebrahim et al. 2016)
F/B	*Aspergillus fumigatus*	Liquid	*Streptomyces rapamycinicus*	fumicyclines A-B	Meroterpenoids	*de novo* biosynthesis	Moderate antibacterial activity against *S. rapamycinicus.*	(König et al. 2013)
B/F or F/B	*Aspergillus fumigatus*	Solid	*Pseudomonas aeruginosa*	1-Hydroxyphenanzine, 1-methoxyphenazine, phenazine-1-sulfate, triacetylfusarinine C [Al3+] and triacetylfusarinine C [Fe3+], triacetylfusarinine C, fusarinine C and phenazine dimers	Phenanzines	Biotransformation	1-hydroxyphenanzine and 1-methoxyphenazine suppressed fungal growth; siderophores triacetylfusarinine C, triacetylfusarinine C [Al3+] and triacetylfusarinine C [Fe3+] stimulate the capturing of iron	(Moree et al. 2012)
F/B	*Aspergillus fumigatus*	Liquid	*Streptomyces bullii*	ergosterol, brevianamide F, spirotryprostatin A, 6-methoxy spirotryprostatin B, fumitremorgin C and its 12,13-dihydroxy derivative, fumitremorgin B, verruculogen, 11-O-methylpseurotin A, 11-O-methylpseurotin A2, emestrins A-B	Diketopiperazine alkaloids	*de novo* biosynthesis	High toxicity and antiparasitic activity against *Trypanosoma brucei brucei* and *Leishmania donovani* (except brevianamide F and 11-O-methylpseurotin A)	(Rateb et al. 2013)
F/B	*Aspergillus fumigatus*	Liquid	*Streptomyces peucetius*	Fumiformamide, N,N′-((1Z,3Z)-1,4-bis(4-methoxyphenyl)β-1,3-diene-2,3-diyl)diformamide, two known N-formyl derivatives and xanthocillin analogue BU-4704	Alkaloids	*de novo* biosynthesis	N,N′-((1Z,3Z)-1,4-bis (4-methoxyphenyl)β-1,3-diene-2,3-diyl) diformamide has cytotoxic activity	(Zuck et al. 2011)
F/B	*Aspergillus fumigatus* KMC-901	Liquid	*Sphingomonas sp* KMK-001	glionitrin A	Diketopiperazine alkaloid	*de novo* biosynthesis	Antibacterial activity against MRSA, antitumor activity against human cancer cell	(Park et al. 2009)

							lines HCT-116, A549, AGS, and DU145	
F/F	*Aspergillus giganteus*	Liquid	*Aspergillus niger*	antifungal protein (AFP)	Protein	Down-regulation	Antifungal protein (AFP) has antifungal activity against various filamentous fungi	(Meyer and Stahl 2003)
F/F	*Aspergillus giganteus*	Liquid	*Fusarium* oxysporum	antifungal protein (AFP)	Protein	Up-regulation	Antifungal protein (AFP) has antifungal activity against various filamentous fungi	(Meyer and Stahl 2003)
F/F	*Aspergillus nidulans*	Liquid	Actinomycetes (58 soil-dwelling bacteria) and *Escherichia coli*	orsellinic acid, lecanoric acid, F-9775A and F-9775B	Archetypal polyketides and pentacyclic triterpenes	*de novo* biosynthesis	F-9775A and F-9775B has antiosteoporosis activity; orsellinic acid has antimicrobial activity; lecanoric acid inhibits ATP synthesis and electron transfer and has antimicrobial activity	(Schroeckh et al. 2009)
B/F and B/B	*Bacillus pumilus*	Liquid	Serratia *marcescens, Bacillus pumilus* or *Bacillus licheniformis*	not identified	Not identified	Up-regulation	Increase in biosurfactant activity	(Dusane et al. 2011)
B/B	*Bacillus amyloliquefaciens* LBM 5006	Liquid	*Escherichia coli* ATCC 25922	peptides	Bacteriocin/peptide	*de novo* biosynthesis	Antibacterial activity	(Benitez et al. 2011; Chanos and Mygind 2016)
B/F	*Bacillus amyloliquefaciens*	Solid	*Aspergillus fumigatus* and *Aspergillus niger*	Iturin	Lipopetides	*de novo* biosynthesis	Antifungal activity	(Moree et al. 2013)
B/B	*Bacillus cereus* UW85	Liquid	*Cytophaga-Flavobacterium* (CF)	peptidoglycan from vegetative cells	Polymer	Up-regulation	Increase the growth of CF bacteria	(Peterson et al. 2006)
B/B	*Bacillus* isolate strain UA-094	Liquid	*Bacillus* isolate strain UA-086	indole and cyclo(Phe-Pro) diketopiperazine	Indole and diketopiperazine	Up-regulation	Antibacterial activity	(Trischman et al. 2004)
B/B	*Bacillus subtilis*	Solid	*Streptomyces griseus*	lactonase-homologous Protein	Protein	*de novo* biosynthesis	Antibacterial activity: inhibits development and	(Schneider et al. 2012)

							streptomycin production in *S. griseus.*	
B/B	*Bacillus subtilis*	Solid	*Streptomyces coelicolor* and *Streptomyces avermitilis*	bacillaene	Polyene	Up-regulation	Delays the production of pigmented antibiotics in *S. coelicolor;* Antibiotic activity against *S. avermitilis* (inhibits the growth).	(Straight et al. 2006; Butcher et al. 2007)
B/B	*Bacillus subtilis*	Liquid	*Streptomyces coelicolor*	surfactin	Lipopeptide surfactant	Up-regulation	Inhibition of aerial hyphae and sporulation of *S. coelicolor*	(Straight et al. 2006)
F or B/B	*Bacillus subtilis, Escherichia coli, Saccharomyces cerevisiae*	Liquid	*Myxococcus xanthus* (proteobacterium)	peptidoglycan	Polymer	Up-regulation	Induction of rippling (ondulatory movement) in *M. xanthus*	(Berleman et al. 2006)
B/F and B/B	*Bacillus* sp. S3, *B. pumilus* S8, *B. licheniformis* D1, and *Serratia marcescens* V1	Liquid	*Candida albicans, Yarrowia lipolytica, Pseudomonas aeruginosa* and *Bacillus pumilus*	not identified	Not identified	Up-regulation	Antibacterial activity against *P. aeruginosa* and *B. pumilus*	(Dusane et al. 2011)
B/B	*Bacillus* sp. S3	Liquid	*Pseudomonas aeruginosa*	not identified	Not identified	Up-regulation	Quorum-sensing inhibition	(Dusane et al. 2011)
B/P	bacterial biofilms	Liquid	*Enteromorpha (*green seaweed)	N-acylhomoserine lactone (AHL)	Quroum sensing fatty acid (DSF)	Up-regulation	AHL attracts *Enteromorpha* zoospores	(Joint et al. 2002)
F/F	*Bionectria ochroleuca*	Solid	*Trichophyton rubrum*	4″-hydroxysulfoxy-2,2″-dimethylthielavin P	Polyketide	*de novo* biosynthesis	No reports	(Bertrand et al. 2013)

F/F	*Botrytis cinerea*	Solid	*Candida albicans, Aspergillus fumigatus, Colletotrichum acutatum, Fusarium proliferatum,* and *Magnaporthe grisea*	11-hydroxy-dehydro-botrydienol, botrytisic acid A, botrytisic acid B, dehydro-botrydienol, 3-hydroxy-4-oxocyclofarnesa-2,5,7,9-tetraen-11,8-olide, norbotrydialone acetate, botrydienal phytotoxin, 10-oxo-dehydro-dihydro-bodtrydial, hydratedbotryenalol, and dehydro-botrydienal, 2-(-hydroxy-5-methoxyphenoxy)acrylic acid, integrastin B, palmarumycin C15, australifungin, botryendial phytotoxin, cissetin, 3-O-acetyl-botcineric acid, equisetin, enniantin I, enniantin G, 11,12-hydroxy-eudesm-4-en-3-one, cordyol C, illudin C3, illudinic acid, integrastin B, 7-chrolo-6-methoxymellein, cis-4-hydroxy-6-deoxy-scytalone or xylarine, citreoviridin licicolinic acid A, 4-hydroxy-5-methylmellein, chaetoxanthone C, Cordyol C, mycorrhizin A, palmarumycins C7 and C15, chaetoxanthone C, diorcinol, destruxin A1/A4, antibiotic FR-49175, bis-dethio-bis-methyl-thio-gliotoxin, 3-hydroxymellein, 3,4-dihydro-3,8-dihydroxy-3-methyl-1H2-benzopyran-1-one, pleuromutilin, cyclopiazonic acid	Fatty acids, enniantins, and others	Up-regulation/*de novo* biosynthesis	Antifungal activity and cytotoxicity	(Serrano et al. 2017)

F/F	*Serpula lacrymans* and *Coniophora puteuna*	Solid	*Trichoderma* spp and *Scytalidium* FY	peroxidase	Enzymes	Up-regulation	Stimulate wood decay	(Score et al. 1997)
F/F	*Coniophora puteuna*	Solid	*Scytalidium* FY	laccase	Enzymes	Up-regulation	Stimulate wood decay	(Score et al. 1997)
B/F	*Burkholderia cenocepacia*	Liquid	*Candida albicans* and *Xanthomonas campestris* pv. campestris	cis-2-dodecenoic acid (BDSF)	α,β Unsaturated fatty acid (DSF)	Up-regulation	BDSF restores the biofilm and extracellular polysaccharide production phenotypes of *X. campestris* and inhibits *C. albicans* germ tube formation.	(Boon et al. 2008)
B/B and B/P	*Burkholderia gladioli*	Liquid	*Burkholderia seminalis* and orchid	extracellular polysaccharides	Polysaccharides	Up-regulation	Polysaccharide alters hormone metabolism	(Araújo et al. 2016)
B/F	*Burkholderia gladioli*	Solid	*Rhizopus microsporus*	enacyloxin Iia, iso-enacyloxin Iia, enacyloxin IIIa and iso-enacyloxin IIIa	Polyketides	*de novo* biosynthesis	Antifungal and antibacterial activity	(Ross et al. 2014)
F/F	*Candida albicans*	Solid	*Trichophyton rubrum, Trichophyton mentagrophytes, Trichophyton terrestre* and *Microsporum gypseu*m. (other common dermatophytes)	(3R,6E)-2,3-dihydrofarnesol (R-DHF) and (2E,6E)-farnesol (F-ol)	Acyclic sesquiterpenes	*de novo* biosynthesis/Up-regulation	F-ol and R-DHF has inhibitory effects on *Trichophyton rubrum, Trichophyto mentagrophytes, Microsporum canis* and *Epidermophyton floccosu.*	(Brasch et al. 2014)
F/B	*Candida albicans*	Liquid	*Pseudomonas aeruginosa*	farnesol	Sesquiterpene	Up-regulation	Reduces levels of quinolone and pyocyanin from *Pseudomonas* and inhibits swarming motility in *P. aeruginosa*	(Cugini et al. 2007; McAlester et al. 2008)
F/B	*Candida albicans*	Liquid	*Pseudomonas aeruginosa*	not identified	Not identified	Up-regulation	Inhibits virulence in *Pseudomonas aeruginosa*	(Lopez-Medina et al. 2015)
F/F	*Ceriporiopsis subvermispora*	Solid	*Pleurotus ostreatus*	laccase, manganese peroxidase and lignin-degrading enzymes	Enzymes	Up-regulation	Stimulate wood decay	(Chi et al. 2007)

B/B	Diaminopimelic acid-peptidoglycan producing bacteria	Liquid	*Bacillus subtilis*	Peptidoglycan and disaccharide tripeptides (muropeptide fragments of the cell wall)	Polymers	Up-regulation	Germination stimulation of dormant *Bacillus subtilis* spores	(Shah et al. 2008)
F/B	*Emericella* sp strain CNL-878	Liquid	*Salinispora arenicola* strain CNH-665	emericellamides A and B	Cyclic depsipeptide	Up-regulation	Emericellamide A has moderate antibacterial activity against MRSA and weak cytotoxicity against the human colon carcinoma cell line HCT-116; Emericellamide B was slightly weaker than 1.	(Oh et al. 2007)
F/B	*Chaetomium* sp. (endophytic fungus)	Solid	*Bacillus subtilis*	3- and 4-hydroxybenzoic acid methyl esters, acremonisol A, SB236050, SB238569, isosulochrin, protocatechuic acid methyl ester, shikimeran A, bipherin A, chorismeron, quinomeran and serkydayn	Polyketides, dibenzoic acid, α pyrone and quinolinic alkaloids	Up-regulation/*de novo* biosynthesis	Increase in antibacterial activity and cytotoxicity.	(Akone et al. 2016)
B/B	*Escherichia coli*	Liquid	*Salmonella typhimurium*	indole	Alkaloid	Up-regulation	Increase antibiotic tolerance (carbenicillin and ciprofloxacin) in *Salmonella typhimurium*	(Vega et al. 2013)
F/F	*Eutypa lata*	Solid	*Botryosphaeria obtusa*	O-methylmellein, 4-hydroxy-8-O-methylmellein and 5-hydroxy-8-O-methylmellein	Polyketides (mycoalexins)	Up-regulation/*de novo* biosynthesis	Non-hydroxylated derivative have antifungal and phytotoxic activity	(Glauser et al. 2009)
F/F	*Alternaria tenuissima*	Solid	*Fusarium culmorum* and *Fusarium graminearum*	alternariol, alternariol monomethyl ether, altenuen	Mycotoxins	Down-regulation	Not reported	(Müller et al. 2012)

F/F	*Fusarium culmorum* and *Fusarium graminearum*	Solid	*Alternaria tenuissima*	Deoxynivalenol and zearalenone	Trichothecene and Polyketide	Up-regulation	Deoxynivalenol has antibacterial activity; zearalenone shows cytochrome P450 3A4 inhibition	(Müller et al. 2012)
F/B	*Fusarium oxysporum* MSA35 *(*wild-type strain)	Liquid	*Serratia* sp *and Achromobacter* sp	isocaryophyllene, α-humulene, cyclocaryophyllan-4-ol	Sesquiterpenes	Up-Regulation	Antimicrobial activity of isocaryophyllene.	(Minerdi et al. 2009)
F/F	*Fusarium oxysporum* MSA35 *(*wild-type strain)	Liquid	*F. oxysporum* f. sp. lactucae (pathogenic strain)	isocaryophyllene, α-humulene, cyclocaryophyllan-4-ol	Sesquiterpenes	Up-Regulation	Suppression of virulence in pathogenic *F. oxysporum*	(Minerdi et al. 2009)
F/F	*Fusarium oxysporum* SIN2	Solid	*Sarocladium* cf. strictum SIN29	fusaric acid	Picolinic acid analogue	Up-regulation	Increase *Fusarium oxysporum* virulence	(Bohni et al. 2016)
F/B	*Fusarium pallidoroseum*	Liquid	*Saccharopolyspora erythraea*	N-demethylophiosetin, pallidorosetin A, pallidorosetin B, equisetin, ophiosetin	Decalin-type tetramic acid analogues	*de novo* biosynthesis	No reports	(Whitt et al. 2014)
F/B	*Fusarium tricinctum*	Solid	*Bacillus subtilis*	ateropyrone, enniantin B, B1 and A1; fusaristatin A; macrocarpon C; (−)-citreoisocoumarin; 2-(carboxymethylamino)benzoic acid and (−)-citreoisocoumarinol	Pyrone/Depsipeptides/lipopeptide/Coumarin	Up-regulation/*de novo* biosynthesis	Enniantin B1 and A1 have antibacterial activity against *Bacillus subtilis, Staphylococcus aureus* (MRSA and MSSA), *Streptococcus pneumoniae*, and *Enterococcus faecali;* lateropyrone displayed antibacterial activity against *B. subtilis, S.aureus, S. pneumoniae and E. faecalis*	(Ola et al. 2013)
F/F	*Fusarium tricinctum*	Liquid	*Fusarium begoniae*	subenniatin A-B	Depsipeptides	*de novo* biosynthesis	Inactive in cytotoxic and antibacterial bioassays	(Wang et al. 2013b)

F/F	*Gloeophyllum abietinum*	Liquid and solid	*Heterobasidion annosum*	oosponol, oospoglycol, melledonal A, melledonal C	Polyketides and Sesquiterpenes	Up-regulated	Antibacterial activity	(Sonnenbichler et al. 1994)
B/F	*Gluconobacter* sp.W-315	Liquid	*Neurospora crassa* or *Aspergillus oryzae*	enacyloxin IIa	Polyketide	*de novo* biosynthesis	Antibacterial activity	(Watanabe et al. 1982)
B/B	group A streptococcus (GAS)	Solid	group B streptococcus (GBS)	short hydrophobic peptides (SHP2/3)	Peptides	Up-regulation	Stimulate RovS-mediated gene regulation in GBS	(Cook et al. 2013)
B/B	group B streptococcus (GBS)	Solid	group A streptococcus (GAS)	short hydrophobic peptides (SHP1520)	Peptides	Up-regulation	Modulate Rgg2/3-regulated gene expression in GAS.	(Cook et al. 2013)
B/F	Gut microbiota	Liquid	*Candida albicans*	peptidoglycans	Polymer	Up-regulation	Increases hyphal development of the polymorphic fungal pathogen *C. albicans*	(Xu et al. 2008)
B/B	*Lactobacillus helveticus* M92, *Lactobacillus plantarum* L4, *Enterococcus faecium* L3	Liquid	*Lactococcus lactis* subsp. Lactis LMG 9450	not identified	Bacteriocin	Up-regulation	Antibiotic activity	(Kos et al. 2011; Chanos and Mygind 2016)
B/F	*Lactobacillus kefiranofaciens*	Liquid	*Saccharomyces cerevisiae*	kefiran	Bacteriocin (exopolysaccharide)	Up-regulation	Used as a thickener, stabilizer, emulsifier, fat substitute or gelling agent; also has antitumor activity	(Cheirsilp et al. 2003)
B/F	*Lactobacillus kefiranofaciens*	Liquid	*Saccharomyces cerevisiae*	kefiran	Bacteriocin (exopolysaccharide)	Up-regulation	Used as a thickener, stabilizer, emulsifier, fat substitute or gelling agent; also has antitumor activity	(Tada et al. 2007)
B/B	*Lactobacillus paracasei*	Liquid	*Bacillus subtilis* ATCC 11774	paracin 1.7	Bacteriocin	Up-regulation	Broad-spectrum antibacterial activity	(Ge et al. 2014; Chanos and Mygind 2016)
B/B	*Lactobacillus plantarum* DC400	Liquid	*Lactobacillus sanfranciscensis* DPPMA174 and *Pediococcus pentosaceus* 2XA3	plantaricin A	Bacteriocin (pheromone)	*de novo* biosynthesis	Antibacterial activity	(Di Cagno et al. 2009; Chanos and Mygind 2016)
B/B	*Lactobacillus plantarum* KLDS1.0391	Liquid	*L. helveticus* KLDS1.0737, *Enterococcus faecium* KLDS4.0352, *Lactobacillus reuteri* KLDS1.0737,	plantaricin MG	Bacteriocin	Up-regulation	Antibacterial activity	(Man et al. 2012; Chanos and Mygind 2016)

			Enterococcus faecalis KLDS4.0313					
B/B	*Lactobacillus acidophilus La-5*	Liquid	*Streptococcus thermophilus* STY-3; *Lactobacillus delbrueckii* subsp. bulgaricus LBY-2; *L. delbrueckii* subsp. bulgaricus CECT 400;*L. delbrueckii* subsp. lactis CECT 282 and *Lactobacillus sakei* subsp. sakei CECT 906	lactacin B	Bacteriocin	*de novo* biosynthesis/Up-regulation	Antibacterial activity	(Tabasco et al. 2009; Chanos and Mygind 2016)
B/B	*Lactobacillus acidophilus* N2 (NCFM)	Liquid	*L. delbrueckii, L. helveticus, L. casei, L. fennentum, L. plantarum, L. viridescens, Streptococcus pyogenes, Staphylococcus aureus, Staphylococcus epidermidis, Bacillus cereus, Listeria monocytogenes*	lactacin B	Bacteriocin	*de novo* biosynthesis/Up-regulation	Antibacterial activity	(Barefoot et al. 1994; Chanos and Mygind 2016)
B/B	*Lactobacillus plantarum* J23	Liquid	*L. lactis, L. hilgardii, P. pentosaceus, L. lactis, L. brevis, L. fermentum, L. hilgardii, L. paracasei, L. pentosus, L. plantarum), L. sakei, L. mesenteroides, P. acidilacti, P. Parvulus, P. pentosaceus*	Not identified	Bacteriocin	*de novo* biosynthesis	Antibacterial activity against *Oenococcus oeni*, and a range of *Lactobacillus* and *Pediococcus* species	(Rojo-Bezares et al. 2007; Chanos and Mygind 2016)

B/B	*Lactobacillus plantarum* NC8	Liquid	*Bacillus cereus, Enterococcus faecalis, E. Faecium, L. acidophilus, L. brevis, L. bulgaricus, L. casei, L. fermentum, L. helveticus, L. hilgardii, L. reuteri, L. sake, L. salivarius, L. lactis, L. mesenteroides, Listeria innocua, P. pentosaceous, Staphylococcus carnosus, Streptococcus thermophilus*	plantaricin NC8	Bacteriocin (Class Iib)	Up-regulated	Antibacterial activity	(Maldonado et al. 2004; Chanos and Mygind 2016)
B/B	*Lactococcus lactis* sub sp. Lactis	Liquid	*Yarrowia lipolytica*	nisin	Bacteriocin (Polycyclic peptide)	Up-regulation	Antibacterial activity	(Ariana and Hamedi 2017)
B/F	*Lactococcus lactis* subsp. lactis ATCC 11454	Liquid	*Saccharomyces cerevisiae*	nisin	Bacteriocin (Polycyclic peptide)	Up-regulation	Antibacterial activity	(Liu et al. 2006)
B/F	*Lactococcus lactis* subsp. lactis ATCC11454	Liquid	*Kluyveromyces marxianus*	nisin	Bacteriocin (Polycyclic peptide)	Up-regulation	Antibacterial activity	(Shimizu et al. 1999)
B/B	*Leuconostoc citreum* GJ7	Liquid	*Lactobacillus plantarum* KFRI 464, *Lactobacillus delbrueckii* KFRI 347 and *Leuconostoc mesenteroides* KCTC 1628	kimchicin G7	Bacteriocin	Up-regulation	Antibacterial activity	(Chang et al. 2007; Chanos and Mygind 2016)
F/B	*Libertella* sp	Liquid	Antibiotic-resistant marine a-proteobacterium	libertellenones A–D	Diterpenoids	*de novo* biosynthesis	Antitumoral activity against HCT-116 human adenocarcinoma cell line (libertellenone D is the most potent).	(Oh et al. 2005)
F/F	*Aspergillus* sp. (mangrove epiphytic)	Liquid	*Aspergillus* sp. (mangrove epiphytic)	aspergicin, neoaspergillic acid, ergosterol	Alkaloids and Steroid	*de novo* biosynthesis	Aspergicin, neoaspergillic acid show antibacterial activity against selected Gram bacteria.	(Zhu et al. 2011)
F/F	Mangrove fungi strain No. K38	Liquid	Mangrove fungi strain No. E33	8-hydroxy-3-methyl-9-oxo-9H-xanthene-1-carboxylic acid methyl ether	Xanthone derivative	*de novo* biosynthesis	Antifungal activity against *Gloeasporium musae* and *Peronophthora cichoralearum*	(Li et al. 2011)

F/F	*Phomopsis* sp K38 (mangrove fungi)	Liquid	*Alternaria* sp E33 (mangrove fungi)	cyclo-(L-leucyl-trans-4-hydroxy-L-prolyl-D-leucyl-trans-4-hydroxy-L-proline)	Cyclopeptide (cyclic tetrapeptide)	*de novo* biosynthesis	Antifungal activity against *Gaeumannomyces graminis*, *Rhizoctonia cerealis*, *Helminthosporium sativum* and *Fusarium graminearum*	(Li et al. 2014)
F/F	*Phomopsis* sp K38 (mangrove fungi)	Liquid	*Alternaria* sp E33 (mangrove fungi)	cyclo (D-Pro-L-Tyr-L-Pro-L-Tyr) and cyclo (Gly-L-Phe-L-Pro-L-Tyr)	Cyclopeptide (cyclic tetrapeptide)	*de novo* biosynthesis	Moderate antifungal activity against *C. albicans*, *G. graminis*, *R. cerealis*, *H. sativum* and *F. graminearum*, Cyclo (Gly-L-Phe-L-Pro-L-Tyr) was more active than cyclo (D-Pro-L-Tyr-L-Pro-L-Tyr).	(Huang et al. 2014)
F/F	*Phomopsis* sp K38 (mangrove fungi)	Liquid	*Alternaria* sp E33 (mangrove fungi)	(-)-byssochlamic acid bisdiimid	Diimide derivative	*de novo* biosynthesis	(-)-byssochlamic has weak cytotoxic activity against Hep-2 and HepG2 cells.	(Li et al. 2010)
F/F	*Phomopsis* sp K38 (mangrove fungi)	Liquid	*Alternaria* sp E33 (mangrove fungi)	ethyl 5-ethoxy-2-formyl-3-hydroxy-4-methylbenzoate	Polysubstituted benzaldehyde derivative	*de novo* biosynthesis	Antifungal activity against *Fusarium graminearum*, *Gloeosporium musae*, *Rhizoctonia solani Kuhn* and *Phytophthora soja.*	(Wang et al. 2013a)
F/F	*Marasmius pallescens*	Solid and Liquid	*Marasmiellus troyanus*	laccase and manganese peroxidase activity	Enzymes	Up-regulation	Stimulate wood decay	(Ferreira Gregorio et al. 2006)
B/B	Marine bacteria	Solid	85 other marine isolates	not identified	Not identified	Up-regulation	Antibacterial activity	(Long and Azam 2001)
B/B	*Prochlorococcus* strain MIT9313 and MED4 (marine cyanobacterium)	Liquid	heterotrophic marine bacteria	not identified	Not identified	Up-regulation/*de novo* biosynthesis	Not reported.	(Sher et al. 2011)
B/B	*Micromonospora* spp., *Verrucosispora* spp., *and Solwaraspora* spp. (marine bacteria)	Liquid	*Mycobacterium* sp. WMMA-183 and *Rhodococcus* sp. WMMA-185 (marine mycolic acid-containing bacteria)	not identified	Not identified	Up-regulation/*de novo* biosynthesis	Antibacterial activity	(Adnani et al. 2015)
F/F	Marine-derived mangrove endohytic fungi Strain n. 1924	Liquid	Marine-derived mangrove endohytic fungi Strain n. 3893	cyclo(phe-phe)dipetide and 6-methylsalicylic acid	Peptide and beta-hydroxy acid	*de novo* biosynthesis	6-methylsalicylic acid has biocide activity against pests *Heliothis armigera* Huhner and *Sinergasilus spp.*	(Zhu et al. 2007)

F/F	*Monascus* sp	Solid and Liquid	*Saccharomyces cerevisiae* and *Aspergillus oryzae*	not identified	Pigment	Up-regulation	Overproduction of hydrophobic substances to block enzyme attack (defense mechanism)	(Shin et al. 1998)
F/F/F/F/F	*Ovadendron sulphureoochraceum, Ascochyta pisi, Emericellopsis minima, Cylindrocarpon destructans* and *Fusarium oxysporum*	Liquid	*Ovadendron sulphureoochraceum, Ascochyta pisi, Emericellopsis minima, Cylindrocarpon destructans* and *Fusarium oxysporum*	lateritin	N-methylated peptide (depsipeptide)	*de novo* biosynthesis	Antitumoral activity against human cancer cell lines pancreas BXPC-3, breast MCF-7, CNS SF268, lung NSC H460, colon KM20L2 and prostate DU145); antibacterial activity against *Micrococcus luteus, S. aureus, E. faecalis* and *S. pneumoniae;* antifungal activity against *Candida albicans*	(Pettit et al. 2010)
F/F	*Paraconiothyrium SSM001* (Taxus endophyte)	Liquid	*Alternaria* and *Phomopsis* (bark fungi)	taxol	Diterpenoid	Up-regulation	Anticancer activity	(Soliman and Raizada 2013)
F/F	*Penicillium citrinum*	Liquid	*Beauveria feline*	citrifelins A–B	Citrinin adducts with a unique tetracyclic framework	*de novo* biosynthesis	Citrifelins A and B have antibacterial activity against *E. coli, S. aureus, A. hydrophilia, V. parahemolyticus, V. harveyi, E. tarda, V. alginolyticus*, and *V. anguillarums.*	(Meng et al. 2015)
F/F	*Penicillium fuscum*	Liquid	*Penicillium camembertii/clavigerum*	berkeleylactones A−H, macrolides A26771B, patulin, and citrinin	16-membered-ring macrolides	*de novo* biosynthesis	Berkeleylactone A has potent antibacterial and antifungal activities against four MRSA, *Bacillus anthracis, Streptococcus pyogenes, Candida albicans*, and *Candida glabrata*	(Stierle et al. 2017)
B/F	*Penicillium pinophilum* FKI-5653	Liquid	*Trichoderma harzianum* FKI-5655	secopenicillide C, penicillide, MC-141, pestalasin A and stromemycin	Lactones	Up-regulation	Penicillide acts as antibiotic, plant growth inhibitor, mycotoxin, acyl-CoA-cholesterol acyltransferase inhibitor; anticancer and cytotoxic, non-peptidic oxytocin receptor antagonist and calpain inhibitor.	(Nonaka et al. 2011)

F/B	*Penicillium* sp DT-F29	Solid	*Bacillus* sp B31	verruculogen TR-2; 12-hydroxyverruculogen TR-2; 12b-hydroxy-13a-methoxyverruculogen TR-2; 12b-hydroxy-13a-ethoxyverruculogen TR-2; 12b-hydroxy-13a-butoxyethoxyverruculogen TR-2; 12a-hydroxy-13a-prenylverruculogen TR-2; spirotryprostatin C; cycloprostatin C; 12,13-dihydroxyfumitremorgin C; cyclotryprostatin B;, hydrocycloprostatin A; hydrocycloprostatin B; neofipiperzine C; fumitremorgin B; prenylcycloprostatin B; 25-hydroxyfumitremorgin B; 13-prenyl fumitremorgin B; 12b-hydroxy-13a-butoxyethoxyfumitremorgin B; 12b-hydroxy-13a-methoxyverruculogen B; fumitremorgin A; 26a-hydroxyfumitremorgin A; 25-hydroxyfumitremorgin A, and diprostatin A	2,5-diketopiperazines	*de novo* biosynthesis/Up-regulation	2,5-DKPs show bromodomain-containing protein 4 (BRD4) inhibitory activities; fumitremorgin B is a potential BRD4 inhibitor	(Yu et al. 2017)
F/B	*Pestalotia* sp	Liquid	Antibiotic-resistant marine a-proteobacterium	pestalone	Chlorinated benzophenone	*de novo* biosynthesis	Moderate antitumoral activity against human tumor cell lines; antibacterial activity against MRSA and vancomycin-resistant *Enterococcus faecium*	(Cueto et al. 2001)
F/F	*Phlebia radiata, Phlebia rufa, Coriolus versicolor, Stereum hirsutum, Phanerochaete velutina* and *Hypholoma fasciculare*	Solid	*Phlebia radiata, Phlebia rufa, Coriolus versicolor, Stereum hirsutum, Phanerochaete velutina* and *Hypholoma fasciculare*	phenoloxidase	Enzymes	Up-regulation/*de novo* biosynthesis	Stimulate wood decay	(White and Boddy 1992)
O/ Oomycetes	*Phytophthora nicotianae*	Liquid	*Phytophthora nicotianae*	mating hormones α1 and α2	Hormones	*de novo* biosynthesis	Induction of sexual reproduction in the opposite mating type	(Ojika et al. 2011)
B/F	*Pseudomonas aeruginosa*	Solid	*Candida albicans*	5-methyl-phenazinium-1-carboxylate (5MPCA)	Phenazines derivative (red pigment)	*de novo* biosynthesis	Redox-active antifungal activity	(Gibson et al. 2009)
B/B	*Pseudomonas aeruginosa*	Liquid	*Enterobacter* sp	pyocyanin	Phenazine derivative	*de novo* biosynthesis	Antibacterial activity	(Angell et al. 2006)

B/F	*Pseudomonas aeruginosa*	Liquid	*Candida albicans*	N-3-oxo-dodecanoyl-L-Homoserine lactone (3-oxo-C12HSL)	Fatty acid (DSF)	Up-regulation	Prevents *Candida* morphology switch from yeast to hyphal (filamentous) growth.	(Cugini et al. 2007; McAlester et al. 2008)
B/B or F	*Pseudomonas aeruginosa*	Liquid	*Escherichia coli, Klebsiella pneumoniae, Proteus mirabilis, Streptococcus pyogenes, Bacillus subtilis, Staphylococcus aureus* and *Candida albicans*	cis-2-decenoic acid	a,b Unsaturated fatty acid (DSF)	Up-regulation	Induces the dispersion of biofilm microcolonies on the challenged microbes	(Davies and Marques 2009)
B/B	*Pseudomonas aeruginosa*	Solid	*Staphylococcus aureus*	4-hydroxy-2-heptylquinoline-N-oxide (HQNO)	Quinoline	*de novo* biosynthesis	Suppression of *S. aureus* respiration, protecting *S. aureus* from death by commonly used aminoglycoside antibiotics; Selection of typical *S. aureus* small-colony variants (SCVs), which are stable aminoglycoside resistance and persistence in chronic infections	(Hoffman et al. 2006)
B/B	*Rhodococcus fascians* (multi-antibiotic resistant mutant)	Liquid	*Streptomyces padanus*	rhodostreptomycin A-B	Aminoglycosides	Gene transfer	Antibacterial activity.	(Kurosawa et al. 2008)
F/F	*Schizophyllum commune*	Solid	*Hypholoma fasciculare*	indigo and indirubin	Pigments	Up-regulation	Indirubin is a potent inhibitors of cyclin dependent kinases and has antiviral, antibacterial and antifungal activityes	(Menezes et al. 2015)
B/B	*Actinokineospora* sp. EG49 (sponge-associated Actinomycetes)	Liquid	*Nocardiopsis* sp. RV163 (sponge-associated Actinomycetes)	N-(2-hydroxyphenyl)-acetamide, 1,6-dihydroxyphenazine and 5a,6,11a,12-tetrahydro-5a,11a-dimethyl[1,4]benzoxazino[3,2-b][1,4]benzoxazine	Angucycline, diketopiperazine and β-carboline	Up-regulation/*de novo* biosynthesis	1,6-dihydroxyphenazine has antibacterial activity against *Bacillus sp*. P25 and *Actinokineospora sp*. EG49 and antiprotozoal activity against *Trypanosoma brucei*	(Dashti et al. 2014)
B/B	*Stenotrophomonas maltophilia*	Liquid	*Pseudomonas aeruginosa*	cis-11-methyl-2-dodecenoic acid	a,b Unsaturated fatty acid (DSF)	Up-regulation	Stimulates biofilm filamentation, increases levels of a number of proteins with roles in bacterial stress tolerance and increased tolerance to	(Fouhy et al. 2007; Ryan et al. 2008)

							polymyxins of *P. aeruginosa*	
B/B	*Streptococcus mutans*	Solid/Liquid	*Streptococcus sanguinis*	mutacins	Bacteriocin	Up-regulation	Antibacterial activity against *Streptococcus sanguinis*.	(Kreth et al. 2005)
B/B	*Streptococcus oligofermentas*	Solid/Liquid	*Streptococcus mutans* (major caries-causing pathogen)	hydrogen peroxide (H2O2)	Oxidizing agent	Up-regulation	Antibacterial activity against *Streptococcus mutans*	(Tong et al. 2007)
B/F	*Streptococcus oralis*	Liquid	*Actinomyces naeslundii* T14V	autoinducer 2	Furanosyl borate diester	Up-regulation	Induces biofilm growth in commensal bacteria	(Rickard et al. 2006)
B/B	*Streptococcus sanguinis*	Solid/Liquid	*Streptococcus mutans* (major caries-causing pathogen)	hydrogen peroxide (H2O2)	Oxidizing agent	Up-regulation	Antibacterial activity against *Streptococcus mutans*.	(Kreth et al. 2005)
B/B	*Streptomyces bikiniensis*	Solid	*Salmonella dusseldorf*	streptomycin	Aminoglycoside	Up-regulation	Antibacterial activity against against *Salmonella dusseldorf*.	(Turpin et al. 1992)
B/B	*Streptomyces cinnabarinus* PK209	Liquid	*Alteromonas* sp. KNS-16	lobocompactol	Diterpene	*de novo* biosynthesis	Antifouling activity against macroalgae *Ulva* and *Navicula*; antioxidant and anticancer activity.	(Cho and Kim 2012)
B/B	*Streptomyces cinnamoneus* NBRC 13823	Liquid	*Tsukamurella pulmonis* (mycolic acid-containing bacteria)	arcyriaflavin E	Indolocarbazole alkaloid	*de novo* biosynthesis	Citotoxicity	(Hoshino et al. 2015c)
B/B	*Streptomyces coelicolor*	Solid	*Streptomyces coelicolor*, *Amycolatopsis* sp., *Streptomyces* sp., *Streptomyces viridochromogenes*	four new acyl-desferrioxamines	Hydroxamic acid	Up-regulated/*de novo* biosynthesis	Antibacterial activity	(Traxler et al. 2013)
B/B	*Streptomyces endus* S-522	Liquid	*Tsukamurella pulmonis* S-522 or *Corynebacterium glutamicum* (mycolic acid-containing bacteria)	alchivemycin A	Polycyclic polyketide	*de novo* biosynthesis	Antibacterial activity against *Micrococcus luteus*	(Onaka et al. 2011)

B/B	*Streptomyces lividans* TK23	Liquid	*Tsukamurella pulmonis, T. pseudospumae, T. spumae , T. strandjordii, Corynebacterium glutamicum, C. efficiens, Rhodococcus erythropolis, R. coprophilus, R, wratislaviensis, R, zopfii, Gordonia rubripertincta, G. bronchialis, Nocardia farcinica, N. asteroides, Mycobacterium smegmatis, M. chlorophenolicum, Williamsia muralis* and *Dietzia cinnamea*	actinorhodin and undecylprodigiosin	Benzoisochromanequinone polyketide (red pigment)	*de novo* biosynthesis	Indicator of secondary metabolism	(Onaka et al. 2011)
B/B	*Streptomyces lividans* TK24	Solid	*Salmonella dusseldorf*	not identified	Not identified	Up-regulation	*Streptomyces lividans* increased the survival of *Salmonella dusseldorf*	(Turpin et al. 1992)
F/F	*Streptomyces nodosus* strain 258	Liquid	*C. brumptii, C. parapsilosis, P. lactosa, R. anrantica, R. rubra, Cryptococcus neoformans, C. albicans, C. krusei, C. tropicalis, C. puleherimma* and *C. utilis*	amphotericin B	Polyene	Up-regulation	Antifungal activity	(Yakovleva and Bulgakova 1978)
B/B	*Streptomyces* sp CJ-5	Liquid	*Tsukamurella pulmonis* TP-B0596 (mycolic acid-containing bacteria)	chojalactones A–C	butanolide chojalactones with unusual γ-butyrolactone scaffolds	*de novo* biosynthesis	Chojalactones A and B showed moderate cytotoxicity against P388 cells.	(Hoshino et al. 2015b)
B/B	*Streptomyces* sp NZ-6	Liquid	*Tsukamurella pulmonis* TP-B0596 (mycolic acid-containing bacteria)	niizalactams A–C	Di- and tricyclic macrolactams	*de novo* biosynthesis	Did not show antimicrobial activity or cytotoxicity.	(Hoshino et al. 2015a)

B/B	*Streptomyces sp.* strain B033	Liquid	*Burkholderia vietnamiensis* ATCC BAA-248, *Brucella neotomae* ATCC 23459, *Yersinia pestis* A1122 and *Xanthomonas axonopodis* ATCC 8718 (pathogenic strains of the phylum *Proteobacteria).*	resistomycin	Aminoglycosides	*de novo* biosynthesis	Increase in antibacterial activity.	(Carlson et al. 2015)
B/B	*Streptomyces* sp. Mg1	Solid	*Bacillus subtilis*	surfactin hydrolase	Enzymes	Biotransformat ion	Increase in surfactin hydrolase activity (detoxification).	(Hoefler et al. 2012)
B/B	*Streptomyces* sp. strain PTY087I2	Liquid	*Bacillus subtilis,* MSSA, MRSA and *Pseudomonas aeruginosa (*human pathogens)	granaticin, granatomycin D, and dihydrogranaticin B	Naphthoquinone	Up-regulation	Antibacterial activity against the all gram positive human pathogens used in these experiments	(Sung et al. 2017)
F/B	*Streptomyces* spp.	Liquid	*Bacillus subtilis*	staurosporine	Indolocarbazole alkaloid	Up-regulation	Antibacterial activity and protein kinase inhibition; Germination stimulation of dormant *Bacillus subtilis* spores	(Shah et al. 2008)
B/B	*Streptomyces tenjimariensis*	Liquid	23 different marine bacteria	istamycins A–B	Aminoglycosides	Up-regulation	Antibacterial activity against competitor colonies	(Slattery et al. 2001)
B/B	*Symbiobacterium thermophilum*	Liquid	*Bacillus* sp	2,2-bis(3 -indolyl)indoxyl and 1,1-bis(3 -indolyl)ethane	Indole	*de novo* biosynthesis	Antibacterial activities against *Staphylococcus aureus* and *Bacillus subtilis*	(Watsuji et al. 2007)
F/F	*Trichoderma harzianum* SIWT 25	Solid	*Serpula lacrymans* and *Coniophora puteuna*	tyrosinase	Enzymes	Up-regulation	Stimulate wood decay	(Score et al. 1997)
F/F	*Trametes versicolor*	Liquid	*Pleurotus ostreatus*	laccase	Enzymes	Up-regulation	Stimulate wood decay	(Baldrian 2004)

F/F	*Trametes versicolor*	Solid	*Stereum gausapatum, Daldinia concentrica, Bjerkandera adusta, Fomes fomentarius* and *Hypholoma fasciculare* (five other wood decay fungi)	laccase and manganese-repressed peroxidase (MRP)	Enzymes	Up-regulation	Stimulate wood decay. Laccase acts to oxidise a variety of phenolic compounds and aromatic diamines; peroxidases utilise hydrogen peroxide (H_2O_2) to catalyse the oxidation of a range of organic and inorganic compounds.	(Hiscox et al. 2010)
F/F	*Trametes versicolor*	Solid	*Ganoderma applanatum*	(N-(4-methoxyphenyl)formamide 2-O-β-D-xyloside and N-(4-methoxyphenyl)formamide 2-O-beta-D-xylobioside), 3-phenyllactic acid, gluconic acid and orsellinic acid	Xylosides and Carboxylic acids	Up-regulation/*de novo* biosynthesis	N-(4-methoxyphenyl)formamide 2-O-beta-D-xylobioside) has potential to enhance the cell viability of human immortalized bronchial epithelial cell line of Beas-2B	(Yao et al. 2016)
F/F	*Trametes versicolor* and *Pleurotus ostreatus*	Liquid	*Trichoderma harzianum*, bacteria and yeasts (soil microbes)	laccase	Enzymes	Up-regulation	Stimulate wood decay	(Baldrian 2004)
B/B	*Escherichia coli* P5v (transformed bacteria engineered to provide DHS precursor)	Liquid	*Escherichia coli* (transformed strains engineered with 3AB synthase and the DHS transporter to provide 3AB)	3-amino-benzoic acid (3AB)	Organic Acid	Up-regulation	3AB is an important building block molecule for biosynthesis of several natural products	(Zhang and Stephanopoulos 2016)
F/P	*Trichoderma atroviride*	Liquid	*Arabidopsis thaliana* seedlings	Indole–acetic acid-related indoles	Indoles	Up-regulation	Stimulatory effect on plant growth	(Salas-Marina et al. 2011)
F/F	*Trichoderma harzianum*	Solid	*Moniliophthora roreri*	T39 butenolide, harzianolide, sorbicillinol	Lactones	*de novo* biosynthesis	T39 butenolide and harzianolide have antifungal activity against phythopathoges.	(Tata et al. 2015)
F/F	*Trichoderma* sp	Liquid	*Lentinula edodes*	β-mannosidase and laccase	Enzymes	Up-regulation	Stimulate wood decay. Lowering of Mn-dependent peroxidase activity	(Savoie and Mata 1999)
F/F	Unidentified Fungi (strain Nos. 1924)	Liquid	Unidentified Fungi (strain Nos. 3893)	marinamide (4-(2-pyrrolyl)-1-isoquinolone-3-carboxylic acid) and methyl ester marinamide (methyl 4-(2-pyrrolyl)-1-isoquinolone-3-carboxylate)	Alkaloids	*de novo* biosynthesis	Antibacterial activity against *E. coli*, *Pseudomonas pyocyanea*, and *S. aureus;* Antitumoral activity against tumor cell lines	(Zhu et al. 2007; Zhu et al. 2013)

B/B	Unidentified surface-associated marine bacteria	Liquid	*Staphylococcus aureus, Pseudomonas aeruginosa* or *Escherichia coli* (human pathogens)	not identified	Not identified	Up-regulation	Antibacterial activity	(Mearns-Spragg et al. 1998)
F/F	*Trametes versicolor*	Liquid	*Trichoderma harzianum (*soil fungus and biocontrol organism)	laccase and cellobiase	Enzymes	Up-regulation	Stimulate wood decay	(Freitag and Morrell 1992)
B/F	*Xanthomonas campestris* pv. campestris	Liquid	*Candida albicans* and *Xanthomonas campestris pv. Campestris*	cis -11-methyl-2-dodecenoic acid	a,b Unsaturated fatty acid (DSF)	Up-regulation	Regulates virulence in *X. Campestris;* Regulates morphological transition and virulence by *C. albicans*	(Wang et al. 2004)
B/B	*Xylella fastidiosa*	Liquid	*Methylobacterium mesophilicum*	not identified	Not identified	Up-regulation	*X. fastidiosa* down-regulate genes related to growth and up-regulate genes related to energy production, stress, transport, and motility,	(Dourado et al. 2015)
F/B	*Fusarium oxysporum* f. sp. radicis-lycopersici (soil-borne fungus)	Liquid	*Pseudomonas chlororaphis* strain PCL1391	fusaric acid	Polyketide	Up-regulation	Represses the production of signal N-hexanoyl-L-homoserine lactone (C6-HSL).and phenazine-1-carboxamide	(van Rij et al. 2005)
F/B	*Fusarium oxysporum*	Liquid	*Pseudomonas fluorescens* CHA0 (biocontrol strain)	fusaric acid	Polyketide	Up-regulation	Suppress 2,4-diacetylphloroglucinol (2,4-DAPG) production in strain CHA0	(Notz et al. 2002)
F/B	*Fusarium oxysporum*	Liquid	*Pseudomonas fluorescens*	Enzyme	Enzymes	Up-regulation	Degrade 2,4-DAPG into less toxic derivatives monoacetyl-phloroglucinol and phloroglucinol	(Schouten et al. 2004)
F/F	biocontrol agent *Trichoderma harzianum* M10	Liquid	endophyte *Talaromyces pinophilus* F36CF	harziaphilic acid	tetramic acid derivative	*de novo* biosynthesis	Harziaphilic acids did not affect viability of colorectal cancer and healthy colonic epithelial cells, but selectively reduced cancer cell proliferation.	(Vinale et al. 2017)
F/F	biocontrol agent *Trichoderma harzianum* M10	Liquid	endophyte *Talaromyces pinophilus* F36CF	ferricrocin, coprogen B and dimerumic acid	siderophores	Up-regulation	-	(Vinale et al. 2017)

F/F	biocontrol agent *Trichoderma harzianum* M10	Liquid	endophyte *Talaromyces pinophilus* F36CF	3-O-methylfunicone and herquline B	γ-pyrone	Down-regulation	-	(Vinale et al. 2017)
F/B	*Aspergillus niger*	Liquid	*Streptomyces coelicolor*	cyclo(Phe-Phe) and 2-hydroxyphenylacetic acid	cyclic dipeptide	Up-regulation	-	(Wu et al. 2015)
F/B	*Aspergillus fumigatus*	Liquid	*Streptomyces rapamycinicus*	fumigermin	polyketide	*de novo* biosynthesis	Fumigermin inhibits germination of spores of the inducing S. rapamycinicus	(Stroe et al. 2020)
B/F	marine *Streptomyces*	Liquid	*Bacillus* strains	dentigerumycin E	cyclic hexapeptide incorporating three piperazic acids	*de novo* biosynthesis	Dentigerumycin E exhibited antiproliferative and antimetastatic activities against human cancer cells	(Shin et al. 2018)
F/B	*Fusarium tricinctum*	Solid	*Streptomyces lividans*	fusatricinones A–D (1), dihydrolateropyrone (2), zearalenone, (−)-citreoisocoumarin, macrocarpon C and 7-hydroxy-2-(2-hydroxypropyl)-5-methylchromone	naphthoquinone dimers (1), lateropyrone derivative (2)	*de novo* biosynthesis	-	(Moussa et al. 2019)
F/B	*Fusarium tricinctum*	Solid	*Streptomyces lividans*	lateropyrone, the enniatins B, B1 and A1, and fusaristatin A	Depsipeptides and lipopeptide	Up-regulation	-	(Moussa et al. 2019)
B/F	*Pseudomonas aeruginosa*	Solid	*Fusarium tricinctum*	2-heptyl- 4-hydroxy-quinolone (HHQ), phenazine-1-carboxylic acid (PCA) and phenazine-1-carboxamide (PCN)	quorum sensing molecule and phenazine alkaloids	*de novo* biosynthesis	-	(Moussa et al. 2020)
F/F	*Ustilago maydis*	solid	*Fusarium verticillioides*	Over 20 chromatographic peaks	-	*de novo* biosynthesis	*F. verticillioides* is a strong antagonist of *U. maydis* as its presence leads to large reductions in *U. maydis* biomass.	(Estrada et al. 2011)

F/F	*Stereum hirsutum*	Solid	*Coprinus micaceus* and *Coprinus disseminatus*	-	-	Up-regulation	*S. hirsutum* mycelial fronts always overgrew those of *C. micaceus*, there were down-regulations of metabolites in the interaction zone, compared to monocultures of both *S. hirsutum* and *C. micaceus.*	(Peiris et al. 2008)
F/F	*Hypholoma fasciculare*	Solid	*Resinicium bicolor*	benzoic acid methyl ester, a benzyl alcohol, a quinolinium type compound and other volatiles	sesquiterpenes	*de novo* biosynthesis	two wood decay basidiomycete fungi	(Hynes et al. 2007)
F/F	*Rhodotorula glutinis* DBVPG 3853	Liquid	*Debaryomyces castellii DBVPG 3503*	β-carotene, torulene, torularhodin	carotenoid pigments	Up-regulation	-	(Buzzini 2001)
F/B	*Penicillium notatum*	Liquid	Cyanobacterium *Scytonema ocelutum*	*tolytoxin*	Phytoalexin	*de novo* biosynthesis	Antifungal	(Patterson and Bolis 1997)
B/F	*Streptomyces sp.* US80	Liquid	heat-killed fungi	irumamycin, X-14952 B and 17-hydroxy-venturicidin A	-	Up-regulation	Antifungal	(Fourati-Ben Fguira et al. 2008)
B/B	*Streptomyces sp. RKBH-B178*	Liquid	autoclaved *Mycobacterium smegmatis*	hydrazidomycin D	hydrazide	*de novo* biosynthesis	-	(Liang et al. 2019)
B/B	*Streptomyces sp. RKBH-B178*	Liquid	autoclaved *Pseudomonas aeruginosa*	Glucuronidated-PQS	glucuronidated analog of the pseudomonas quinolone signal (PQS)	*de novo* biosynthesis	-	(Liang et al. 2019)
B/B	*Bacillus cereus*, *Flavobacterium johnsoniae*, and *Pseudomonas koreensis*	Liquid	*Bacillus cereus*, *Flavobacterium johnsoniae*, and *Pseudomonas koreensis*	zwittermicin, petrobactin, derivatives of lokisin	sidephores	Up-regulation	Antibiotic	(Chevrette et al. 2022)

B/B	*Streptomyces hygroscopicus* HOK021	Liquid	*Tsukamurella pulmonis* TP-B0596	harundomycin A	conjugate between FabF inhibitor and catechol-type siderophore	*de novo* biosynthesis	Bifunctional Antibacterial Conjugate	(Asamizu et al. 2022)
F/F	*Aspergillus carneus* KMM 4638	Liquid	*Beauveria felina* (=*Isaria felina*) KMM 4639	asperflavinoids B, D and E	drimane-Type Sesquiterpenes	*de novo* biosynthesis	Cytotoxic	(Zhuravleva et al. 2022)
F/B	*Aspergillus nidulans*	Liquid	*Streptomycetes*	anhydrosepedonin (**1**) and antibiotic C (**2**)	iron-chelating tropolones and tripyrnidone	*de novo* biosynthesis and up-regulation	antibacterial	(Gerke et al. 2022)
F/F	*Alternaria*	Liquid	*Trichoderma atroviride*	alternariol	benzochromenone	Up-regulation	mycotoxin	(Tian et al. 2023)
F/F	Trichoderma *atr oviride*	Liquid	*Alternaria*	Hydroxylation of altern ariol (AOH-OH)	benzochromenone	Biotransform ation	-	(Tian et al. 2023)
B/F	*Streptomyces* sp 13F051	Liquid	*Leohumicola minima* 15S071	ulleungdolin	polyketide–peptide hybrid	*de novo* biosynthesis	-	(Hwang et al. 2022)
F/F	*Botrytis cinerea*	Liquid	*Pichia fermentans* (two strains), *Issatchenkia terricola* and *Wicker hamomyces anomalus.*	46 differentially secreted metabolites, including *trans*-cinnamic acid and the indole-3-carboxaldehyde	Phenylpropanoid and alkaloid	Up-regulation	Antifungal for biocontrol	(Fernandez-San Millan et al. 2022)
F/F	*Aspergillus oryz ae*	Solid	Zygosaccharomyces *rouxii*	l-Arginine and l-Glutamate increase and l-Isoleucine decreased	Aminoacids	Regulation	- fungal breakage rate increased<br>- enzyme activity decreased	(Liu et al. 2022b)
F/F	*Trichoderma* strains	Liquid	*T richoderma* strains	Unkown	Unkown	Regulation	- synergistically promote plant growth<br>- antagonistic activity against pathogens	(Liu et al. 2022a)
F/F	*Aspergillus nidulans*	Liquid	*Aspergillus fumigatus*	diphenyl ethers	polyketides	Up-regulation	Antibacterial	(Ninomiya et al. 2022)

F/F	*Aspergillus fumigatus*		*Aspergillus nidulans*	Unkown	siderophore	Up-regulation	-	(Ninomiya et al. 2022)
F/F	*Fusarium oxysporum* AB2	Liquid	*Epicoccum nigrum* TORT	Beauvericin	Depsipeptides	Up-regulation	insecticidal and antibacterial activities	(Vásquez-Bonilla et al. 2022)
F/F	*Azospirillum oryzae* NBT506	Liquid	*Bacillus velezensis* UTB96	Surfactin and indole-3-acetic acid (AA)	surfactin production, indole-3-acetic acid (IAA) production, phosphate solubilization and enzyme activities	Up-regulation	plant growth-promoting bacteria (PGPB)	(Bagheri et al. 2022)
F/F	*Aspergillus oryz ae*	Liquid	Zygosaccharomyces *rouxii*	Unkown	Unkown	Up-regulation	Antibacterial	(Liu et al. 2022c)
F/F	deep-sea-derived fungi *Penicillium crustosum* PRB-2	Solid	*Penicillium fellutanum* HDN14-323	penifellutins A (1), B (2), penifellutins C (3) and D (4),	Polyketides	*de novo* biosynthesis	-	(Yu et al. 2022)
F/B	*Aspergillus flavipes*	Solid	*Bacillus subtilis*	Taxol	-	Up-regulation	`Chemotherapy	(El-Sayed et al. 2021)
F/F	*Penicillium fuscum*	Solid	*P. camembertii/clavigerum*	berkeleypenostatins A–G (1–7), berkeleylactones A–H, the known macrolide A26771B, citrinin, and patulin	macrolide	*de novo* biosynthesis and Up-regulation	Anticancer	(Stierle et al. 2021)
B/F	*Streptomyces lunalinharesii*	Solid	Phytopathogen *Rhizoctonia solani*	desferrioxamine E and anisomycin	siderophores	Up-regulation	-	(Maimone et al. 2021)
B/F	wild-type lactic acid bacteria	Liquid	auxotrophic *Saccharomyces cerevisiae*	riboflavin and folate	B-group vitamins	Up-regulation	-	(Konstantinidis et al. 2021)
F/F	Marine *Aspergillus terreus* C23-3	Liquid	Marine *Aspergillus. unguis* DLEP2008001	unguisin A, lovastatin, and nidulin and others	Multiple	Up-regulation	Antimicrobial	(Wang et al. 2022)

F/F	*Irpex lacteus*	Solid	*Nigrospora oryzae*	irperide	butenolide	*de novo* biosynthesis	Antifungal	(Wu et al. 2022)
F/F	*Alternaria, Botryosphaeria, Phoma* and *Talaromyces*	Solid	*Fusarium pseudograminearum* (Fp)	Unkown	Unkown	Unkown	Antifungal	(Zhao et al. 2022)
F/B	*Aspergillus sydowii*	Liquid	*Bacillus subtilis*	s (−)- (7S)- 10-hydroxysydonic acid, serine sydonate and macrolactin U'	-	Up-regulation	Antibacterial against Staphylococcus aureus	(Sun et al. 2022)
B/B	*Enterococcus faecium* 135	Liquid	*Ligilactobacillus salivarius* and *Limosilactobacillus reuteri*	bacteriocin-like inhibitory substances (BLIS)	Bacteriocin	Up-regulation	Antibacterial	(Piazentin et al. 2022)
B/B	*Streptomyces sp*	Liquid	*Achromobacter sp*	Ligiamycins A and B	Decalin-Amino-Maleimides	Up-regulation	-	(Lim et al. 2022)
F/F	*Cosmospora sp.*	Solid	Phytopathogen *Magnaporthe oryzae*	soudanones A, E, D (1-3) and their two new derivatives, soudanones H-I (4-5), the known isochromans, pseudoanguillosporins A and B (6, 7), naphtho-γ-pyrones, cephalochromin and ustilaginoidin G (8, 9), and ergosterol (10).	Isochromanones	*de novo* biosynthesis and Up-regulation	-	(Oppong-Danquah et al. 2022)
B/B	*Streptomyces sp*	Solid	*Pandoraea sp.*	Gwanakosides A and B	6-Deoxy-α-l-talopyranose-Bearing Aromatic Metabolites	*de novo* biosynthesis	Antibacterial	(Huynh et al. 2022)
B/B	Soil *Bacillus velezensis*	Solid	*Pseudomonas*	surfactin	lipopeptides	Up-regulation	- enhance motility<br>- chemical trap to reduce the toxicity	(Andrić, S et al. 2021)

F/F	*Trametes coccinea* (F3)	Solid	*Leiotrametes lactinea* (F9) and *T. versicolor* (F1)	Melanin and laccase	Group of multicopper oxidases involved in detoxification of toxic compounds	Up-regulation	-	(Dullah et al. 2021)
F/F	*Talaromyces calidicanius*	Solid	*Aspergillus chevalieri*	Unkown	Unkown	Up-regulation	inhibiting the enzyme acetylcholinesterase	(de Oliveira et al. 2022)
F/B	*Aspergillus nidulans*	Liquid	*Streptomyces iranensis*	orsellinic acid, lecanoric acid, F-9775A and F-9775B	Archetypal polyketides and pentacyclic triterpenes	*de novo* biosynthesis	Arginine-derived polyketides serve as the bacterial signals	(Krespach et al. 2022)
B/F	*Paenibacillus peoriae strain M48F*	Solid	*Pleurotus ostreatus HK35* and *Pleurotus eryngii DSMZ 8264*	5-diisopropylpyrazine	VOC	Up-regulation	enhance mycelial growth	(Orban et al. 2023)
F/B	*Pleurotus ostreatus HK35* and *Pleurotus eryngii DSMZ 8264*	Solid	*Paenibacillus peoriae strain M48F*	β-bisabolene	sesquiterpenes	Up-regulation	fungal defense reaction	(Orban et al. 2023)
A/F	marine dinoflagellate *Prorocentrum lima*	Liquid	*Aspergillus pseudoglaucus*	okadaic acid and dinophysistoxin 1	Up-regulation	Up-regulation	Toxines	(Berry et al. 2022)

References

Adnani N, Vazquez-Rivera E, Adibhatla SN, Ellis GA, Braun DR, Bugni TS (2015) Investigation of interspecies interactions within marine micromonosporaceae using an improved co-culture approach. Marine Drugs 13:6082–6098. https://doi.org/10.3390/md13106082

Akone SH, Mándi A, Kurtán T, Hartmann R, Lin W, Daletos G, Proksch P (2016) Inducing secondary metabolite production by the endophytic fungus Chaetomium sp . through fungal e bacterial co-culture and epigenetic modi fi cation. Tetrahedron 72:6340–6347. https://doi.org/10.1016/j.tet.2016.08.022

Andrić, S, Meyer T, Rigolet A, Prigent-Combaret C, Höfte M, Balleux G, Steels S, Hoff G, De Mot R, McCann A, De Pauw E, Arias AA, Ongena M (2021) Lipopeptide Interplay Mediates Molecular Interactions between Soil Bacilli and Pseudomonads. Microbiology Spectrum 9:e02038-21. https://doi.org/10.1128/spectrum.02038-21

Angell S, Bench BJ, Williams H, Watanabe CMH (2006) Pyocyanin Isolated from a Marine Microbial Population: Synergistic Production between Two Distinct Bacterial Species and Mode of Action. Chemistry and Biology 13:1349–1359. https://doi.org/10.1016/j.chembiol.2006.10.012

Araújo WL, Creason AL, Mano ET, Camargo-Neves AA, Minami SN, Chang JH, Loper JE (2016) Genome Sequencing and Transposon Mutagenesis of Burkholderia seminalis TC3.4.2R3 Identify Genes Contributing to Suppression of Orchid Necrosis Caused by B. gladioli. Molecular plant-microbe interactions : MPMI 29:MPMI02160047R. https://doi.org/10.1094/MPMI-02-16-0047-R

Ariana M, Hamedi J (2017) Enhanced production of nisin by co-culture of Lactococcus lactis sub sp. lactis and Yarrowia lipolytica in molasses based medium. Journal of Biotechnology 256:21–26. https://doi.org/10.1016/j.jbiotec.2017.07.009

Asamizu S, Pramana AAC, Kawai S, Arakawa Y, Onaka H (2022) Comparative Metabolomics Reveals a Bifunctional Antibacterial Conjugate from Combined-Culture of *Streptomyces hygroscopicus* HOK021 and *Tsukamurella pulmonis* TP-B0596. ACS Chem Biol 17:2664–2672. https://doi.org/10.1021/acschembio.2c00585

Bagheri N, Ahmadzadeh M, Mariotte P, Jouzani GS (2022) Behavior and interactions of the plant growth-promoting bacteria Azospirillum oryzae NBT506 and Bacillus velezensis UTB96 in a co-culture system. World Journal of Microbiology and Biotechnology 38:101. https://doi.org/10.1007/s11274-022-03283-8

Baldrian P (2004) Increase of laccase activity during interspecific interactions of white-rot fungi. FEMS Microbiology Ecology 50:245–253. https://doi.org/10.1016/j.femsec.2004.07.005

Barefoot SF, Chen Y, Hughes TA, Bodine AB, Shearer MY, Hughes MD (1994) Identification and Purification of a Protein That Induces Production of the Lactobacillus acidophilus Bacteriocin Lactacin Bt. Applied and Environmental Microbiology 60:3522–3528

Benitez L, Correa A, Daroit D, Brandelli A (2011) Antimicrobial activity of Bacillus amyloliquefaciens LBM 5006 is enhanced in the presence of Escherichia coli. Current Microbiology 62:1017–1022. https://doi.org/10.1007/s00284-010-9814-z

Berleman JE, Chumley T, Cheung P, Kirby JR (2006) Rippling is a predatory behavior in Myxococcus xanthus. Journal of Bacteriology 188:5888–5895. https://doi.org/10.1128/JB.00559-06

Berry O, Briand E, Bagot A, Chaigné M, Meslet-Cladière L, Wang J, Grovel O, Jansen JJ, Ruiz N, Robiou du Pont T, Pouchus YF, Hess P, Bertrand S (2022) Deciphering interactions between the marine dinoflagellate Prorocentrum lima and the fungus Aspergillus pseudoglaucus. Environmental Microbiology n/a. https://doi.org/10.1111/1462-2920.16271

Bertrand S, Schumpp O, Bohni N, Monod M, Gindro K, Wolfender J-L (2013) De novo production of metabolites by fungal co-culture of Trichophyton rubrum and Bionectria ochroleuca. Journal of Natural Products 76:1157–1165. https://doi.org/10.1021/np400258f

Bohni N, Hofstetter V, Gindro K, Buyck B, Schumpp O, Bertrand S, Monod M, Wolfender J-L (2016) Production of fusaric acid by Fusarium spp. in pure culture and in solid medium co-cultures. Molecules 21:370–386. https://doi.org/10.3390/molecules21030370

Boon C, Deng Y, Wang LH, He Y, Xu JL, Fan Y, Pan SQ, Zhang LH (2008) A novel DSF-like signal from Burkholderia cenocepacia interferes with Candida albicans morphological transition. ISME Journal 2:27–36. https://doi.org/10.1038/ismej.2007.76

Brasch J, Horter F, Fritsch D, Beck-Jendroschek V, Troger A, Francke W (2014) Acyclic sesquiterpenes released by Candida albicans inhibit growth of dermatophytes. Medical Mycology 52:48–55. https://doi.org/10.3109/13693786.2013.814174

Butcher RA, Schroeder FC, Fischbach MA, Straight PD, Kolter R, Walsh CT, Clardy J (2007) The identification of bacillaene, the product of the PksX megacomplex in Bacillus subtilis. Proceedings of the National Academy of Sciences 104:1506–1509. https://doi.org/10.1073/pnas.0610503104

Buzzini P (2001) Batch and fed-batch carotenoid production by Rhodotorula glutinis - Debaryomyces castellii co-cultures in corn syrup. Journal of Applied Microbiology 90:843–847. https://doi.org/10.1046/j.1365-2672.2001.01319.x

Carlson S, Tanouye U, Omarsdottir S, Murphy BT (2015) Phylum-specific regulation of resistomycin production in a streptomyces sp. via microbial coculture. Journal of Natural Products 78:381–387. https://doi.org/10.1021/np500767u

Chagas FO, Dias LG, Pupo MT (2013) A Mixed Culture of Endophytic Fungi Increases Production of Antifungal Polyketides. Journal of Chemical Ecology 39:1335–1342. https://doi.org/10.1007/s10886-013-0351-7

Chang JY, Lee HJ, Chang HC (2007) Identification of the agent from Lactobacillus plantarum KFRI464 that enhances bacteriocin production by Leuconostoc citreum GJ7. Journal of Applied Microbiology 103:2504–2515. https://doi.org/10.1111/j.1365-2672.2007.03543.x

Chanos P, Mygind T (2016) Co-culture-inducible bacteriocin production in lactic acid bacteria. Applied Microbiology and Biotechnology 100:4297–4308. https://doi.org/10.1007/s00253-016-7486-8

Cheirsilp B, Shimizu H, Shioya S (2003) Enhanced kefiran production by mixed culture of and. Journal of Biotechnology 100:43–53. https://doi.org/10.1016/S0168-1656(02)00228-6

Chevrette MG, Thomas CS, Hurley A, Rosario-Meléndez N, Sankaran K, Tu Y, Hall A, Magesh S, Handelsman J (2022) Microbiome composition modulates secondary metabolism in a multispecies bacterial community. Proc Natl Acad Sci USA 119:e2212930119. https://doi.org/10.1073/pnas.2212930119

Chi Y, Hatakka A, Maijala P (2007) Can co-culturing of two white-rot fungi increase lignin degradation and the production of lignin-degrading enzymes? International Biodeterioration and Biodegradation 59:32–39. https://doi.org/10.1016/j.ibiod.2006.06.025

Cho JY, Kim MS (2012) Induction of Antifouling Diterpene Production by Streptomyces cinnabarinus PK209 in Co-Culture with Marine-Derived Alteromonas sp. KNS-16. Bioscience, Biotechnology, and Biochemistry 76:1849–1854. https://doi.org/10.1271/bbb.120221

Cook LC, LaSarre B, Federle MJ (2013) Interspecies communication among commensal and pathogenic streptococci. mBio 4:1–11. https://doi.org/10.1128/mBio.00382-13

Cueto M, Jensen PR, Kauffman C, Fenical W, Lobkovsky E, Clardy J (2001) Pestalone, a New Antibiotic Produced by a Marine Fungus in Response to Bacterial Challenge. Journal of Natural Products 64:1444–1446

Cugini C, Calfee MW, Farrow JM, Morales DK, Pesci EC, Hogan DA (2007) Farnesol, a common sesquiterpene, inhibits PQS production in Pseudomonas aeruginosa. Molecular Microbiology 65:896–906. https://doi.org/10.1111/j.1365-2958.2007.05840.x

Dashti Y, Grkovic T, Abdelmohsen UR, Hentschel U, Quinn RJ (2014) Production of induced secondary metabolites by a co-culture of sponge-associated actinomycetes, Actinokineospora sp. EG49 and Nocardiopsis sp. RV163. Marine Drugs 12:3046–3059. https://doi.org/10.3390/md12053046

Davies DG, Marques CNH (2009) A fatty acid messenger is responsible for inducing dispersion in microbial biofilms. Journal of Bacteriology 191:1393–1403. https://doi.org/10.1128/JB.01214-08

de Oliveira GP, Barreto DLC, Ramalho Silva M, Augusti R, Evódio Marriel I, Gomes de Paula Lana U, Takahashi e A (2022) Biotic stress caused by in vitro co-inoculation enhances the expression of acetylcholinesterase inhibitors by fungi. Natural Product Research 36:4266–4270. https://doi.org/10.1080/14786419.2021.1975701

Degenkolb T, Heinze S, Schlegel B, Strobel G, Gräfe U (2002) Formation of new lipoaminopeptides, acremostatins A, B, and C, by co-cultivation of Acremonium sp. Tbp-5 and Mycogone rosea DSM 12973. Bioscience, Biotechnology, and Biochemistry 66:883–886. https://doi.org/10.1271/bbb.66.883

Di Cagno R, De Angelis M, Coda R, Minervini F, Gobbetti M (2009) Molecular adaptation of sourdough Lactobacillus plantarum DC400 under co-cultivation with other lactobacilli. Research in Microbiology 160:358–366. https://doi.org/10.1016/j.resmic.2009.04.006

Dourado MN, Santos DS, Nunes LR, Costa de Oliveira RLB, de Oliveira M V, Araújo WL (2015) Differential gene expression in Xylella fastidiosa 9a5c during co-cultivation with the endophytic bacterium Methylobacterium mesophilicum SR1.6/6. Journal of Basic Microbiology 55:1357–1366. https://doi.org/10.1002/jobm.201400916

Dullah S, Hazarika DJ, Goswami G, Borgohain T, Ghosh A, Barooah M, Bhattacharyya A, Boro RC (2021) Melanin production and laccase mediated oxidative stress alleviation during fungal-fungal interaction among basidiomycete fungi. IMA Fungus 12:33. https://doi.org/10.1186/s43008-021-00082-y

Dusane DH, Matkar P, Venugopalan VP, Kumar AR, Zinjarde SS (2011) Cross-species induction of antimicrobial compounds, biosurfactants and quorum-sensing inhibitors in tropical marine epibiotic bacteria by pathogens and biofouling microorganisms. Current Microbiology 62:974–980. https://doi.org/10.1007/s00284-010-9812-1

Ebrahim W, El-Neketi M, Lewald LI, Orfali RS, Lin W, Rehberg N, Kalscheuer R, Daletos G, Proksch P (2016) Metabolites from the Fungal Endophyte Aspergillus austroafricanus in Axenic Culture and in Fungal-Bacterial Mixed Cultures. Journal of Natural Products 79:914–922. https://doi.org/10.1021/acs.jnatprod.5b00975

El-Sayed ASA, Shindia AA, AbouZeid A, Koura A, Hassanein SE, Ahmed RM (2021) Triggering the biosynthetic machinery of Taxol by Aspergillus flavipes via cocultivation with Bacillus subtilis: proteomic analyses emphasize the chromatin remodeling upon fungal-bacterial interaction. Environmental Science and Pollution Research 28:39866–39881. https://doi.org/10.1007/s11356-021-13533-1

Estrada AER, Hegeman A, Kistler HC, May G (2011) In vitro interactions between Fusarium verticillioides and Ustilago maydis through real-time PCR and metabolic profiling. Fungal Genetics and Biology 48:874–885. https://doi.org/10.1016/j.fgb.2011.06.006

Fernandez-San Millan A, Gamir J, Larraya L, Farran I, Veramendi J (2022) Towards understanding of fungal biocontrol mechanisms of different yeasts antagonistic to Botrytis cinerea through exometabolomic analysis. Biological Control 174:105033. https://doi.org/10.1016/j.biocontrol.2022.105033

Ferreira Gregorio AP, Da Silva IR, Sedarati MR, Hedger JN (2006) Changes in production of lignin degrading enzymes during interactions between mycelia of the tropical decomposer basidiomycetes Marasmiellus troyanus and Marasmius pallescens. Mycological Research 110:161–168. https://doi.org/10.1016/j.mycres.2005.10.002

Fouhy Y, Scanlon K, Schouest K, Spillane C, Crossman L, Avison MB, Ryan RP, Dow JM (2007) Diffusible signal factor-dependent cell-cell signaling and virulence in the nosocomial pathogen Stenotrophomonas maltophilia. Journal of Bacteriology 189:4964–4968. https://doi.org/10.1128/JB.00310-07

Fourati-Ben Fguira L, Smaoui S, Karray-Rebai I, Bejar S, Mellouli L (2008) The antifungal activity of the terrestrial Streptomyces US80 strain is induced by heat-killed fungi. Biotechnology journal 3:1058–1066. https://doi.org/10.1002/biot.200700155

Freitag M, Morrell JJ (1992) Changes in selected enzyme activities during growth of pure and mixed cultures of the white-rot decay fungus Trametes versicolor and the potential biocontrol fungus Trichoderma harzianum. Canadian Journal of Microbiology 38:317–23. https://doi.org/10.1139/m92-053

Ge J, Fang B, Wang Y, Song G, Ping W (2014) Bacillus subtilis enhances production of Paracin1.7, a bacteriocin produced by Lactobacillus paracasei HD1-7, isolated from Chinese fermented cabbage. Annals of Microbiology 64:1735–1743. https://doi.org/10.1007/s13213-014-0817-z

Gerke J, Köhler AM, Wennrich J-P, Große V, Shao L, Heinrich AK, Bode HB, Chen W, Surup F, Braus GH (2022) Biosynthesis of Antibacterial Iron-Chelating Tropolones in Aspergillus nidulans as Response to Glycopeptide-Producing Streptomycetes. Frontiers in Fungal Biology 2

Gibson J, Sood A, Hogan DA (2009) Pseudomonas aeruginosa-Candida albicans interactions: Localization and fungal toxicity of a phenazine derivative. Applied and Environmental Microbiology 75:504–513. https://doi.org/10.1128/AEM.01037-08

Glauser G, Gindro K, Fringeli J, De Joffrey JP, Rudaz S, Wolfender J-L (2009) Differential Analysis of Mycoalexins in Confrontation Zones of Grapevine Fungal Pathogens by Ultrahigh Pressure Liquid Chromatography / Time-of-Flight Mass Spectrometry and Capillary Nuclear Magnetic Resonance. Journal of Agricultural and Food Chemistry 57:1127–1134. https://doi.org/10.1021/jf8033539

Hiscox J, Baldrian P, Rogers HJ, Boddy L (2010) Changes in oxidative enzyme activity during interspecific mycelial interactions involving the white-rot fungus Trametes versicolor. Fungal Genetics and Biology 47:562–571. https://doi.org/10.1016/j.fgb.2010.03.007

Hoefler BC, Gorzelnik K V, Yang JY, Hendricks N, Dorrestein PC, Straight PD (2012) Enzymatic resistance to the lipopeptide surfactin as identified through imaging mass spectrometry of bacterial competition. Proceedings of the National Academy of Sciences 109:13082–13087. https://doi.org/10.1073/pnas.1205586109

Hoffman LR, Deziel E, D'Argenio DA, Lepine F, Emerson J, McNamara S, Gibson RL, Ramsey BW, Miller SI (2006) Selection for Staphylococcus aureus small-colony variants due to growth in the presence of Pseudomonas aeruginosa. Proceedings of the National Academy of Sciences 103:19890–19895. https://doi.org/10.1073/pnas.0606756104

Hoshino S, Okada M, Wakimoto T, Zhang H, Hayashi F, Onaka H, Abe I (2015a) Niizalactams A-C, Multicyclic Macrolactams Isolated from Combined Culture of Streptomyces with Mycolic Acid-Containing Bacterium. Journal of Natural Products 78:3011–3017. https://doi.org/10.1021/acs.jnatprod.5b00804

Hoshino S, Wakimoto T, Onaka H, Abe I (2015b) Chojalactones A-C, cytotoxic butanolides isolated from streptomyces sp. cultivated with mycolic acid containing bacterium. Organic Letters 17:1501–1504. https://doi.org/10.1021/acs.orglett.5b00385

Hoshino S, Zhang L, Awakawa T, Wakimoto T, Onaka H, Abe I (2015c) Arcyriaflavin E, a new cytotoxic indolocarbazole alkaloid isolated by combined-culture of mycolic acid-containing bacteria and Streptomyces cinnamoneus NBRC 13823. Journal of Antibiotics 68:342–344

Huang S, Ding W, Li C, Cox DG (2014) Two new cyclopeptides from the co-culture broth of two marine mangrove fungi and their antifungal activity. Pharmacogn Mag 10:410–414. https://doi.org/10.4103/0973-1296.141781

Huynh T-H, Lee J, Moon DH, Nguyen TQ, Son S, Hwang S, Du YE, Cui J, Jang J-H, Nam S-J, Shin J, Jang J, Lee SK, Oh K-B, Oh D-C (2022) Gwanakosides A and B, 6-Deoxy-α-l-talopyranose-Bearing Aromatic Metabolites from a Streptomyces sp. and Coculture with Pandoraea sp. J Nat Prod 85:83–90. https://doi.org/10.1021/acs.jnatprod.1c00703

Hwang GJ, Jang M, Son S, Kim GS, Lee B, Heo KT, Kim GJ, Choi H, Hur J-S, Jang J-P, Ko S-K, Hong Y-S, Ahn JS, Jang J-H (2022) Ulleungdolin, a Polyketide–Peptide Hybrid Bearing a 2,4-Di-O-methyl-β-d-antiarose from Streptomyces sp. 13F051 Co-cultured with Leohumicola minima 15S071. J Nat Prod 85:2445–2453. https://doi.org/10.1021/acs.jnatprod.2c00682

Hynes J, Müller CT, Jones TH, Boddy L (2007) Changes in Volatile Production During the Course of Fungal Mycelial Interactions Between Hypholoma fasciculare and Resinicium bicolor. Journal of Chemical Ecology 33:43–57. https://doi.org/10.1007/s10886-006-9209-6

Iakovlev A, Stenlid J (2000) Spatiotemporal Patterns of Laccase Activity in Interacting Mycelia of Wood-Decaying Basidiomycete Fungi. Microbial Ecology 39:236–245. https://doi.org/10.1007/s002480000022

Joint I, Tait K, Callow ME, Callow JA, Milton D, Williams P, Cámara M (2002) Cell-to-cell communication across the prokaryote-eukaryote boundary. Science 298:1207. https://doi.org/10.1126/science.1077075

König CC, Scherlach K, Schroeckh V, Horn F, Nietzsche S, Brakhage AA, Hertweck C (2013) Bacterium Induces Cryptic Meroterpenoid Pathway in the Pathogenic Fungus Aspergillus fumigatus. ChemBioChem 14:938–942. https://doi.org/10.1002/cbic.201300070

Konstantinidis D, Pereira F, Geissen E-M, Grkovska K, Kafkia E, Jouhten P, Kim Y, Devendran S, Zimmermann M, Patil KR (2021) Adaptive laboratory evolution of microbial co-cultures for improved metabolite secretion. Molecular Systems Biology 17:e10189. https://doi.org/10.15252/msb.202010189

Kos B, Beganović J, Jurašić L, Švađumović M (2011) Coculture-inducible bacteriocin biosynthesis of different probiotic strains by dairy starter culture Lactococcus lactis. Mljekarstvo 61:273–282

Krespach MKC, Stroe MC, Netzker T, Rosin M, Zehner LM, Komor AJ, Beilmann JM, Krüger T, Kniemeyer O, Schroeckh V, Hertweck C, Brakhage AA (2022) Ubiquitous bacterial polyketides induce cross-kingdom microbial interactions. bioRxiv 2022.05.09.491136. https://doi.org/10.1101/2022.05.09.491136

Kreth J, Merritt J, Shi W, Qi F (2005) Competition and Coexistence between Streptococcus mutans and Streptococcus sanguinis in the Dental Biofilm Competition and Coexistence between Streptococcus mutans and Streptococcus sanguinis in the Dental Biofilm. Journal of Bacteriology 187:7193–7203. https://doi.org/10.1128/JB.187.21.7193

Kurosawa K, Ghiviriga I, Sambandan TG, Lessard PA, Barbara JE, Rha C, Sinskey AJ (2008) Rhodostreptomycins, antibiotics biosynthesized following horizontal gene transfer from Streptomyces padanus to Rhodococcus fascians. Journal of the American Chemical Society 130:1126–1127. https://doi.org/10.1021/ja077821p

Li C, Wang J, Luo C, Ding W, Cox DG (2014) A new cyclopeptide with antifungal activity from the co-culture broth of two marine mangrove fungi. Natural Product Research 28:616–621. https://doi.org/10.1080/14786419.2014.887074

Li C, Zhang J, Shao C, Ding W, She Z, Lin J (2011) A New Xanthone Derivative from the co-culture broth of two marine fungi (strain No. E33 and K38). Chemistry of Natural Compounds 47:342–344

Li CY, Ding WJ, Shao CL, She ZG, Lin Y-C (2010) A new diimide derivative from the co-culture broth of two mangrove fungi (strain (strains nos. E33 and K38). Journal of Asian Natural Products Research 12:809–813. https://doi.org/10.1007/s10600-013-0751-5

Liang L, Sproule A, Haltli B, Marchbank DH, Berrué F, Overy DP, McQuillan K, Lanteigne M, Duncan N, Correa H, Kerr RG (2019) Discovery of a New Natural Product and a Deactivation of a Quorum

Sensing System by Culturing a “Producer” Bacterium With a Heat-Killed “Inducer” Culture. Frontiers in Microbiology 9:3351

Lim H-J, An JS, Bae ES, Cho E, Hwang S, Nam S-J, Oh K-B, Lee SK, Oh D-C (2022) Ligiamycins A and B, Decalin-Amino-Maleimides from the Co-Culture of Streptomyces sp. and Achromobacter sp. Isolated from the Marine Wharf Roach, Ligia exotica. Marine Drugs 20. https://doi.org/10.3390/md20020083

Liu C, Hu B, Liu Y, Chen S (2006) Stimulation of nisin production from whey by a mixed culture of Lactococcus lactis and Saccharomyces cerevisiae. Applied Biochemistry and Biotechnology 131:751–761. https://doi.org/10.1385/ABAB:131:1:751

Liu H, Hao D, Li Y, Wang X, Chen J (2022a) Approaches for the establishment of optimized co-culture system of multiple Trichoderma strains for culture metabolites highly effective in cucumber growth promotion. Frontiers in Microbiology 13

Liu Z, Fu B, Duan X, Lv W, Kang S, Zhou M, Wang C, Li D, Xu N (2022b) Effects of cell-cell interactions between A. oryzae and Z. rouxii on morphology and secondary metabolites. LWT 170:114035. https://doi.org/10.1016/j.lwt.2022.114035

Liu Z, Kang B, Duan X, Hu Y, Li W, Wang C, Li D, Xu N (2022c) Metabolomic profiles of the liquid state fermentation in co-culture of A. oryzae and Z. rouxii. Food Microbiology 103:103966. https://doi.org/10.1016/j.fm.2021.103966

Long RA, Azam F (2001) Antagonistic interactions among marine pelagic bacteria. Applied and Environmental Microbiology 67:4975. https://doi.org/10.1128/AEM.67.11.4975

Lopez-Medina E, Fan D, Coughlin LA, Ho EX, Lamont IL, Reimmann C, Hooper L V, Koh AY (2015) Candida albicans Inhibits Pseudomonas aeruginosa Virulence through Suppression of Pyochelin and Pyoverdine Biosynthesis. PLoS Pathogens 11:1–34. https://doi.org/10.1371/journal.ppat.1005129

Maimone NM, de Oliveira LFP, Santos SN, de Lira SP (2021) Elicitation of Streptomyces lunalinharesii secondary metabolism through co-cultivation with Rhizoctonia solani. Microbiological Research 251:126836. https://doi.org/10.1016/j.micres.2021.126836

Maldonado A, Ruiz-Barba JL, Jiménez-Díaz R (2004) Production of plantaricin NC8 by Lactobacillus plantarum NC8 is induced in the presence of different types of gram-positive bacteria. Archives of Microbiology 181:8–16. https://doi.org/10.1007/s00203-003-0606-8

Man LL, Meng XC, Zhao RH (2012) Induction of plantaricin MG under co-culture with certain lactic acid bacterial strains and identification of LuxS mediated quorum sensing system in Lactobacillus plantarum KLDS1.0391. Food Control 23:462–469. https://doi.org/10.1016/j.foodcont.2011.08.015

McAlester G, O’Gara F, Morrissey JP (2008) Signal-mediated interactions between Pseudomonas aeruginosa and Candida albicans. Journal of Medical Microbiology 57:563–569. https://doi.org/10.1099/jmm.0.47705-0

Mearns-Spragg A, Bregu M, Boyd KG, Burgess JG (1998) Cross-species induction and enhancement of antimicrobial activity produced by epibiotic bacteria from marine algae and invertebrates, after exposure to terrestrial bacteria. Letters in applied microbiology 27:142–146. https://doi.org/10.1046/j.1472-765X.1998.00416.x

Menezes RC, Kai M, Krause K, Matthäus C, Svatoš A, Popp J, Kothe E (2015) Monitoring metabolites from Schizophyllum commune interacting with Hypholoma fasciculare combining LESA-HR mass spectrometry and Raman microscopy. Analytical and Bioanalytical Chemistry 407:2273–2282. https://doi.org/10.1007/s00216-014-8383-6

Meng LH, Liu Y, Li XM, Xu GM, Ji NY, Wang BG (2015) Citrifelins A and B, Citrinin Adducts with a Tetracyclic Framework from Cocultures of Marine-Derived Isolates of Penicillium citrinum and Beauveria felina. Journal of Natural Products 78:2301–2305. https://doi.org/10.1021/acs.jnatprod.5b00450

Meyer V, Stahl U (2003) The influence of co-cultivation on expression of the antifungal protein in Aspergillus giganteus. Journal of Basic Microbiology 43:68–74. https://doi.org/10.1002/jobm.200390007

Minerdi D, Bossi S, Gullino ML, Garibaldi A (2009) Volatile organic compounds: A potential direct long-distance mechanism for antagonistic action of Fusarium oxysporum strain MSA 35. Environmental Microbiology 11:844–854. https://doi.org/10.1111/j.1462-2920.2008.01805.x

Moree WJ, Phelan V V, Wu C-H, Bandeira N, Cornett DS, Duggan BM, Dorrestein PC (2012) Interkingdom metabolic transformations captured by microbial imaging mass spectrometry. Proceedings of the National Academy of Sciences 109:13811–13816. https://doi.org/10.1073/pnas.1206855109

Moree WJ, Yang JY, Zhao X, Liu WT, Gutiérrez M, Dorrestein PC, Aparicio M, Atencio L, Ballesteros J, Sánchez J, Gavilán RG, Gutiérrez M, Dorrestein PC (2013) IMAGING MASS SPECTROMETRY OF A CORAL MICROBE INTERACTION WITH FUNGI. Journal of Chemical Ecology 39:1–14. https://doi.org/10.1007/s10886-013-0320-1.IMAGING

Moussa M, Ebrahim W, Bonus M, Gohlke H, Mándi A, Kurtán T, Hartmann R, Kalscheuer R, Lin W, Liu Z, Proksch P (2019) Co-culture of the fungus Fusarium tricinctum with Streptomyces lividans induces production of cryptic naphthoquinone dimers. RSC Advances 9:1491–1500. https://doi.org/10.1039/C8RA09067J

Moussa M, Ebrahim W, Kalscheuer R, Liu Z, Proksch P (2020) Co-culture of the bacterium Pseudomonas aeruginosa with the fungus Fusarium tricinctum induces bacterial antifungal and quorum sensing signaling molecules. Phytochemistry Letters 36:37–41. https://doi.org/10.1016/j.phytol.2020.01.013

Müller MEH, Steier I, Köppen R, Siegel D, Proske M, Korn U, Koch M (2012) Cocultivation of phytopathogenic Fusarium and Alternaria strains affects fungal growth and mycotoxin production. Journal of Applied Microbiology 113:874–887. https://doi.org/10.1111/j.1365-2672.2012.05388.x

Ninomiya A, Urayama S, Hagiwara D (2022) Antibacterial diphenyl ether production induced by co-culture of Aspergillus nidulans and Aspergillus fumigatus. Applied Microbiology and Biotechnology 106:4169–4185. https://doi.org/10.1007/s00253-022-11964-5

Nonaka K, Abe T, Iwatsuki M, Mori M, Yamamoto T, Shiomi K, Ômura S, Masuma R (2011) Enhancement of metabolites productivity of Penicillium pinophilum FKI-5653, by co-culture with Trichoderma harzianum FKI-5655. Journal of Antibiotics 64:769–774. https://doi.org/10.1038/ja.2011.91

Notz R, Maurhofer M, Dubach H (2002) Fusaric acid-producing strains of Fusarium oxysporum alter 2, 4-diacetylphloroglucinol biosynthetic gene expression in Pseudomonas fluorescens CHA0 in vitro and. Applied and Environmental Microbiology 68:2229–2235. https://doi.org/10.1128/AEM.68.5.2229

Oh DC, Jensen PR, Kauffman CA, Fenical W (2005) Libertellenones A-D: Induction of cytotoxic diterpenoid biosynthesis by marine microbial competition. Bioorganic and Medicinal Chemistry 13:5267–5273. https://doi.org/10.1016/j.bmc.2005.05.068

Oh DC, Kauffman CA, Jensen PR, Fenical W (2007) Induced production of emericellamides A and B from the marine-derived fungus Emericella sp. in competing co-culture. Journal of Natural Products 70:515–520. https://doi.org/10.1021/np060381f

Ojika M, Molli SD, Kanazawa H, Yajima A, Toda K, Nukada T, Mao H, Murata R, Asano T, Qi J, Sakagami Y (2011) The second Phytophthora mating hormone defines interspecies biosynthetic crosstalk. Nature Chemical Biology 7:591–593. https://doi.org/10.1038/nchembio.617

Ola ARB, Thomy D, Lai D, Brötz-Oesterhelt H, Proksch P (2013) Inducing Secondary Metabolite Production by the Endophytic Fungus Fusarium tricinctum through Coculture with Bacillus subtilis. Journal of Natural Products 76:2094–2099. https://doi.org/10.1021/np400589h

Onaka H, Mori Y, Igarashi Y, Furumai T (2011) Mycolic acid-containing bacteria induce natural-product biosynthesis in Streptomyces species. Applied and Environmental Microbiology 77:400–406. https://doi.org/10.1128/AEM.01337-10

Oppong-Danquah E, Blümel M, Scarpato S, Mangoni A, Tasdemir D (2022) Induction of Isochromanones by Co-Cultivation of the Marine Fungus Cosmospora sp. and the Phytopathogen Magnaporthe oryzae. International Journal of Molecular Sciences 23. https://doi.org/10.3390/ijms23020782

Orban A, Jerschow JJ, Birk F, Suarez C, Schnell S, Rühl M (2023) Effect of bacterial volatiles on the mycelial growth of mushrooms. Microbiological Research 266:127250. https://doi.org/10.1016/j.micres.2022.127250

Park HB, Kwon HC, Lee CH, Yang HO (2009) Glionitrin A , an Antibiotic-Antitumor Metabolite Derived from Competitive Interaction. Journal of Natural Products 72:248–252. https://doi.org/10.1021/np800606e

Patterson GML, Bolis CM (1997) Fungal Cell-Wall Polysaccharides Elicit An Antifungal Secondary Metabolite (Phytoalexin) In The Cyanobacterium Scytonema Ocelutum2. Journal of Phycology 33:54–60. https://doi.org/doi:10.1111/j.0022-3646.1997.00054.x

Peiris D, Dunn WB, Brown M, Kell DB, Roy I, Hedger JN (2008) Metabolite profiles of interacting mycelial fronts differ for pairings of the wood decay basidiomycete fungus, Stereum hirsutum with its competitors Coprinus micaceus and Coprinus disseminatus. Metabolomics 4:52–62. https://doi.org/10.1007/s11306-007-0100-4

Peterson SB, Dunn AK, Klimowicz AK, Handelsman J (2006) Peptidoglycan from Bacillus cereus mediates commensalism with rhizosphere bacteria from the Cytophaga-Flavobacterium group. Applied and Environmental Microbiology 72:5421–5427. https://doi.org/10.1128/AEM.02928-05

Pettit RK, Pettit GR, Xu JP, Weber CA, Richert LA (2010) Isolation of human cancer cell growth inhibitory, antimicrobial lateritin from a mixed fungal culture. Planta Medica 76:500–501. https://doi.org/10.1055/s-0029-1240617

Piazentin ACM, Mendonça CMN, Vallejo M, Mussatto SI, de Souza Oliveira RP (2022) Bacteriocin-like inhibitory substances production by Enterococcus faecium 135 in co-culture with Ligilactobacillus salivarius and Limosilactobacillus reuteri. Brazilian Journal of Microbiology 53:131–141. https://doi.org/10.1007/s42770-021-00661-6

Rateb ME, Hallyburton I, Houssen WE, Bull AT, Goodfellow M, Santhanam R, Jaspars M, Ebel R (2013) Induction of diverse secondary metabolites in Aspergillus fumigatus by microbial co-culture. RSC Advances 3:14444. https://doi.org/10.1039/c3ra42378f

Rickard AH, Palmer RJ, Blehert DS, Campagna SR, Semmelhack MF, Egland PG, Bassler BL, Kolenbrander PE (2006) Autoinducer 2: A concentration-dependent signal for mutualistic bacterial biofilm growth. Molecular Microbiology 60:1446–1456. https://doi.org/10.1111/j.1365-2958.2006.05202.x

Rojo-Bezares B, Sáenz Y, Navarro L, Zarazaga M, Ruiz-Larrea F, Torres C (2007) Coculture-inducible bacteriocin activity of Lactobacillus plantarum strain J23 isolated from grape must. Food Microbiology 24:482–491. https://doi.org/10.1016/j.fm.2006.09.003

Ross C, Opel V, Scherlach K, Hertweck C (2014) Biosynthesis of antifungal and antibacterial polyketides by Burkholderia gladioli in coculture with Rhizopus microsporus. Mycoses 57:48–55. https://doi.org/10.1111/myc.12246

Ryan RP, Fouhy Y, Garcia BF, Watt SA, Niehaus K, Yang L, Tolker-Nielsen T, Dow JM (2008) Interspecies signalling via the Stenotrophomonas maltophilia diffusible signal factor influences biofilm formation and polymyxin tolerance in Pseudomonas aeruginosa. Molecular Microbiology 68:75–86. https://doi.org/10.1111/j.1365-2958.2008.06132.x

Salas-Marina MA, Silva-Flores MA, Uresti-Rivera EE, Castro-Longoria E, Herrera-Estrella A, Casas-Flores S (2011) Colonization of Arabidopsis roots by Trichoderma atroviride promotes growth and enhances systemic disease resistance through jasmonic acid/ethylene and salicylic acid pathways. European Journal of Plant Pathology 131:15–26. https://doi.org/10.1007/s10658-011-9782-6

Savoie JM, Mata G (1999) The antagonistic action of Trichoderma sp. hyphae to Lentinula edodes hyphae changes lignocellulotytic activities during cultivation in wheat straw. World Journal of Microbiology and Biotechnology 15:369–373. https://doi.org/10.1023/A:1008979701853

Schneider J, Yepes A, Garcia-Betancur JC, Westedt I, Mielich B, López D (2012) Streptomycin-induced expression in Bacillus subtilis of YtnP, a lactonase-homologous protein that inhibits development and streptomycin production in Streptomyces griseus. Applied and Environmental Microbiology 78:599–603. https://doi.org/10.1128/AEM.06992-11

Schouten A, van den Berg G, Edel-Hermann V, Steinberg C, Gautheron N, Alabouvette C, de Vos CH, Lemanceau P, Raaijmakers JM (2004) Defense responses of Fusarium oxysporum to 2,4-diacetylphloroglucinol, a broad-spectrum antibiotic produced by Pseudomonas fluorescens. Molecular plant-microbe interactions: MPMI 17:1201–1211. https://doi.org/10.1094/MPMI.2004.17.11.1201

Schroeckh V, Scherlach K, Nutzmann HW, Shelest E, Schmidt-Heck W, Schuemann J, Martin K, Hertweck C, Brakhage AA (2009) Intimate bacterial-fungal interaction triggers biosynthesis of archetypal polyketides in Aspergillus nidulans. Proceedings of the National Academy of Sciences 106:14558–14563. https://doi.org/10.1073/pnas.0901870106

Score AJ, Palfreyman JW, White NA (1997) Extracellular phenoloxidase and peroxidase enzyme production during interspecific fungal interactions. International Biodeterioration and Biodegradation 39:225–233. https://doi.org/10.1016/S0964-8305(97)00012-7

Serrano R, González-Menéndez V, Rodríguez L, Martín J, Tormo JR, Genilloud O (2017) Co-culturing of fungal strains against Botrytis cinerea as a model for the induction of chemical diversity and therapeutic agents. Frontiers in Microbiology 8:649–664. https://doi.org/10.3389/fmicb.2017.00649

Shah IM, Laaberki MH, Popham DL, Dworkin J (2008) A Eukaryotic-like Ser/Thr Kinase Signals Bacteria to Exit Dormancy in Response to Peptidoglycan Fragments. Cell 135:486–496. https://doi.org/10.1016/j.cell.2008.08.039

Sher D, Thompson JW, Kashtan N, Croal L, Chisholm SW (2011) Response of Prochlorococcus ecotypes to co-culture with diverse marine bacteria. The ISME Journal 5:1125–1132. https://doi.org/10.1038/ismej.2011.1

Shimizu H, Mizuguchi T, Tanaka E, Shioya S (1999) Nisin production by a mixed-culture system consisting of Lactococcus lactis and Kluyveromyces marxianus. Applied and Environmental Microbiology 65:3134–3141

Shin CS, Kim HJ, Kim MJ, Ju JY (1998) Morphological change and enhanced pigment production of Monascus when cocultured with Saccharomyces cerevisiae or Aspergillus oryzae. Biotechnology and Bioengineering 59:576–581. https://doi.org/10.1002/(SICI)1097-0290(19980905)59:5<576::AID-BIT7>3.0.CO;2-7

Shin D, Byun WS, Moon K, Kwon Y, Bae M, Um S, Lee SK, Oh DC (2018) Coculture of Marine Streptomyces sp. With Bacillus sp. Produces a New Piperazic Acid-Bearing Cyclic Peptide. Frontiers in Chemistry 6:498. https://doi.org/10.3389/fchem.2018.00498

Slattery M, Rajbhandari I, Wesson K (2001) Competition-Mediated Antibiotic Induction in the Marine Bacterium Streptomyces tenjimariensis. Microbial Ecology 41:90–96. https://doi.org/10.1007/978-1-60327-140-0_4

Soliman SSM, Raizada MN (2013) Interactions between co-habitating fungi elicit synthesis of Taxol from an endophytic fungus in host Taxus plants. Frontiers in Microbiology 4:1–14. https://doi.org/10.3389/fmicb.2013.00003

Sonnenbichler J, Dietrich J, Peipp H (1994) Secondary Fungal Metabolites and Their Biological Activities, V. Investigations Concerning the Induction of the Biosynthesis of Toxic Secondary Metabolites in Basidiomycetes. Biological Chemistry Hoppe-Seyler 375:71–80. https://doi.org/10.1515/bchm3.1994.375.1.71

Stierle AA, Stierle DB, Decato D, Alverson J, Apedaile L (2021) Cryptic Biosynthesis of the Berkeleypenostatins from Coculture of Extremophilic Penicillium sp. J Nat Prod 84:1656–1665. https://doi.org/10.1021/acs.jnatprod.1c00248

Stierle AA, Stierle DB, Decato D, Priestley ND, Alverson JB, Hoody J, McGrath K, Klepacki D (2017) The Berkeleylactones, Antibiotic Macrolides from Fungal Coculture. Journal of Natural Products 80:1150–1160. https://doi.org/10.1021/acs.jnatprod.7b00133

Straight PD, Willey JM, Kolter R (2006) Interactions between Streptomyces coelicolor and Bacillus subtilis: Role of surfactants in raising aerial structures. Journal of Bacteriology 188:4918–4925. https://doi.org/10.1128/JB.00162-06

Stroe MC, Netzker T, Scherlach K, Krüger T, Hertweck C, Valiante V, Brakhage AA (2020) Targeted induction of a silent fungal gene cluster encoding the bacteria-specific germination inhibitor fumigermin. eLife 9:1–20. https://doi.org/10.7554/eLife.52541

Sun Y, Shi X, Xing Y, Ren X-X, Zhang D-Y, Li X, Xiu Z-L, Dong Y-S (2022) Co-culture of Aspergillus sydowii and Bacillus subtilis induces the production of antibacterial metabolites. Fungal Biology 126:320–332. https://doi.org/10.1016/j.funbio.2022.01.002

Sung AA, Gromek SM, Balunas MJ (2017) Upregulation and identification of antibiotic activity of a marine-derived Streptomyces sp. via co-cultures with human pathogens. Marine Drugs 15. https://doi.org/10.3390/md15080250

Tabasco R, García-Cayuela T, Peláez C, Requena T (2009) Lactobacillus acidophilus La-5 increases lactacin B production when it senses live target bacteria. International Journal of Food Microbiology 132:109–116. https://doi.org/10.1016/j.ijfoodmicro.2009.04.004

Tada S, Katakura Y, Ninomiya K, Shioya S (2007) Fed-batch coculture of Lactobacillus kefiranofaciens with Saccharomyces cerevisiae for effective production of kefiran. Journal of Bioscience and Bioengineering 103:557–562. https://doi.org/10.1263/jbb.103.557

Tata A, Perez C, Campos ML, Bayfield MA, Eberlin MN, Ifa DR (2015) Imprint Desorption Electrospray Ionization Mass Spectrometry Imaging for Monitoring Secondary Metabolites Production during Antagonistic Interaction of Fungi. Analytical Chemistry 87:12298–12305. https://doi.org/10.1021/acs.analchem.5b03614

Tian Y, Abdallah MF, De Boevre M, Audenaert K, Wang C, De Saeger S, Wu A (2023) Deciphering Alternaria metabolic responses in microbial confrontation via an integrated mass spectrometric targeted and non-targeted strategy. Food Chemistry 404:134694. https://doi.org/10.1016/j.foodchem.2022.134694

Tong H, Chen W, Merritt J, Qi F, Shi W, Dong X (2007) Streptococcus oligofermentans inhibits Streptococcus mutans through conversion of lactic acid into inhibitory H2O2: A possible counteroffensive strategy for interspecies competition. Molecular Microbiology 63:872–880. https://doi.org/10.1111/j.1365-2958.2006.05546.x

Traxler MF, Watrous JD, Alexandrov T, Dorrestein PC, Kolter R (2013) Interspecies interactions stimulate diversification of the Streptomyces coelicolor secreted metabolome. mBio 4:459–13. https://doi.org/10.1128/mBio.00459-13

Trischman JA, Oeffner RE, De Luna MG, Kazaoka M (2004) Competitive induction and enhancement of indole and a diketopiperazine in marine bacteria. Marine Biotechnology 6:215–220. https://doi.org/10.1007/s10126-003-0010-z

Turpin PE, Dhir VK, Maycroft KA, Rowland C, Wllington EMH (1992) The effect of Streptomycetes species on the survival of Salmonella in soil. FEMS Microbiology Ecology 101:271–280

Ueda K, Kawai S, Ogawa H, Kiyama A, Kubota T, Kawanobe H, Beppu T (2000) Wide Distribution of Interspecific Stimulatory Events on Antibiotic Production and Sporulation among Streptomyces species. The Journal of antibiotics 53:979–982. https://doi.org/11099234

van Rij ET, Girard G, Lugtenberg BJJ, Bloemberg G V (2005) Influence of fusaric acid on phenazine-1-carboxamide synthesis and gene expression of Pseudomonas chlororaphis strain PCL1391. Microbiology 151:2805–2814. https://doi.org/10.1099/mic.0.28063-0

Vásquez-Bonilla JN, Barranco-Florido JE, Ponce-Alquicira E, Rincón-Guevara MA, Loera O (2022) Improvement of beauvericin production by Fusarium oxysporum AB2 under solid-state fermentation using an optimised liquid medium and co-cultures. Mycotoxin Research 38:175–183. https://doi.org/10.1007/s12550-022-00458-y

Vega NM, Allison KR, Samuels AN, Klempner MS, Collins JJ (2013) Salmonella typhimurium intercepts Escherichia coli signaling to enhance antibiotic tolerance. Proceedings of the National Academy of Sciences 110:14420–14425. https://doi.org/10.1073/pnas.1308085110

Vinale F, Nicoletti R, Borrelli F, Mangoni A, Parisi OA, Marra R, Lombardi N, Lacatena F, Grauso L, Finizio S, Lorito M, Woo SL (2017) Co-Culture of Plant Beneficial Microbes as Source of Bioactive Metabolites. Scientific Reports 7:1–12. https://doi.org/10.1038/s41598-017-14569-5

Wang J, Ding W, Li C, Huang S, She Z, Lin Y (2013a) A New Polusubstituted Benzaldehyde from the co-culture broth of two marine fungi (strains Nos. E33 and K38). Chemistry of Natural Compounds 49:689–691

Wang J, Lin W, Wray V, Lai D, Proksch P (2013b) Induced production of depsipeptides by co-culturing Fusarium tricinctum and Fusarium begoniae. Tetrahedron Letters 54:2492–2496. https://doi.org/10.1016/j.tetlet.2013.03.005

Wang LH, He Y, Gao Y, Wu JE, Dong YH, He C, Wang SX, Weng LX, Xu JL, Tay L, Fang RX, Zhang LH (2004) A bacterial cell-cell communication signal with cross-kingdom structural analogues. Molecular Microbiology 51:903–912. https://doi.org/10.1046/j.1365-2958.2003.03883.x

Wang Y, Glukhov E, He Y, Liu Y, Zhou L, Ma X, Hu X, Hong P, Gerwick WH, Zhang Y (2022) Secondary Metabolite Variation and Bioactivities of Two Marine Aspergillus Strains in Static Co-Culture Investigated by Molecular Network Analysis and Multiple Database Mining Based on LC-PDA-MS/MS. Antibiotics 11. https://doi.org/10.3390/antibiotics11040513

Watanabe T, Izaki K, Takahashi H (1982) New Polyenic Antibiotics Against Gram-positive and -Negative Bacteria. The Journal of Antibiotics 35:1141–1147

Watsuji T, Yamada S, Yamabe T, Watanabe Y, Kato T, Saito T, Ueda K, Beppu T (2007) Identification of indole derivatives as self-growth inhibitors of Symbiobacterium thermophilum, a unique bacterium whose growth depends on coculture with a Bacillus sp. Applied and Environmental Microbiology 73:6159–6165. https://doi.org/10.1128/AEM.02835-06

White N, Boddy L (1992) Extracellular enzyme localization during interspecific fungal interactions. FEMS Microbiology Letters 98:75–80. https://doi.org/10.1111/j.1574-6968.1992.tb05493.x

Whitt J, Shipley SM, Newman DJ, Zuck KM (2014) Tetramic acid analogues produced by coculture of saccharopolyspora erythraea with fusarium pallidoroseum. Journal of Natural Products 77:173–177. https://doi.org/10.1021/np400761g

Wu C, Zacchetti B, Ram AFJ, Van Wezel GP, Claessen D, Choi YH (2015) Expanding the chemical space for natural products by Aspergillus-Streptomyces co-cultivation and biotransformation. Scientific Reports 5:1–10. https://doi.org/10.1038/srep10868

Wu Y-M, Yang X-Q, Chen J-X, Wang T, Li T-R, Liao F-R, Liu R-T, Yang Y-B, Ding Z-T (2022) A new butenolide with antifungal activity from solid co-cultivation of Irpex lacteus and Nigrospora oryzae. Natural Product Research 1–5. https://doi.org/10.1080/14786419.2022.2037589

Xu XL, Lee RTH, Fang HM, Wang YM, Li R, Zou H, Zhu Y, Wang Y (2008) Bacterial Peptidoglycan Triggers Candida albicans Hyphal Growth by Directly Activating the Adenylyl Cyclase Cyr1p. Cell Host and Microbe 4:28–39. https://doi.org/10.1016/j.chom.2008.05.014

Yakovleva EP, Bulgakova TN (1978) Formation of Amphotericin B in Mixed Cultures. Pharmaceutical Chemistry Journal 12:1483–1488

Yao L, Zhu LP, Xu XY, Tan LL, Sadilek M, Fan H, Hu B, Shen XT, Yang J, Qiao B, Yang S (2016) Discovery of novel xylosides in co-culture of basidiomycetes Trametes versicolor and Ganoderma applanatum by integrated metabolomics and bioinformatics. Scientific Reports 6:1–13. https://doi.org/10.1038/srep33237

Yu G, Sun P, Aierken R, Sun C, Zhang Z, Che Q, Zhang G, Zhu T, Gu Q, Li M, Li D (2022) Linear polyketides produced by co-culture of Penicillium crustosum and Penicillium fellutanum. Marine Life Science & Technology 4:237–244. https://doi.org/10.1007/s42995-021-00125-8

Yu L, Ding W, Wang Q, Ma Z, Xu X, Zhao X, Chen Z (2017) Induction of cryptic bioactive 2,5-diketopiperazines in fungus Penicillium sp. DT-F29 by microbial co-culture. Tetrahedron 73:907–914. https://doi.org/10.1016/j.tet.2016.12.077

Zhang H, Stephanopoulos G (2016) Co-culture engineering for microbial biosynthesis of 3-amino-benzoic acid in Escherichia coli. Biotechnology Journal 11:981–987. https://doi.org/10.1002/biot.201600013

Zhao X, Hou D, Xu J, Wang K, Hu Z (2022) Antagonistic Activity of Fungal Strains against Fusarium Crown Rot. Plants 11. https://doi.org/10.3390/plants11030255

Zhu F, Chen G, Chen X, Huang M, Wan X (2011) Aspergicin, a new antibacterial alkaloid produced by mixed fermentation of two marine-derived mangrove epiphytic fungi. Chemistry of Natural Compounds 47:767–769. https://doi.org/10.1007/s10600-011-0053-8

Zhu F, Chen GY, Wu JS, Pan JH (2013) Structure revision and cytotoxic activity of marinamide and its methyl ester, novel alkaloids produced by co-cultures of two marine-derived mangrove endophytic fungi. Natural Product Research 27:1960–1964. https://doi.org/10.1080/14786419.2013.800980

Zhu F, Lin Y, Ding J, Wang X, Huang L (2007) Secondary metabolites of two marine-derived mangrove endophytic fungi (strain nos. 1924# and 3893#) by mixed fermentation. Chemistry and Industry of Forest Products 27:8–20

Zhuravleva OI, Belousova EB, Oleinikova GK, Antonov AS, Khudyakova YV, Rasin AB, Popov RS, Menchinskaya ES, Trinh PTH, Yurchenko AN, Yurchenko EA (2022) Cytotoxic Drimane-Type Sesquiterpenes from Co-Culture of the Marine-Derived Fungi Aspergillus carneus KMM 4638 and Beauveria felina (=Isaria felina) KMM 4639. Marine Drugs 20:584. https://doi.org/10.3390/md20090584

Zuck KM, Shipley S, Newman DJ (2011) Induced Production of N-Formyl Alkaloids from Aspergillus fumigatus by Co-culture with Streptomyces peucetius. Journal of Natural Products 74:1653–1657. https://doi.org/10.1021/np200255f
